# Deltex E3 ubiquitin ligase 2 potentiates STING-mediated type I interferon response by K63-linked ubiquitination

**DOI:** 10.1038/s41419-026-08659-4

**Published:** 2026-03-28

**Authors:** Zhuang Liu, Runze Li, Caihong Fan, Yuheng Liu, Jia Liu, Mingchen Yin, Ni Shang, Xudong Wang, Zhi Qi, Yanna Shen, Chang Liu

**Affiliations:** 1 Department of Head and Neck Oncology, Tianjin Medical University Cancer Institute & Hospital, Key Laboratory of Basic and Translational Medicine on Head & Neck Cancer, Tianjin; National Clinical Research Center for Cancer; Tianjin’s Clinical Research Center for Cancer; Key Laboratory of Cancer Prevention and Therapy, Tianjin; Tianjin Medical University, Tianjin, China; 2 Division of Medical Technology, Tianjin Medical University, Tianjin, China; 3 Department of Molecular Pharmacology, School of Medicine; Beichen Hospital, Nankai University, Tianjin, China; 4 Tianjin Key Laboratory of General Surgery in Construction, Tianjin Union Medical Center, Tianjin, China; 5 The first Department of Critical Care Medicine, The first Affiliated Hospital of Shihezi University, Shihezi, China

**Keywords:** Cell signalling, Ubiquitylation

## Abstract

Stimulator of interferon genes (STING) signaling, as a pivotal DNA-sensing mechanism, orchestrates antiviral and antitumor immunity through the induction of type I interferon response. Precise modulation of STING signaling is critical for maintaining immune homeostasis, yet its regulatory landscape has not been fully elucidated. Here, we identify the Deltex E3 ubiquitin ligase 2 (DTX2) as a positive regulator of STING-type I interferon response. Loss of *Dtx2* in mouse macrophages and embryonic fibroblasts (MEFs) markedly impairs the type I interferon production upon double-stranded DNA (dsDNA) or cyclic guanosine monophosphate (GMP)-adenosine monophosphate (AMP) (cGAMP) stimulation. Correspondingly, *Dtx2*^−/−^ mice exhibit more susceptibility to DNA viral infection compared to its counterparts. Mechanistically, DTX2 interacts with STING to promote K63-linked ubiquitination at residue K236 and K370, which facilitates the translocation of STING from the endoplasmic reticulum (ER) to the Golgi apparatus and activates downstream signaling cascades. Furthermore, we demonstrate that DTX2 potentiates STING-mediated type I interferon response in multiple tumor cell lines, and enhances anti-tumor immunity in murine head and neck cancer models. Collectively, our work uncovers DTX2 as a previously unrecognized regulator of STING, revealing a ubiquitin-dependent mechanism for fine-tuning innate immune response with implications for combating infections and cancer.

## Introduction

The innate immune system, a foundational and non-specific defense mechanism, is initiated by the recognition of foreign or misplaced double-stranded DNA (dsDNA). Such dsDNA originates from pathogen infections like herpes simplex virus (HSV) or tissue damage, and is recognized by a repertoire of intracellular pattern recognition receptors termed DNA sensors [1, 2]. A key player in this recognition is cyclic GMP-AMP synthase (cGAS), which typically resides in an inactive state within cells. Upon binding to dsDNA, cGAS becomes activated and catalyzes the production of a second messenger cyclic guanosine monophosphate (GMP)-adenosine monophosphate (AMP) (cGAMP) [3, 4]. cGAMP subsequently binds to the endoplasmic reticulum (ER)-resident adaptor protein stimulator of interferon genes (STING), triggering its oligomerization and trafficking from the ER to the Golgi apparatus. Upon trafficking to the Golgi, STING recruits and activates TANK-binding kinase 1 (TBK1), followed by interferon regulatory factor 3 (IRF3) and NF-κB subunit p65, which ultimately led to the production of type I interferon and inflammatory factors [5]. This spatial regulation of STING trafficking is critical for proper signal transduction [6]. Beyond its role in antiviral defense, the STING-mediated type I interferon response is integral to anti-tumor immunity. Type I interferons, produced by macrophages, dendritic cells (DCs), or even tumor cells, shape the tumor microenvironment by fostering the activation and cross-infiltration of immune cells [7, 8]. Nonetheless, aberrant STING activation, particularly when triggered by self-DNA, can precipitate severe autoimmune and inflammatory conditions [9]. Consequently, precise modulation of STING signaling is crucial for fostering antiviral and anti-tumor immunity as well as preventing autoimmunity. Despite recent advances, the STING signal regulation mechanism is not yet fully elucidated.

Ubiquitination is a common and essential post-translational modification that governs a vast array of biological processes by dictating protein stability, localization, and activity [10, 11]. Ubiquitin, a highly conserved small protein comprising 76 amino acids, can be connected to each other by seven lysine residues (K6, K11, K27, K29, K33, K48, and K63), forming distinct types of ubiquitin chains with diverse regulatory functions [12]. The ubiquitination process is mediated by a cascade of enzymes including E1 ubiquitin-activating enzyme, E2 ubiquitin-conjugating enzyme, and E3 ubiquitin-protein ligase [13]. To date, over 600 E3 ligases have been identified, and ongoing research continues to elucidate their diverse roles [14]. Several E3 ligases have been demonstrated to be critical regulators of the STING-mediated innate immune response. For instance, ring finger protein (RNF) 144A catalyzes K6-linked ubiquitination to promote STING trafficking and potentiate antiviral response [15]. Similarly, TRIM10-mediated K27/K29-linked ubiquitination facilitates STING aggregation and TBK1 recruitment, which is essential for downstream signaling [16]. Additionally, RNF115-induced K63-linked ubiquitination activates STING signaling pathway [17], whereas TRIM30α-mediated K48-linked ubiquitination targets STING for proteasomal degradation, diminishing IFN-β production [18]. These findings highlight the complex and finely tuned regulation of STING signaling by the ubiquitin system. The identification of novel E3 ligases that regulate STING signaling could provide valuable insights into the modulation of innate immune response and offer new therapeutic opportunities for diseases.

E3 ubiquitin ligases are categorized into three primary families-Really Interesting New Gene (RING), E6-AP Carboxy-terminal Homology (HECT), and Ring-in-between-Ring (RBR)-based on their domain architecture and distinctive mechanisms of substrate ubiquitination [19]. The RING family, the largest and most studied, employs its RING-finger domain to recruit E2 enzymes, directly catalyzing ubiquitin transfer to the substrate proteins [20]. Over the past decade, the Deltex (DTX) family of E3 ubiquitin ligases, which falls under the RING family and comprises DTX1, DTX2, DTX3, DTX3L, and DTX4, has emerged as a group of multifunctional E3 ligases with diverse roles in development, cell fate determination, and tumor biology [21]. DTX2, an ~67 kDa protein, is characterized by four key domains: an N-terminal WWE domain, a proline-rich region, a RING-H2 motif, and a Deltex C-terminal (DTC) domain [22]. Accumulating evidence has begun to illuminate the broad biological roles of DTX2. For instance, DTX2 inhibits myogenic differentiation through its regulation of MyoD, a pivotal myogenic regulatory factor [23]. More recently, DTX2 is identified as a novel ADP-ribosylation-dependent regulator in DNA damage repair [24]. Our research, as well as that of other investigators, indicates a compelling role for DTX2 in facilitating ferroptosis resistance in cancer [25, 26]. However, the role and mechanism of DTX2 in innate immunity remain unclear.

In this study, we identified DTX2 as a previously uncharacterized regulator of the STING-mediated type I interferon response. RNA sequencing (RNA-seq) data and gene expression analysis revealed that DTX2 expression is upregulated upon herpes simplex virus 1 (HSV-1) infection. We further demonstrated that bone marrow-derived macrophages (BMDMs), peritoneal macrophages (PMs), and embryonic fibroblasts (MEFs) from *Dtx2*^−/−^ mice exhibited significantly attenuated type I interferon response and compromised STING-downstream signaling activation upon infection with HSV-1, or stimulation with cGAMP and IFN-stimulatory DNA (ISD), relative to wild-type (WT) controls. Consistently, *Dtx2*^−/−^ mice exhibited increased susceptibility to infection by DNA viruses, compared to its counterparts. Mechanistically, we showed that DTX2 formed a stable complex with STING and promoted K63-linked ubiquitination at its lysine residues 236 and 370. This specific modification facilitated STING translocation from the ER to the Golgi apparatus, thereby enabling the efficient recruitment and activation of TBK1, IRF3, and p65. Besides, our findings revealed that DTX2 potentially augmented STING-mediated type I interferon response in a variety of tumor cell lines and potentiated anti-tumor immunity in murine head and neck cancer models. Collectively, our data unveil a novel, critical function for DTX2 in regulating STING-interferon signaling, offering new insights into the mechanisms governing antiviral and anti-tumor immunity.

## Results

### DTX2 is required for an effective type I interferon response

**Fig. 1:**
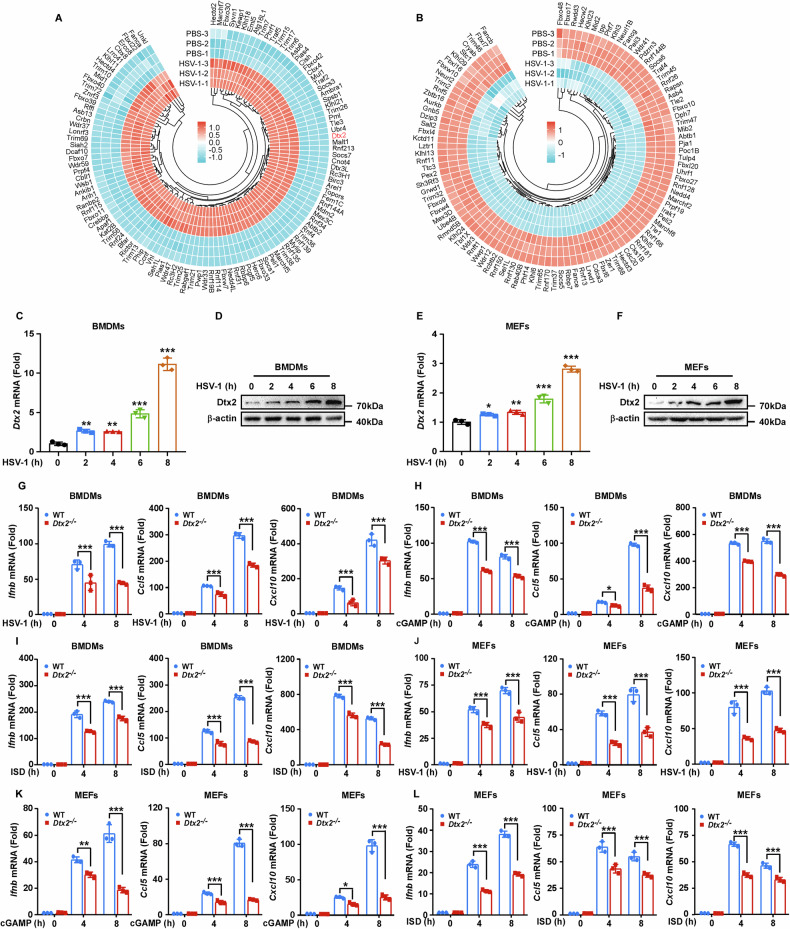
DTX2 is indispensable in facilitating an effective type I interferon response. Differentially expressed genes between HSV-1-infected and PBS-treated BMDMs were identified via RNA-seq, with significantly upregulated (**A**) and downregulated (**B**) E3 ubiquitin ligases visualized using circular heatmaps. Dtx2 expression in BMDMs infected with HSV-1 for the 0, 2, 4, 6, and 8 h was detected by RT-PCR (**C**) and western blotting (**D**). Dtx2 expression in MEFs infected with HSV-1 for the 0, 2, 4, 6, and 8 h was detected by RT-PCR (**E**) and western blotting (**F**). BMDMs were infected with HSV-1 (**G**), stimulated with cGAMP (**H**), and ISD (**I**) for the 0, 4, and 8 h. RT-PCR was used to measure the mRNA expression of *Ifnb*, *Ccl5*, and *Cxcl10*. MEFs were infected with HSV-1 (**J**), stimulated with cGAMP (**K**), and ISD (**L**) for the 0, 4, and 8 h. RT-PCR was used to measure the mRNA expression of *Ifnb*, *Ccl5*, and *Cxcl10*. Data in (**C**, **E**, **G**–**L**) are shown as mean ± SD of three independent experiments. *p < 0.05, **p < 0.01, ***p < 0.001.

To identify E3 ubiquitin ligases with potential roles in the innate immune response, we infected BMDMs with HSV-1 and conducted RNA-seq analysis. Differential expression analysis revealed 151 upregulated and 123 downregulated E3 ubiquitin ligases (Fig. 1A, B). Notably, Dtx2 expression was significantly increased following HSV-1 infection (Fig. 1A). To validate this finding, Dtx2 mRNA and protein levels were assessed in mouse BMDMs, PMs, and MEFs after HSV-1 infection. Results confirmed a significant increase in Dtx2 mRNA and protein following HSV-1 infection (Fig. 1C–F; Supplementary Fig. S1A, B).

To explore whether DTX2 potentially regulates type I interferon response, we generated *Dtx2-*deficient mice (Supplementary Fig. S1C, D). Quantitative real-time PCR (RT-PCR) and western blot analysis confirmed that Dtx2 expression was absent in BMDMs, PMs, and MEFs derived from *Dtx2*^*−/−*^ mice (Supplementary Fig. S1E–J). Subsequently, WT and *Dtx2*-deficient BMDMs and PMs were infected with HSV-1 and the transcript levels of *Ifnb*, as well as the IFN-β-inducing chemokines *Ccl5* and *Cxcl10*, was determined. RT-PCR revealed reduced *Ifnb*, *Ccl5*, and *Cxcl10* mRNA expression in *Dtx2*-deficient BMDMs and PMs compared to WT counterparts (Fig. 1G, Supplementary Fig. S1K). Consistent with these results, *Dtx2*-deficient BMDMs and PMs also exhibited reduced expression of *Ifnb*, *Ccl5*, and *Cxcl10* compared to their WT counterparts upon cGAMP treatment (Fig. 1H, Supplementary Fig. S1L) or ISD stimulation (Fig. 1I, Supplementary Fig. S1M). To further define the role of DTX2 in type I interferon response in non-immune cells, WT and *Dtx2*-deficient MEFs were subjected to HSV-1 infection, and cGAMP, or ISD stimulation. RT-PCR analysis demonstrated that the expression of *Ifnb*, *Ccl5*, and *Cxcl10* induced by HSV-1 (Fig. 1J), cGAMP (Fig. 1K), or ISD (Fig. 1L) was significantly decreased in *Dtx2*-deficient MEFs relative to WT MEFs. In parallel, to determine whether DTX2 is involved in the interferon response to RNA viruses, WT and *Dtx2*-deficient BMDMs were exposed to Sendai virus (SeV). We observed that *Dtx2* deficiency resulted in only a slight defect in interferon production following SeV infection (Supplementary Fig. S1N). Collectively, these findings suggest that *Dtx2* deficiency predominantly impairs dsDNA-induced type I interferon response.

### DTX2 regulates dsDNA-induced innate immune signaling

**Fig. 2:**
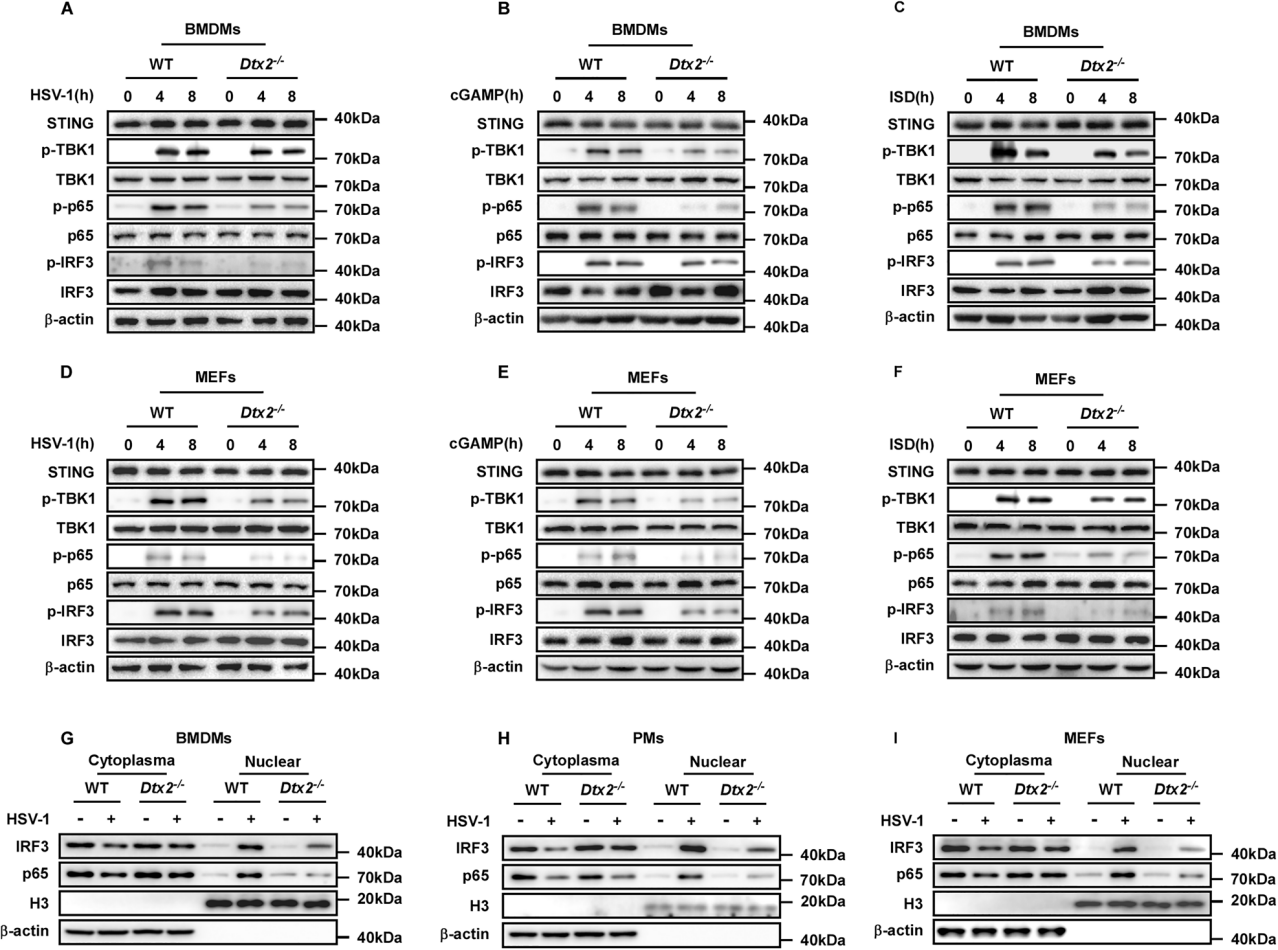
DTX2 positively regulates dsDNA-induced STING-type I interferon signaling. WT and *Dtx2*-deficient BMDMs were infected with HSV-1(**A**), stimulated with cGAMP (**B**) or ISD (**C**) for 0, 4, and 8 h. Cells were collected and lysed for western blotting. WT and *Dtx2*-deficient MEFs were infected with HSV-1 (**D**), stimulated with cGAMP (**E**), and ISD (**F**) for 0, 4, and 8 h. Cells were collected and lysed for western blotting. BMDMs (**G**), PMs (**H**), and MEFs (**I**) were infected with HSV-1 for 4 h, and then fractionated into cytosolic and nuclear subfractions for western blotting analysis.

To comprehensively delineate DTX2’s regulatory role in the STING signaling pathway, we assessed the impact of *Dtx2* deficiency on the downstream activation of STING signaling. Initially, following HSV-1 infection for 4 or 8 h, BMDMs and PMs from *Dtx2*^−/−^ mice exhibited no alteration in the total protein levels of STING, TBK1, IRF3, and p65 compared to WT controls. However, phosphorylation of TBK1 (p-TBK1), IRF3 (p-IRF3), and p65 (p-p65) was attenuated (Fig. 2A; Supplementary Fig. S2A). This phenomenon was consistently observed in *Dtx2*-deficient BMDMs and PMs stimulated with cGAMP or ISD, compared to WT controls (Fig. 2B, C; Supplementary Fig. S2B, C). To validate these observations, the above-mentioned indicators were measured in MEFs. *Dtx2*-deficient MEFs showed impairment of antiviral signaling upon HSV-1 infection, characterized by reduced p-TBK1, p-IRF3, and p-p65 (Fig. 2D). Consistent with the observations in macrophages, p-TBK1, p-IRF3, and p-p65 induced by cGAMP and ISD was reduced in *Dtx2*-deficient MEFs relative to WT MEFs (Fig. 2E, F). Given that STING signaling activation leads to IRF3 and p65 nuclear translocation, which is necessary for the expression of type I interferon and inflammatory factors such as IL-6 and TNF-α, we next investigated whether DTX2 influences IRF3 and p65 localization. As depicted in Fig. 2G–I, *Dtx2* deficiency inhibited IRF3 and p65 nuclear translocation in BMDMs, PMs, and MEFs following infection with HSV-1. Furthermore, enzyme-linked immunosorbent assay (ELISA) analysis demonstrated that *Dtx2* deficiency markedly suppressed the production of IFN-β, IL-6, and TNF-α in PMs (Supplementary Fig. S2D–F). Collectively, these findings compellingly demonstrate that DTX2 plays a crucial positive regulatory role in the DNA-induced STING signaling pathway, essential for effective activation of downstream effectors.

### DTX2 fosters the K63-linked ubiquitin chains on STING

**Fig. 3:**
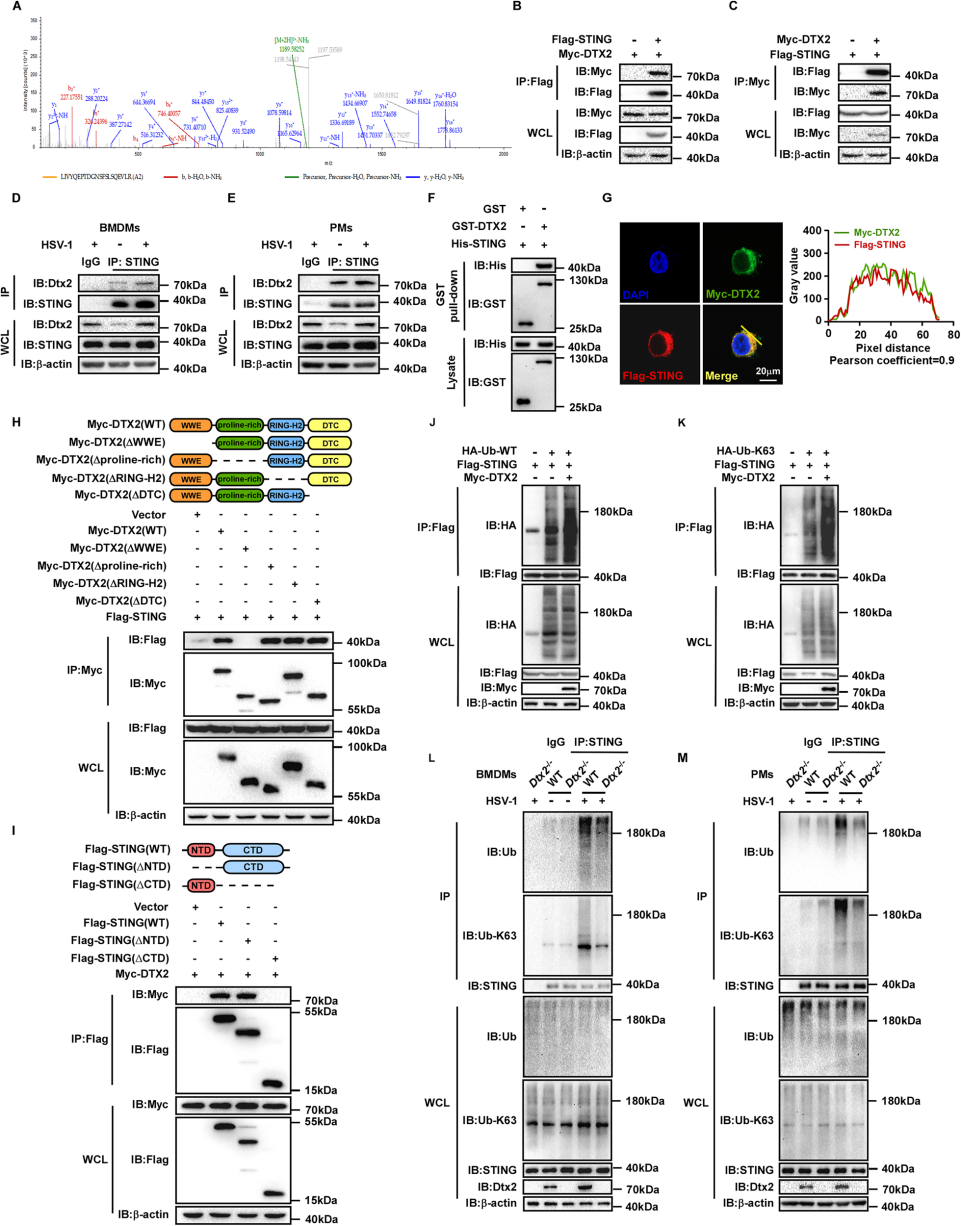
DTX2 promotes K63-linked ubiquitination of STING. **A** The peptide profile of STING identified by mass spectrometry analysis. PMs were transfected with Myc-Dtx2, infected with HSV-1 for 4 h, and then subjected to Co-IP with anti-Myc. **B**, **C** HEK293T cells were transfected with Flag-STING and Myc-DTX2 plasmids for 24 h. The cell lysate was subjected to Co-IP using anti-Flag (**B**) and anti-Myc beads (**C**), and then western blotting was performed using indicated antibodies. BMDMs (**D**) or PMs (**E**) were infected with HSV-1 for 4 h and then the cell lysate was subjected to Co-IP and western blotting to determine the protein levels of STING and Dtx2. **F** The interaction between DTX2 and STING was detected using a GST pull-down assay. **G** Fluorescence confocal images showing localization of Myc-DTX2 (green) and Flag-STING (red) in HeLa cells. Scale bar = 20 µm. HeLa cells transfected with Myc-DTX2 and Flag-STING for 24 h, then labeled with anti-Myc (green) and anti-Flag (red). Nuclei were labeled with DAPI. Co-localization between DTX2 and STING was analyzed by ImageJ software. **H** Schematic representation of the Myc-DTX2 truncations (top panel). HEK293T cells were transfected with Flag-STING and Myc-DTX2 or truncated mutants for 24 h, and the cell lysate was subjected to Co-IP using anti-Myc beads, and then western blotting was performed using indicated antibodies (bottom panel). **I** Schematic representation of the Flag-STING truncations (top panel). HEK293T cells were transfected with Myc-DTX2 and Flag-STING or truncated mutants for 24 h, and the cell lysate was subjected to Co-IP using anti-Flag beads, and western blotting was performed using indicated antibodies (bottom panel). HEK293T cells were transfected with Myc-DTX2, Flag-STING, and HA-Ub-WT (**J**) or K63 mutant (**K**) plasmids for 24 h, and STING ubiquitination was demonstrated. Cell lysate was subjected to Co-IP using anti-Flag beads, followed by western blotting using indicated antibodies. The endogenous ubiquitination and K63-ubiquitination of STING in BMDMs (**L**) or PMs (**M**) from WT and *Dtx2*^−/−^ mice were measured by Co-IP assay with anti-STING antibody, followed by western blotting with indicated antibodies.

To investigate the molecular regulation of DTX2, we sought to identify its binding partners. The Myc-tagged mouse Dtx2 overexpression plasmid was constructed and transfected into PMs, followed by Co-Immunoprecipitation (Co-IP) coupled with mass spectrometry analysis to identify DTX2-interacting proteins. Interestingly, STING protein appeared among the candidate interactors (Fig. 3A) and was consequently prioritized for further analysis. To validate the DTX2-STING interaction, we performed subsequent experiments. Initially, we co-transfected Myc-tagged DTX2 and Flag-tagged STING into HEK293T cells, followed by reciprocal Co-IP assays, which indicated a physical association (Fig. 3B, C). Then, endogenous Co-IP demonstrated that Dtx2 and STING formed a complex in both BMDMs and PMs following HSV-1 infection (Fig. 3D, E). Furthermore, a GST pull-down assay demonstrated a direct interaction between DTX2 and STING (Fig. 3F). Consistently, confocal microscopy analysis revealed the co-localization of DTX2 and STING (Fig. 3G). These data collectively establish that DTX2 interacts with STING. To determine which domain of DTX2 is critical for STING interaction, we generated a series of Myc-tagged DTX2 truncation mutants, encompassing deletions of WWE, proline-rich, RING-H2, and DTC domains. Co-IP assays revealed that STING interacted with full-length DTX2 and with mutants lacking the proline-rich, RING-H2, and DTC domains, but not with the WWE domain deletion mutant (Fig. 3H). These findings unequivocally implicate the DTX2 WWE domain as essential for STING binding. STING itself consists of an N-terminal domain and a C-terminal domain. Subsequent investigations aimed to examined which domain of STING is responsible for its interaction with DTX2. Co-IP experiments illustrated that removal of the STING C-terminal domain significantly disrupted the interaction with DTX2 (Fig. 3I), indicating the C-terminal domain of STING as the key mediator of its interaction with DTX2.

Since DTX2 is an E3 ubiquitin ligase that interacts with STING, we investigated its role in regulating STING ubiquitination. HEK293T cells were co-transfected with Flag-tagged STING, HA-tagged ubiquitin (WT), and Myc-tagged DTX2. As illustrated in Fig. 3J, DTX2 overexpression markedly enhanced the ubiquitination of STING. E3 ubiquitin ligases have been found to orchestrate substrate function through catalyzing diverse types of ubiquitination. To determine which lysine-linked ubiquitin was hired by DTX2 to modulate the function of STING, we utilized a series of ubiquitin mutants, each possessing only one specific lysine available for linkage (K6, K11, K27, K29, K33, K48, and K63). Our findings revealed that overexpression of DTX2 predominantly increased K63-linked ubiquitination of STING, while exhibiting no significant effect on other ubiquitin linkages (Fig. 3K; Supplementary Fig. S3A–F). To further confirm this, we assessed endogenous STING ubiquitination in *Dtx2*-deficient BMDMs and PMs following HSV-1 infection. As depicted in Fig. 3L, M, STING exhibited substantial both total (WT) and K63-linked ubiquitination in BMDMs and PMs from WT mice post-HSV-1 infection. However, these ubiquitination levels were significantly reduced in *Dtx2*-deficient BMDMs and PMs, indicating that DTX2 was required for efficient K63-linked ubiquitination of STING in response to HSV-1 infection. In addition, we found that deletion of the RING-H2 domain of DTX2, which harbors its enzymatic activity [21], led to defective DTX2-mediated STING ubiquitination (Supplementary Fig. S3G). These findings collectively indicate that DTX2 catalyzes the K63-linked ubiquitin chains on STING.

### DTX2 drives STING translocation, as well as TBK1 recruitment and subsequent activation

**Fig. 4:**
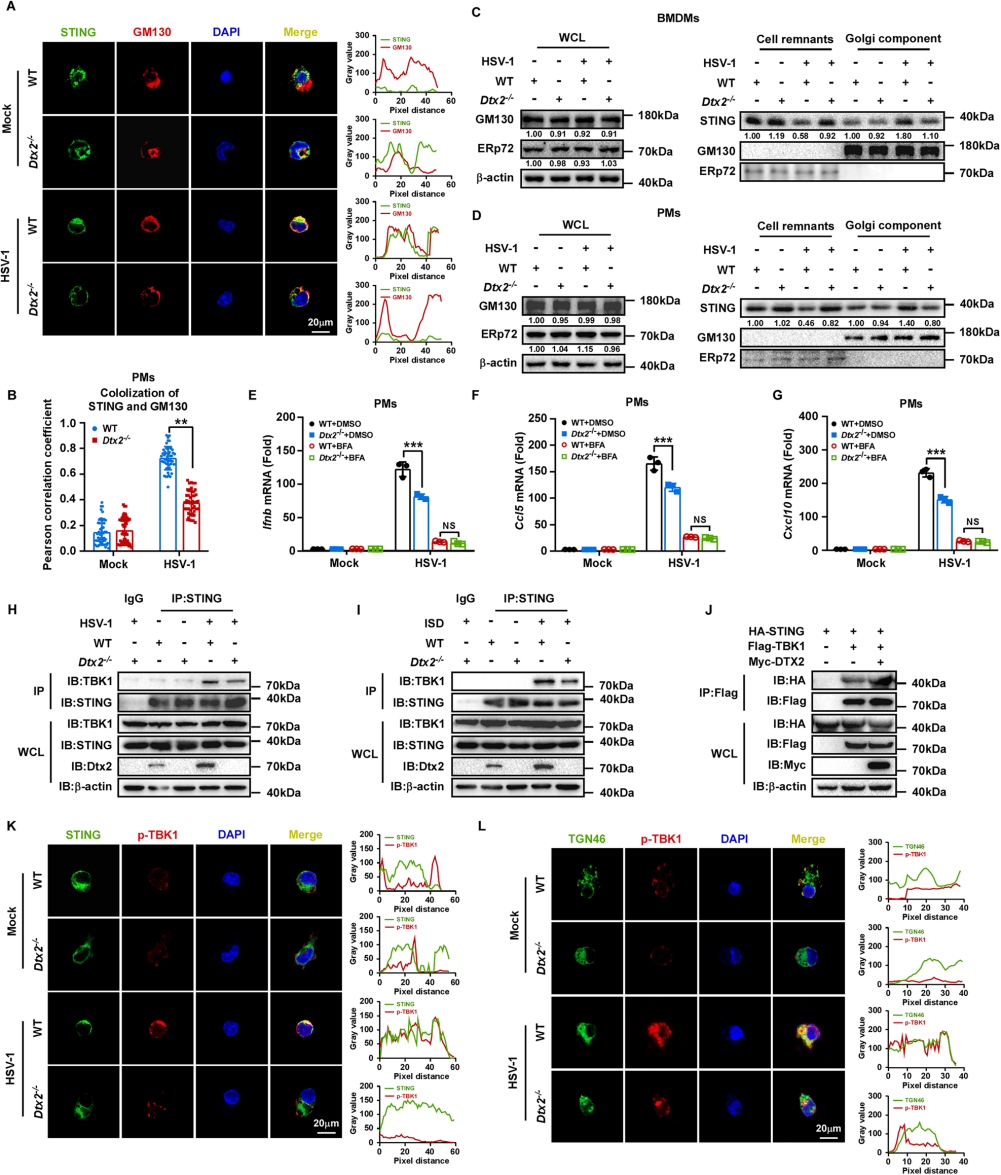
DTX2 facilitates STING translocation, as well as TBK1 recruitment and subsequent activation. **A** Fluorescence confocal images showing localization of STING (green) and GM130 (Golgi marker, red) in WT and *Dtx2*^−/−^ PMs. Scale bar = 20 µm. WT and *Dtx2*^−/−^ PMs were infected with HSV-1 for 4 h. Afterward, the cells were fixed and labeled with STING and GM130 antibodies. Nuclei were labeled with DAPI. **B** Co-localization of STING and GM130 in HSV-1-infected WT and Dtx2-deficient PMs was analyzed using ImageJ software, and Pearson correlation coefficients were statistically evaluated. *n* = 50. The Golgi apparatus was fractionated from WT and *Dtx2*^−/−^ BMDMs (**C**) or PMs (**D**) infected with HSV-1 for 4 h. WCL, cell remnants and Golgi apparatus-enriched components were immunoblotted for western blotting. **E**–**G** WT and *Dtx2*^−/−^ PMs were treated with BFA (5 μg/ml) or DMSO for 3 h, and then infected with HSV-1 for 4 h. Afterward, RT-PCR was used to measure the mRNA expression of *Ifnb*, *Ccl5*, and *Cxcl10*. Co-IP and western blotting analysis were performed to detect the interaction between STING and TBK1 in WT and *Dtx2*^−/−^ PMs infected with HSV-1 (**H**) or stimulated with ISD (**I**). **J** Co-IP and western blotting analysis were performed to detect the interaction between exogenous HA-STING and Flag-TBK1 in HEK293T cells transfected with or without Myc-DTX2. **K** Fluorescence confocal images showing localization of STING (green) and p-TBK1 (red) in WT and *Dtx2*^−/−^ PMs. Scale bar = 20 µm. WT and *Dtx2*^−/−^ PMs were infected with HSV-1 for 4 h. Afterward, the cells were fixed and labeled with STING and p-TBK1 antibodies. Nuclei were labeled with DAPI. Co-localization between STING and p-TBK1 was analyzed by ImageJ software. **L** Fluorescence confocal images showing localization of TGN46 (Golgi marker, green) and p-TBK1 (red) in WT and *Dtx2*^−/−^ PMs. Scale bar = 20 µm. WT and *Dtx2*^−/−^ PMs were infected with HSV-1 for 4 h. Afterward, the cells were fixed and labeled with TGN46 and p-TBK1 antibodies. Nuclei were labeled with DAPI. Co-localization between TGN46 and p-TBK1 was analyzed by ImageJ software. Data in (**E**–**G**) are shown as mean ± SD of three independent experiments. Statistical significance was analyzed by two-way ANOVA. **p < 0.01, ***p < 0.001, NS means no significance.

STING ubiquitination is indispensable for its stability, trafficking, and downstream signaling functionality [27]. Notably, as previously demonstrated, *Dtx2* deficiency does not impact overall STING protein levels, implying a regulatory role beyond protein expression. Since STING translocation from the ER to the Golgi apparatus is essential for signaling activation, we next investigated the role of DTX2 in this process. Employing confocal microscopy, we assessed the co-localization of STING with the Golgi apparatus. In *Dtx2*-deficient PMs and MEFs, we observed a significant reduction in STING-Golgi apparatus co-localization relative to WT controls following HSV-1 stimulation (Fig. 4A, B; Supplementary Fig. S4A, B). Furthermore, we isolated Golgi apparatus-enriched fractions from HSV-1-infected WT and *Dtx2*-deficient BMDMs, PMs, and MEFs. Western blotting analyses confirmed that STING translocation to the Golgi apparatus was significantly attenuated in *Dtx2*-deficient cells versus WT cells (Fig. 4C, D; Supplementary Fig. S4C), supporting the notion that DTX2 is necessary for dsDNA-stimulated STING trafficking. Additionally, treatment with Brefeldin A (BFA), which blocks STING trafficking, abolished the differential type I interferon response observed between WT and *Dtx2*-deficient cells following HSV-1 infection (Fig. 4E–G; Supplementary Fig. S4D–I), implying that DTX2’s effect on type I interferon production is dependent on STING translocation.

Considering the central role of STING-mediated TBK1 recruitment and activation in downstream signal transduction, we further investigated the influence of DTX2 on this crucial step. Co-IP analyses revealed that the interaction between TBK1 and STING was significantly diminished in *Dtx2*-deficient PMs following HSV-1 infection (Fig. 4H) or ISD stimulation (Fig. 4I), relative to WT controls. Conversely, overexpression of DTX2 in HEK293T cells augmented this interaction (Fig. 4J). To elucidate whether DTX2 influences STING-mediated TBK1 activation, we performed confocal microscopy to assess the co-localization of p-TBK1 with STING and the Golgi apparatus, respectively. Post-HSV-1 infection, *Dtx2*-deficient PMs and MEFs exhibited a marked reduction in the co-localization of p-TBK1 with both STING and the Golgi apparatus, compared to WT cells (Fig. 4K, L; Supplementary Fig. S4J–O). Collectively, these data indicate that DTX2 facilitates STING- mediated TBK1 recruitment and activation.

### DTX2 facilitates STING ubiquitination at K236 and K370

**Fig. 5:**
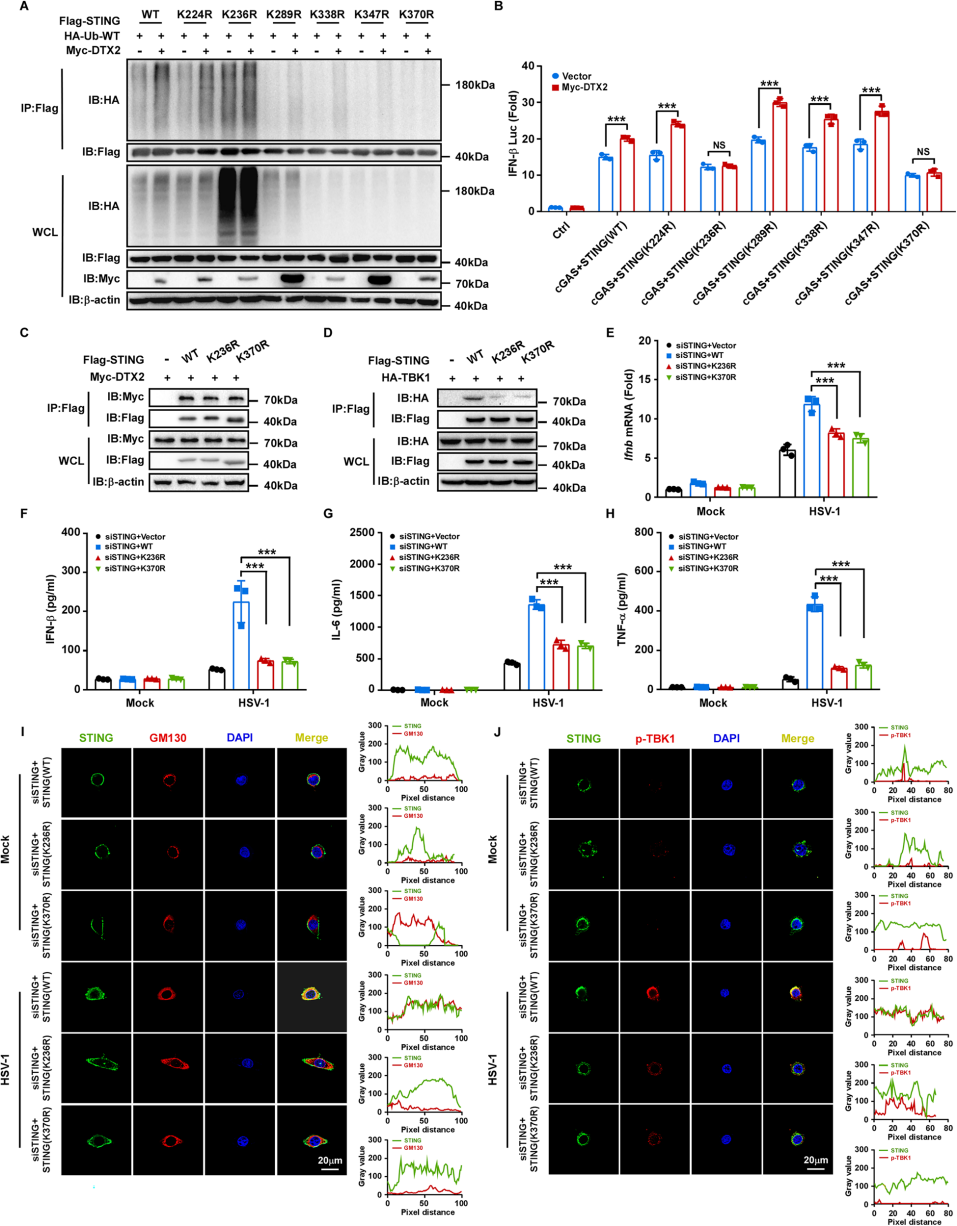
DTX2 facilitates STING ubiquitination at K236 and K370. **A** HEK293T cells were transfected with Myc-DTX2, HA-Ub-WT, Flag-STING-WT or mutants (K224R or K236R or K289R or K338R or K347R or K370R) for 24 h. The cell lysate was subjected to Co-IP and western blotting. **B** Luciferase activity in HEK293T cells transfected with IFN-β luciferase reporter, together with plasmids as indicated. **C** HEK293T cells were transfected with Myc-DTX2 and Flag-STING-WT or STING mutants (K236R or K370R) for 24 h. The cell lysate was subjected to Co-IP and western blot assays. **D** HEK293T cells were transfected with HA-TBK1 and Flag-STING-WT or STING mutants and incubated for 24 h. The cell lysate was subjected to Co-IP and western blot analysis. **E** After 24 h of transfection with Flag-STING-WT or its mutants, STING-knockdown HeLa cells were infected with HSV-1 for 4 h. RT-PCR was used to detect the expression level of *Ifnb* in each group. **F**–**H** STING-knockdown PMs were transfected with Flag-STING-WT or its mutants. Twenty-four hours later, cells were infected with HSV-1 for 36 h. IFN-β (**F**), IL-6 (**G**), and TNF-α (**H**) production was then quantified in each group using ELISA assay. **I**, **J** STING-knockdown HeLa were transfected with Flag-STING-WT or its mutants for 24 h, then infected with HSV-1 for 4 h. Afterward, the cells were fixed and labeled with the indicated antibodies. Co-localization analysis between STING and GM130, STING and p-TBK1 was conducted using ImageJ software. Representative images illustrating the co-localization of STING and GM130 (**I**), STING and p-TBK1 (**J**) in different groups. Scale bar = 20 μm. Data in (**B**, **E**–**H**) are shown as mean ± SD of three biological independent experiments. Statistical significance was analyzed by two-way ANOVA. ***p < 0.001, NS means no significance.

To determine whether DTX2-mediated ubiquitination of STING is essential for its role in the type I interferon signaling, we aimed to identify the specific ubiquitination sites on STING targeted by DTX2. Ubiquitination assays were conducted using WT STING and a series of mutants in which candidate lysine residues were substituted with arginine. Co-IP analysis revealed that the K236R and K370R mutants of STING exhibited resistance to DTX2-driven ubiquitination (Fig. 5A). Correspondingly, these two mutants did not support DTX2-induced upregulation of IFN-β promoter luciferase activity (Fig. 5B). These findings suggest that K236 and K370 are the principal ubiquitination sites on STING targeted by DTX2. Besides, despite preserving their interaction with DTX2, the STING K236R and K370R mutants impaired the recruitment of TBK1 (Supplementary Fig. S5A; Fig. 5C, D). To further explore the impact of DTX2-mediated STING ubiquitination on signaling activation, we knocked down endogenous STING and reintroduced either WT STING or these two mutants. The STING knockdown efficiency was verified (Supplementary Fig. S5B–E). Upon HSV-1 infection, the K236R and K370R mutants failed to evoke the type I interferon response and the production of inflammatory factors (Fig. 5E–H). Moreover, confocal microscopy confirmed that the co-localization of STING mutants (K236R and K370R) with the Golgi apparatus and p-TBK1 was diminished compared to WT STING following HSV-1 treatment (Fig. 5I, J; Supplementary Fig. S5F, G). Collectively, these data indicate that DTX2-mediated ubiquitination of STING at K236 and K370 is essential for efficient STING signaling activation.

### *Dtx2*-deficient mice are susceptible to HSV-1 infection

**Fig. 6:**
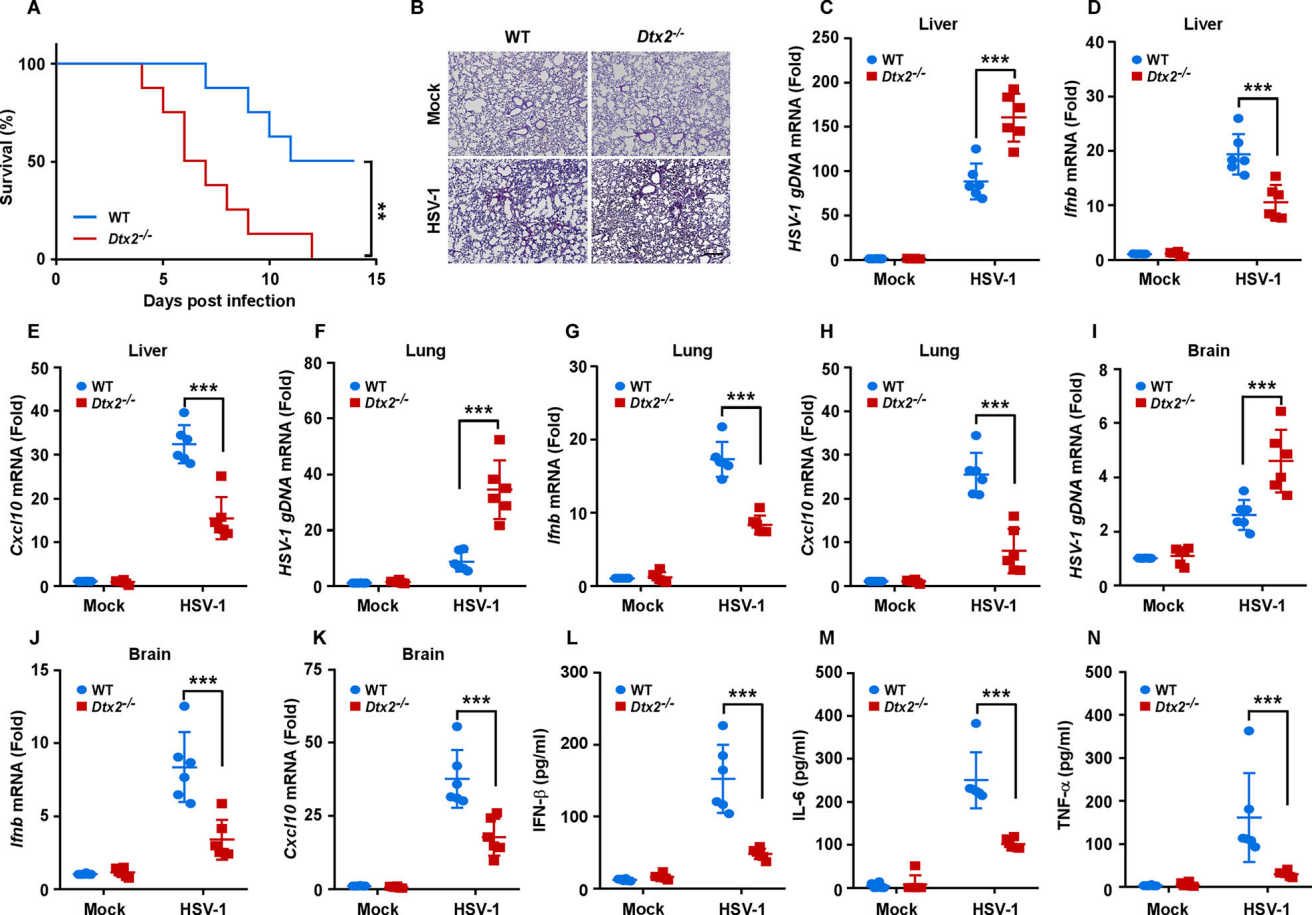
*Dtx2*-deficient mice are susceptible to HSV-1 infection. **A** The survival analysis of WT and *Dtx2*^−/−^ mice infected with HSV-1 through tail vein injection. All mice were monitored for 14 days (*n* = 8). **B** Sex- and age-matched WT and *Dtx2*^−/−^ mice were intravenously infected with HSV-1 for 12 h and lung sections were analyzed by H&E staining. Scale bar = 100 μm. C-K) WT and *Dtx2*^−/−^ mice were intravenously infected with HSV-1 for 12 h and then the liver (**C**–**E**), lung (**F**–**H**), and brain (**I**–**K**) of the mice were subjected to measure the mRNA expression of *HSV-1 gDNA*, *Ifnb*, and *Cxcl10* by RT-PCR assay. Levels of IFN-β (**L**), IL-6 (**M**), and TNF-α (**N**) in the serum of WT and *Dtx2*^−/−^ mice (*n* = 6) were measured by ELISA assay after intravenous infection with HSV-1 for 12 h. Data in (**C**–**N**) are shown as mean ± SD. **p < 0.01, ***p < 0.001.

To further assess the in vivo impact of DTX2 on HSV-1 infection, we infected WT and *Dtx2*^−/−^ mice with the HSV-1 virus. The *Dtx2*-deficient mice exhibited reduced overall survival (Fig. 6A) and more severe lung damage (Fig. 6B) compared to their WT counterparts. Analysis of viral titers, along with cytokine expression, across a range of organs revealed elevated HSV-1 replication, alongside diminished expression of *Ifnb* and *Cxcl10*, in the liver (Fig. 6C–E), lung (Fig. 6F–H), and brain (Fig. 6I–K) of *Dtx2*^−/−^ mice compared to WT mice. Furthermore, ELISA results showed significantly lower serum levels of IFN-β (Fig. 6L), IL-6 (Fig. 6M), and TNF-α (Fig. 6N) in *Dtx2*^−/−^ mice following intravenous HSV-1 infection. Collectively, these data underscore the protective role of DTX2 against HSV-1 infection in mice by fostering type I interferon and proinflammatory cytokine induction.

### DTX2 facilitates STING-mediated type I interferon response in multiple tumor types

**Fig. 7:**
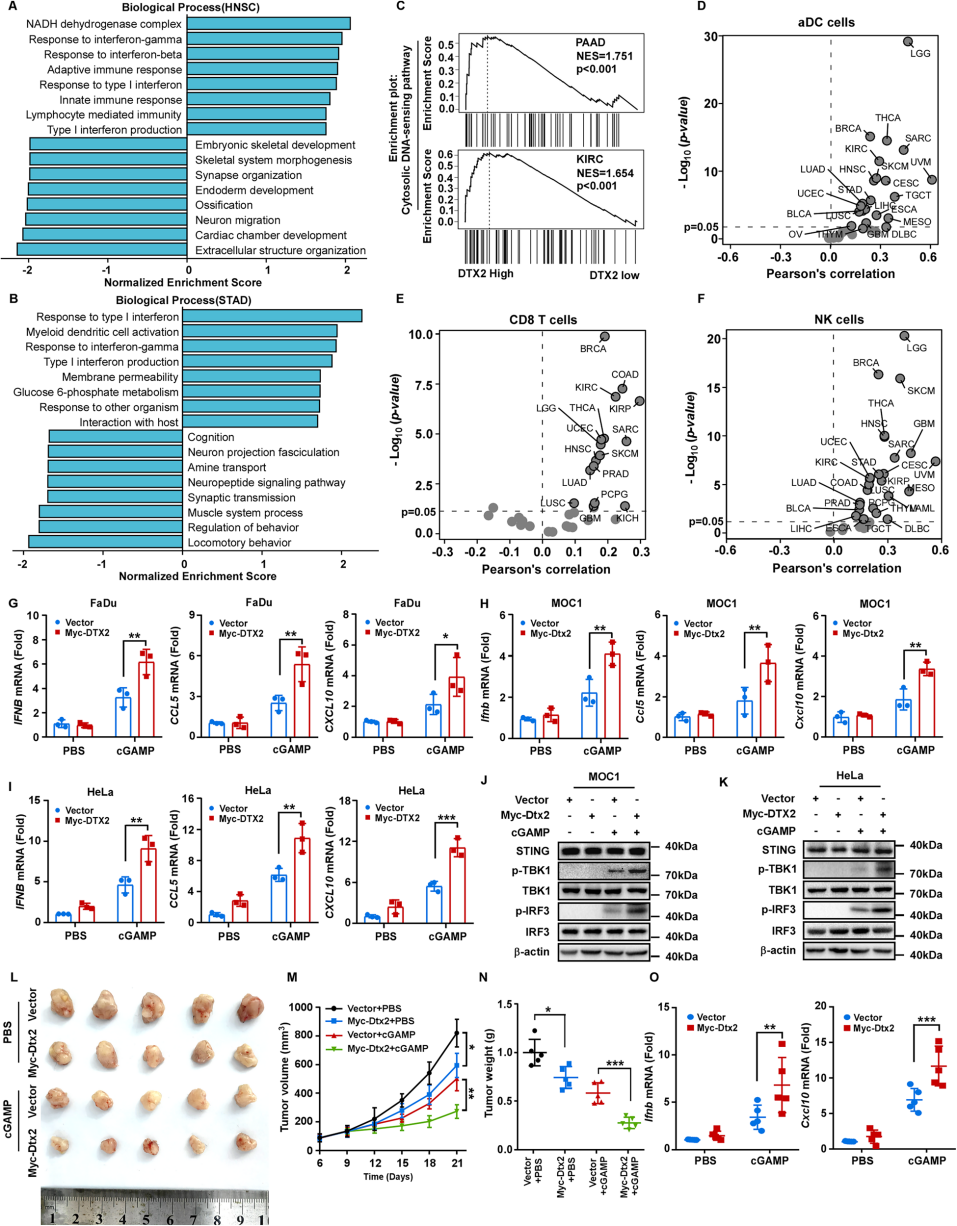
DTX2 facilitates STING-mediated type I interferon response in multiple tumor types. **A**, **B** The biological processes associated with DTX2 were analyzed in head and neck squamous cell carcinoma (HNSC) and stomach adenocarcinoma (STAD) using GSEA, and the results are summarized. **C** KEGG pathway analysis revealed significant enrichment of the cytosolic DNA-sensing pathway in the DTX2-high group in pancreatic adenocarcinoma (PAAD) and kidney renal clear cell carcinoma (KIRC). Correlation between DTX2 expression and the levels of infiltrating aDC cells (**D**), CD8^+^T cells (**E**), and NK cells (**F**) across various types of cancers based on TCGA data. BLCA bladder urothelial carcinoma, BRCA breast invasive carcinoma, CESC cervical squamous cell carcinoma and endocervical adenocarcinoma, COAD colon adenocarcinoma, DLBC lymphoid neoplasm diffuse large b-cell Lymphoma, ESCA esophageal carcinoma, GBM glioblastoma multiforme, KICH kidney chromophobe, KIRP kidney renal papillary cell carcinoma, LAML acute myeloid leukemia, LGG brain Lower grade glioma, LIHC liver hepatocellular carcinoma, LUAD lung adenocarcinoma, LUSC lung squamous cell carcinoma, MESO mesothelioma, OV ovarian serous cystadenocarcinoma, PCPG pheochromocytoma and paraganglioma, PRAD prostate adenocarcinoma, SARC sarcoma, SKCM skin cutaneous melanoma, TGCT testicular germ cell tumors, THCA thyroid carcinoma, THYM thymoma, UCEC uterine corpus endometrial carcinoma, UVM uveal melanoma. RT-PCR was used to quantify the mRNA expression of *IFNB, CCL5* and *CXCL10* in FaDu (**G**), MOC1 (**H**), and HeLa (**I**) cells with DTX2 overexpression following treatment with cGMAP. **J**, **K** MOC1 and HeLa cells were stimulated with cGAMP. Cells were collected and lysed for Western blotting. **L** Representative images of tumor growth in the labeled groups. **M** The growth curve of the tumor volumes measured on the indicated days. **N** Quantification data of tumor weights. **O** The expressions of *Ifnb* and *Cxcl10* mRNA in each group of tumor tissues were quantified by RT-PCR. Data in (**G**–**I**) are shown as mean ± SD of three biological independent experiments. *p < 0.05, **p < 0.01, ***p < 0.001.

Since STING signaling is crucial in anti-tumor immunity, our subsequent investigation focuses on whether DTX2 regulates STING signaling in tumors and participates the anti-tumor immune response. Leveraging TCGA data and GSEA, we identified a positive correlation between DTX2 expression and both the type I interferon response (Fig. 7A, B; Supplementary Fig. S6A–D) and the cytosolic DNA-sensing pathway (Fig. 7C; Supplementary Fig. S6E, F) across multiple cancer types. Given that STING activation enhances anti-tumor immunity by recruiting and activating immune cells such as DCs, CD8^+^ T cells, and NK cells [28, 29], we conducted immune infiltration analyses, which revealed a parallel positive correlation between DTX2 expression and infiltration levels of activated DCs (aDCs) (Fig. 7D), CD8^+^ T cells (Fig. 7E), and NK cells (Fig. 7F) in various tumor types. Moreover, high DTX2 expression, as assessed in prognostic analysis, proved predictive of improved survival in cancer patients receiving PD-1, PD-L1, or CTLA4 blockade immunotherapy (Supplementary Fig. S7A–C). These findings implicate DTX2 may facilitate STING-mediated type I interferon response in multiple tumor types. To further verify this, we transfected DTX2 expression plasmids into head and neck cell lines FaDu and MOC1, as well as the cervical cancer cell line HeLa (Supplementary Fig. S8A–C). Upon cGAMP stimulation, DTX2 significantly promoted the expression of *IFNB*, *CCL5* and *CXCL10* (Fig. 7G–I). Consistent results were observed when these cells were treated with cisplatin (Supplementary Fig. S8D–F), a first-line chemotherapy drug known to activate STING signaling. Subsequent western blot analyses revealed that tumor cells overexpressing DTX2 exhibited enhanced phosphorylation of TBK1 and IRF3 upon stimulation with either cGAMP or HSV-1 (Fig. 7J, K; Supplementary Fig. S8G). These observations support that DTX2 enhances STING-mediated type I interferon response in multiple tumor types.

As a STING agonist, cGAMP has been well established to elicit anti-tumor immunity via activation of the STING-type I interferon pathway [30]. Therefore, we sought to determine whether DTX2 could enhance this effect. We established a tumor model using MOC1 cells stably overexpressing Dtx2, followed by intratumoral administration of either PBS or cGAMP. Our results demonstrated that Dtx2 overexpression alone exerted a modest inhibitory effect on tumor growth and weight. However, the combination of Dtx2 overexpression with cGAMP treatment markedly potentiated tumor suppression, leading to significant reductions in both tumor volume and weight (Fig. 7L–N). Furthermore, transcriptional analysis revealed that the levels of *Ifnb*, *Cxcl10* and *Ccl5* were substantially elevated in tumors subjected to combined Dtx2 overexpression and cGAMP treatment (Fig. 7O; Supplementary Fig. S8H). These findings collectively confirm that DTX2 potentiates cGAMP-mediated anti-tumor immune response.

### DTX2 potentiates anti-tumor immunity

**Fig. 8:**
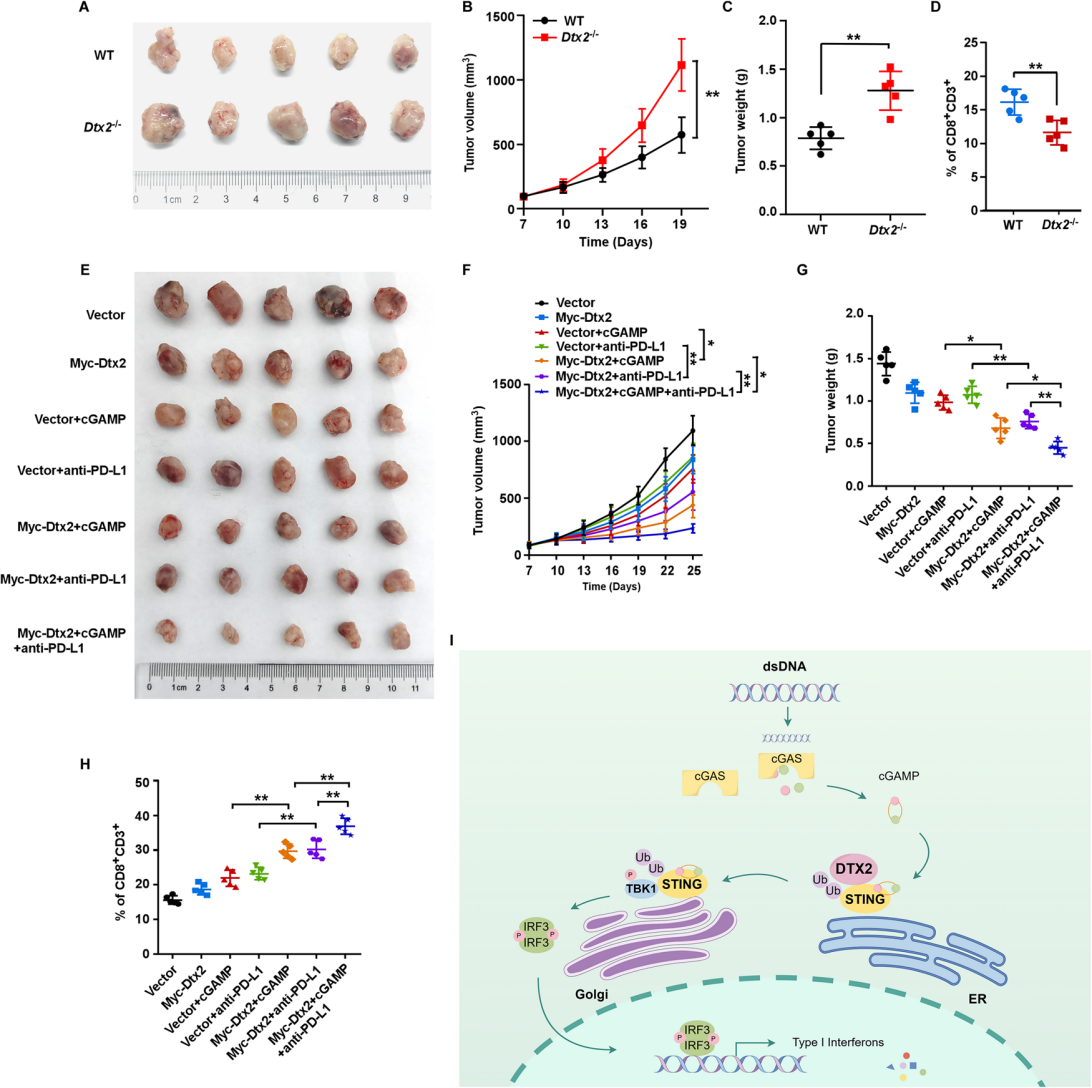
DTX2 potentiates anti-tumor immunity. **A** Representative images of tumor growth in the labeled groups. **B** The growth curve of the tumor volumes measured on the indicated days. **C** Quantification data of tumor weights. **D** The percentage of CD8^+^CD3^+^ T cells in tumor tissues. **E** Representative images of tumor growth in the labeled groups. **F** The growth curve of the tumor volumes measured on the indicated days. **G** Quantification data of tumor weights. **H** The percentage of CD8^+^CD3^+^ cells in tumor tissues. **I** The diagram depicts the mechanistic role of DTX2-mediated ubiquitination in amplifying STING-induced type I interferon production. *p < 0.05, **p < 0.01.

To elucidate the role of DTX2 in anti-tumor immunity, we established tumor models using MOC1 cells in both WT and *Dtx2*^−/−^ mice. Our findings revealed that tumors in *Dtx2*^−/−^ mice exhibited significantly accelerated growth and increased tumor weight (Fig. 8A–C). This was accompanied by a substantial reduction in CD8⁺ T cell and NK cell infiltration within the tumor microenvironment (Fig. 8D; Supplementary Fig. S9A; B). To further investigate DTX2’s impact on therapeutic response, we generated tumor models with MOC1 cells stably overexpressing Dtx2 and control counterparts. These models were subsequently treated with cGAMP, anti-PD-L1 antibody, or a combination of both. The results demonstrated that Dtx2 overexpression significantly sensitized the therapeutic efficacy of cGAMP or anti-PD-L1 antibody treatment. Notably, superior therapeutic outcomes were achieved when Dtx2 overexpression was combined with treatment using both cGAMP and the anti-PD-L1 antibody (Fig. 8E–G). Furthermore, immune infiltration analyses indicated that Dtx2 facilitated cGAMP- or anti-PD-L1 antibody-induced CD8^+^ T cell recruitment (Fig. 8H; Supplementary Fig. S9C). Additionally, Dtx2 also markedly boosted cGAMP-induced NK cell infiltration (Supplementary Fig. S9D). Collectively, these data powerfully implicates DTX2 in the potentiation of anti-tumor immunity.

## Discussion

As a fundamental component of the innate immune system, STING signaling is essential for initiating antiviral responses and maintaining effective tumor immune surveillance [31]. The activation of the STING pathway is governed by complex and tightly regulated mechanisms; notably, its translocation from the ER to the Golgi apparatus represents a critical step for subsequent downstream signaling and the induction of type I interferon response [32]. However, the dynamic regulatory mechanism of STING activation has not yet been fully elucidated. In this study, we elucidated the previously uncharacterized yet crucial role of the E3 ubiquitin ligase DTX2 in regulating the STING signaling. Our investigations revealed that BMDMs, PMs, and MEFs from *Dtx2* knockout mice exhibited significantly diminished type I interferon response and attenuated activation of STING-downstream signaling upon infection with HSV-1, or stimulation with cGAMP, and ISD, respectively. Consistent with these in vitro findings, *Dtx2* knockout mice demonstrated heightened susceptibility to infection with the DNA virus HSV-1 compared to their WT counterparts. Further mechanistic exploration via protein interaction analysis demonstrated that DTX2 forms a stable complex with STING. Crucially, DTX2-mediated ubiquitination promoted STING trafficking rather than protein degradation, thereby facilitating activation of downstream signaling cascades and type I interferon response.

STING, a transmembrane protein localized to the endoplasmic reticulum, undergoes a pivotal transition upon engagement with cGAMP, a cyclic dinucleotide synthesized by the upstream cytosolic dsDNA sensor cGAS. STING trafficking to the Golgi apparatus is necessary for initiating downstream signals. At the Golgi, STING recruits and activates TBK1, which leads to the phosphorylation of IRF3 and p65. Phosphorylated IRF3 and p65 subsequently translocate to the nucleus, inducing the expression of type I interferon and inflammatory factors [6]. Several proteins have been identified as essential mediators of the STING trafficking process, such as TMED2 [33], SNX8 [34], STEEP [35], and ACBD3 [36]. In this study, we demonstrated the essential role of DTX2 in STING trafficking and subsequent signaling cascade activation. Following dsDNA stimulation, *Dtx2* deficiency suppressed STING translocation to the Golgi apparatus in macrophages and MEFs, concurrently weakening the STING-TBK1 interaction; DTX2 overexpression, conversely, enhanced this interaction. Moreover, loss of *Dtx2* attenuated downstream STING signaling, as indicated by reduced phosphorylation of TBK1, IRF3, and p65, and decreased nuclear accumulation of IRF3 and p65. These findings collectively establish DTX2 as a crucial mediator for STING trafficking and the robust activation of its downstream signaling pathway.

Ubiquitination represents as a critical regulatory mechanism within the STING-type I interferon signaling pathway, wherein distinct ubiquitin chain types orchestrate precise modulation of STING signal transduction. K48-linked ubiquitination is well-characterized in mediating STING protein degradation [37–39]. In contrast, K63-linked ubiquitination is intimately linked to the activation of the STING signal [40–42]. Besides, emerging evidence suggests that other types of ubiquitination-including K6-, K27-, and K29-linked ubiquitination may also exert regulatory influence over STING activation [15, 16, 43]. In this study, through screening different types of ubiquitin chains, we demonstrated that DTX2 specifically promoted K63-linked ubiquitination of STING, which facilitated its trafficking from the ER to the Golgi and subsequently enhanced the activation of downstream signaling cascades. Notably, we identified two DTX2-modified specific STING ubiquitination sites, K236 and K370, demonstrating that any mutation at these sites would significantly impair STING trafficking and type I interferon response. Our findings, therefore, provide novel insights into the intricate mechanisms governing STING activation.

Beyond bolstering antiviral immunity, STING activation also potently promotes anti-tumor responses. STING agonists have demonstrated considerable anti-tumor efficacy across diverse cancer models, including ovarian cancer [44], colorectal cancer [45], and hepatocellular carcinoma [46]. Nevertheless, tumor cells frequently deploy diverse mechanisms to inhibit STING signaling, thereby circumventing immune surveillance and promoting immune evasion [47]. Addressing this challenge necessitates the identification of novel targets capable of amplifying STING signaling to enhance anti-tumor immunity. Building upon our foundational discovery that DTX2 enhances STING-interferon signaling, we extended our investigation to evaluate the potential role of DTX2 in regulating STING signaling in tumors and participating in anti-tumor immune responses. Our results indicated that DTX2 facilitated type I interferon response in multiple tumor cell lines and enhanced anti-tumor immunity in murine head and neck cancer models. The analysis of TCGA data corroborated these findings, demonstrating that DTX2 expression correlated positively with type I interferon response signatures and immune cell infiltration of various tumor types. Notably, patients with high DTX2 expression exhibited improved outcomes following immunotherapy. Overall, these findings position DTX2 as a promising target or biomarker for anti-tumor immunotherapy.

Our study demonstrates that DTX2 is upregulated during viral infection and plays a critical role in regulating STING-mediated antiviral and anti-tumor immunity. However, the upstream signaling pathways and transcriptional regulators responsible for this induction remain unclear. Intriguingly, DTX2 has been identified as a transcriptional target of phosphorylated STAT3 in lenvatinib-resistant hepatocellular carcinoma cells [26]. It will be a priority for future studies to determine whether STAT3 or other infection-responsive pathways (e.g., IRF3 or NF-κB signaling) are responsible for DTX2 induction during viral infection. Elucidating the precise mechanism of DTX2 induction represents a critical next step for the future research to fully understand its role in antiviral and anti-tumor immunity, which will provide valuable insights for developing novel therapeutic strategies.

In summary, our study elucidates a critical regulatory mechanism by which the E3 ubiquitin ligase DTX2 amplifies STING-mediated type I interferon signaling. We demonstrate that DTX2 catalyzes K63-linked ubiquitination of STING at lysine residue 236 and 370. This specific modification is essential for its proper intracellular trafficking of STING, which in turn enables the robust activation of downstream signaling kinases, culminating in potentiated type I interferon production (Fig. 8I). Our results not only deepen our understanding of the intricate post-translation control of the STING-pathway but also identify DTX2 as a promising therapeutic target for modulating innate immunity in the context of viral infections and tumors.

## Methods

### Mice

*Dtx2*^−/−^ mice were purchased from Shanghai Model Organisms (Shanghai, China). All mice used in this study were on the C57BL/6J background and housed in a specific pathogen-free (SPF) facility with free access to food and water. Animal experiments were performed in accordance with the guidelines approved by the Ethics Committee of Tianjin Medical University (TMUaMEC2023044).

### Cell culture

BMDMs, PMs and MEFs were obtained as previously described [48, 49]. BMDMs and PMs cultured in RPMI-1640 medium, whereas MEFs were cultured in Dulbecco’s modified Eagle’s medium (DMEM) medium. HEK293T cells and HeLa cells were obtained from the American Type Culture Collection (ATCC, Manassas, VA, USA) and cultured in DMEM medium. The human pharyngeal carcinoma cell FaDu (from ATCC) and mouse oral cancer cell MOC1 (from Meisen CTCC) were cultured in DMEM-F12 medium. All cells were supplemented with 10% fetal bovine serum (FBS) and 1% penicillin/streptomycin (P/S) and cultured in a cell culture incubator at 37 °C with 5% CO_2_.

### Viruses and infection

BMDMs, PMs, and MEFs were infected with HSV-1 (10 MOI) during a designated time period. After infection, cells were washed thoroughly with PBS and collected for subsequent assays. For in vivo infection studies, HSV-1 (1 × 10^7^ PFU per mouse) was delivered via tail vein injection to age-, weight-, and sex-matched WT and *Dtx2*^−/−^ mice.

### Plasmids and transfection

Plasmids encoding murine Myc-Dtx2, human Myc-DTX2, and their domain-deletion mutants (ΔWWE, Δproline-rich, ΔRING-H2, and ΔDTC), along with Flag-STING and its domain-deletion mutants (ΔC and ΔN), were obtained from Miaoling Biotechnology (Wuhan, China). Ubiquitin and its mutants (HA-Ub-WT, HA-Ub-K6, HA-Ub-K11, HA-Ub-K27, HA-Ub-K29, HA-Ub-K33, HA-Ub-K48, and HA-Ub-K63) were generously provided by Dr. Xi Wang at Ditan Hospital, Capital Medical University. All constructs were validated via DNA sequencing. Plasmid transfections into HEK293T cells were carried out using Lipofectamine 2000 following the manufacturer’s instructions.

### Antibodies and reagents

The following antibodies were used: Anti-p-TBK1/NAK (Ser172) (5483S) was purchased from Cell Signaling Technology. Anti-STING (A3575), Anti-p-p65 (AP1294), Anti-p65 (A2547), Anti-p-TBK1 (AP1026), Anti-TBK1 (A3458), Anti-p-IRF3-S396 (AP0623), Anti-IRF3 (A2172), Anti-Myc (AE070), Anti-HA (AE105), and Anti-GM130 (A16248) were purchased from Abclonal. Anti-Flag (TAG10001) was purchased from Bioswamp. Anti-STING (19851-1-AP) and Anti-TGN46 (13573-1-AP) were purchased from Proteintech. Anti-β-actin (T0022), Goat anti-rabbit (S0001), and Goat anti-mouse (S0002) were purchased from Affinity Biosciences. Anti-CD45 (557659), anti-CD3 (562600) and anti-CD8 (552877) were purchased from BD Biosciences. Anti-NK1.1 (156506) was purchased from biolegend. FITC-Conjugated Goat Anti-Rabbit IgG (H + L) and Rhodamine (TRITC)-Conjugated Goat anti-Mouse IgG (H + L) were purchased from ZSGB-BIO.

2’3’-cGAMP (tlrl-nacga23-02) was purchased from InvivoGen. Protein A/G agarose used for immunoprecipitation was purchased from Thermo Fisher Scientific. Mouse IFN-β (E-EL-M0033), IL-6 (E-EL-M0044), and TNF-α (E-EL-M3063) ELISA Kits were purchased from Elabscience. Nuclear and cytoplasmic protein extraction kit was purchased from Glpbio. Golgi apparatus enrichment kit was purchased from Invent Biotechnologies. Triple-color multiplex fluorescent immunohistochemistry IHC kit (abs50088) was purchased from Absin.

### RNA sequencing (RNA-seq) analysis

After BMDMs was exposed to HSV-1 or control treatment for 4 h, total RNA was extracted with TRIzol reagent. Library construction and sequencing services were provided by OBiO Technology (Shanghai, China). Genes with a *p*-value < 0.05 and |log_2_FC|> 1 were considered differentially expressed.

### Quantitative real-time PCR (RT-PCR)

This assay was performed as described previously [50]. Briefly, total RNA was extracted using TRIzol reagent and reverse-transcribed into cDNA with a commercial kit (Vazyme, China) according to the manufacturer’s protocol. Quantitative real-time PCR was conducted with SYBR Green incorporation on Roche LightCycler480 Instrument II. Relative gene expression was normalized to *Gapdh* or *ACTIN* in each sample. The specific primer sequences for all RT-PCR assays are detailed in Supplementary Table S1.

### Co-IP and western blotting

For Co-IP experiments, cells were harvested and lysed in IP lysis buffer (20 mM Tris pH 7.5, 0.5% Triton X-100, 10% glycerol, 150 mM NaCl, 20 mM MgCl₂, 1 mM EDTA, 1 mM PMSF) at 4 °C for 30 min. Following centrifugation at 12,000 rpm for 15 minutes to clear cellular debris, the supernatants were collected and incubated overnight at 4 °C with anti-Flag magnetic beads, anti-Myc magnetic beads, or specific antibodies pre-conjugated to Protein A/G beads. After three washes with IP buffer, the immunoprecipitated complexes were eluted by boiling in 2× SDS loading buffer.

For subsequent western blotting, cells were lysed using RIPA buffer supplemented with a protease inhibitor cocktail. Total protein concentrations were determined using a bicinchoninic acid (BCA) protein assay kit (Thermo Fisher Scientific, USA). Equal amounts of protein extracts were separated by sodium dodecyl sulfate-polyacrylamide gel electrophoresis (SDS-PAGE) and then transferred onto polyvinylidene difluoride (PVDF) membranes for downstream antibody probing and detection.

### GST pull-down assay

Recombinant GST-DTX2 and His-STING proteins were individually expressed and purified from *Escherichia coli* BL21 using standard protocols. Purified GST-DTX2 was first immobilized onto glutathione-Sepharose beads. These GST-DTX2-bound beads were then incubated with purified His-STING for 3 h at 4°C. Following the incubation period, the beads were subjected to rigorous washing. Finally, the captured proteins complexes were subsequently analyzed by western blotting.

### Ubiquitination assay

To investigate the ubiquitination of STING, HEK293T cells were co-transfected with plasmids encoding Flag-STING, HA-Ub-WT or specific ubiquitin mutants (K6, K11, K27, K29, K33, K48, and K63), and Myc-DTX2. Proteins were subsequently immunoprecipitated from whole cell extracts using an anti-Flag antibody. The ubiquitination status of STING was then assessed by immunoblotting with an anti-HA antibody.

### ELISA

Eight-week-old WT and *Dtx2*^−/−^ mice were infected with HSV-1 for a duration of 12 hours. Subsequently, plasma samples were collected and the concentrations of IFN-β, IL-6, and TNF-α were quantified using commercially available ELISA kits, following the manufacturer’s protocols.

### luciferase reporter analysis

Luciferase reporter analysis was performed as described previously [51].

### RNA interference

STING small interfering RNA (siRNA) and a negative control siRNA were obtained from Sangon Biotech. Their sequences are listed below: siSTING-1: Forward, 5’-UGUUGCUGCUGUCCA UCUA-3’; Reverse, 5’-UAGAUGGACAGCAGCAACA-3’. siSTING-2: Forward, 5’-GGUCA UAUUACAUCGGAUA-3’; Reverse, 5’-UAUCCGAUGUAAUAUGACC-3’. siSTING-3: Forward, 5’-GCAUUACAACAACCUGCUA-3’; Reverse, 5’-UAGCAGGUUGUUGUAAUGC-3’. Transfection of these siRNAs into cells was performed using RNAmate (G04001, GenePharma) as per the manufacturer’s recommended protocol. Subsequent experiments were conducted with the transfected cells at the 48 h post-transfection time point.

### Confocal microscopy

Confocal microscopy was performed as described previously [52]. Briefly, post-treatment, cells underwent sequential fixation with 4% paraformaldehyde (PFA) in PBS, permeabilization with 0.5% Triton X-100, and blocking with 5% BSA in PBS. Multiplex fluorescence staining was then conducted as per the manufacturer’s instructions for the triple-color kit. Nuclear visualization was achieved using 4’,6-diamidino-2-phenylindole (DAPI). Image acquisition was performed on an Olympus 900 laser confocal ultra-high resolution microscope.

### Golgi apparatus isolation

BMDMs, PMs, and MEFs from WT and *Dtx2*^−/−^ mice were infected with HSV-1 or treated with PBS for 4 h. Golgi apparatus isolation was then performed based on the manufacturer’s recommended procedure. In brief, cells were collected, washed with PBS, and resuspended in Buffer A. After vortexing and centrifugation, the resulting cytosolic pellet was discarded. The supernatant was mixed with Buffer B and incubated on ice, followed by centrifugation. The pellet was resuspended in Buffer A, treated with Buffer C, and incubated again before recentrifugation to remove residual contaminants, discarding the supernatant. The enriched Golgi fraction was finally resuspended in an appropriate storage buffer for downstream applications.

### Nuclear and cytoplasmic protein extraction

WT and *Dtx2*^−/−^ BMDMs, PMs, and MEFs were infected with HSV-1 or treated with PBS for 4 h. Subcellular fractionation was performed to extract nuclear and cytoplasmic proteins following the manufacturer’s instructions. Briefly, cells were harvested and incubated with Reagent A, then mixed thoroughly. Reagent B was subsequently added to the homogenate, which was centrifuged to yield a supernatant containing cytoplasmic proteins. The pellet was resuspended in Reagent C to isolate nuclear proteins.

### Tumor models

Female C57BL/6J mice (6-8 weeks old) were randomly assigned to two groups (*n* = 10 per group) and subcutaneously injected with 1 × 10^6^ MOC1-Vector or MOC1-Myc-Dtx2 cells in 100 μL of PBS. Tumor length (L) and width (W) were measured with calipers, and tumor volume (V) was calculated every 3 days as 0.5 × L × W². When tumors reached ~100 mm³ in volume, mice within each group were randomly subdivided and administered intratumoral injections of either PBS or 10 μg cGAMP twice a week for 2 weeks. For immune checkpoint blockade (ICB) therapy, Mice received intraperitoneal injections of anti-PD-L1 antibody (200 μg per mouse; clone 10 F.9G2, Bio X Cell) every three days, starting on day 10. Upon sacrifice, tumors were excised, weighed, and stored for subsequent studies.

### Flow cytometry analysis

Tumors under various treatment conditions were collected and dissociated into single-cell suspensions. Then, the suspensions were incubated with the specified panel of fluorescently labeled surface antibodies for 30 min at 4 °C in the dark. Data collection was performed on an LSRFortessa flow cytometer (BD Biosciences), followed by analysis with FlowJo software (TreeStar). Gates and quadrants were rigorously set based on negative control staining.

### Bioinformatics analysis

In this study, RNA-seq data from 32 tumor types were obtained from the TCGA database (https://portal.gdc.cancer.gov) and organized to analyze the correlation between DTX2 expression and immune cell infiltration. Analyses were conducted using R software (version 4.2.1). The Gene Set Variation Analysis (GSVA) package (version 1.46.0) was employed to estimate immune cell abundance via the single-sample GSEA (ssGSEA) method [53], utilizing immune cell marker gene sets based on reference [54]. Data visualization was achieved with the ggplot2 package (version 3.4.4). Statistical associations were determined via Pearson’s correlation coefficient, with a threshold of *p* < 0.05 for significance.

Gene Set Enrichment Analysis (GSEA) tool within the LinkedOmics platform (http://www.linkedomics.org/login.php), a comprehensive multi-omics data resource integrating clinical and genomic data across 32 cancer types [55], was used to identify enriched GO_biological processes and KEGG pathways linked to DTX2. Significance was assigned at *p* < 0.05.

The prognostic value of DTX2 in patients treated with immune checkpoint inhibitors (PD-1, PD-L1, or CTLA4 blockade) was evaluated using the Kaplan–Meier Plotter (http://kmplot.com/analysis/) [56]. Hazard ratios (HR) with 95% confidence intervals (CI) and log-rank *p*-values were calculated.

### Statistical analysis

Statistical analyses were carried out with GraphPad Prism version 9.0. Data represent the mean ± SD from at least three independent experiments. Comparisons between two groups were conducted using Student’s *t* tests. For comparisons of three or more groups with a single independent variable, statistical significance was determined by two-way ANOVA. The Kaplan–Meier method was used to perform survival analysis. Differences were considered statistically significant at *p* < 0.05. *P*-values are denoted as: *p < 0.05, **p < 0.01, ***p < 0.001.

## Supplementary information

Supplementary materials

Supplementary Table

Western blots

## Data Availability

Data will be made available on request.
