# Supplementary material for: Deltex E3 ubiquitin ligase 2 potentiates STING-mediated type I interferon response by K63-linked ubiquitination
Source: Cell Death Dis. 2026 Mar 28;17(1):424. doi: 10.1038/s41419-026-08659-4 (PMC13150011; doi:10.1038/s41419-026-08659-4)
Supplement: Supplementary file 1 — Supplementary materials [file 41419_2026_8659_MOESM1_ESM.docx]

Supplementary Materials for

**Deltex E3 ubiquitin ligase 2 potentiates STING-mediated type I interferon response by K63-linked ubiquitination**

Zhuang Liu, Runze Li, *et al.*

*Corresponding author. E-mail: liuchang419@tmu.edu.cn (Chang Liu), shenyanna@tmu.edu.cn (Yanna Shen), qizhi@nankai.edu.cn (Zhi Qi), wxd.1133@163.com (Xudong Wang)

**This PDF file includes:**

Figs. S1 to S9


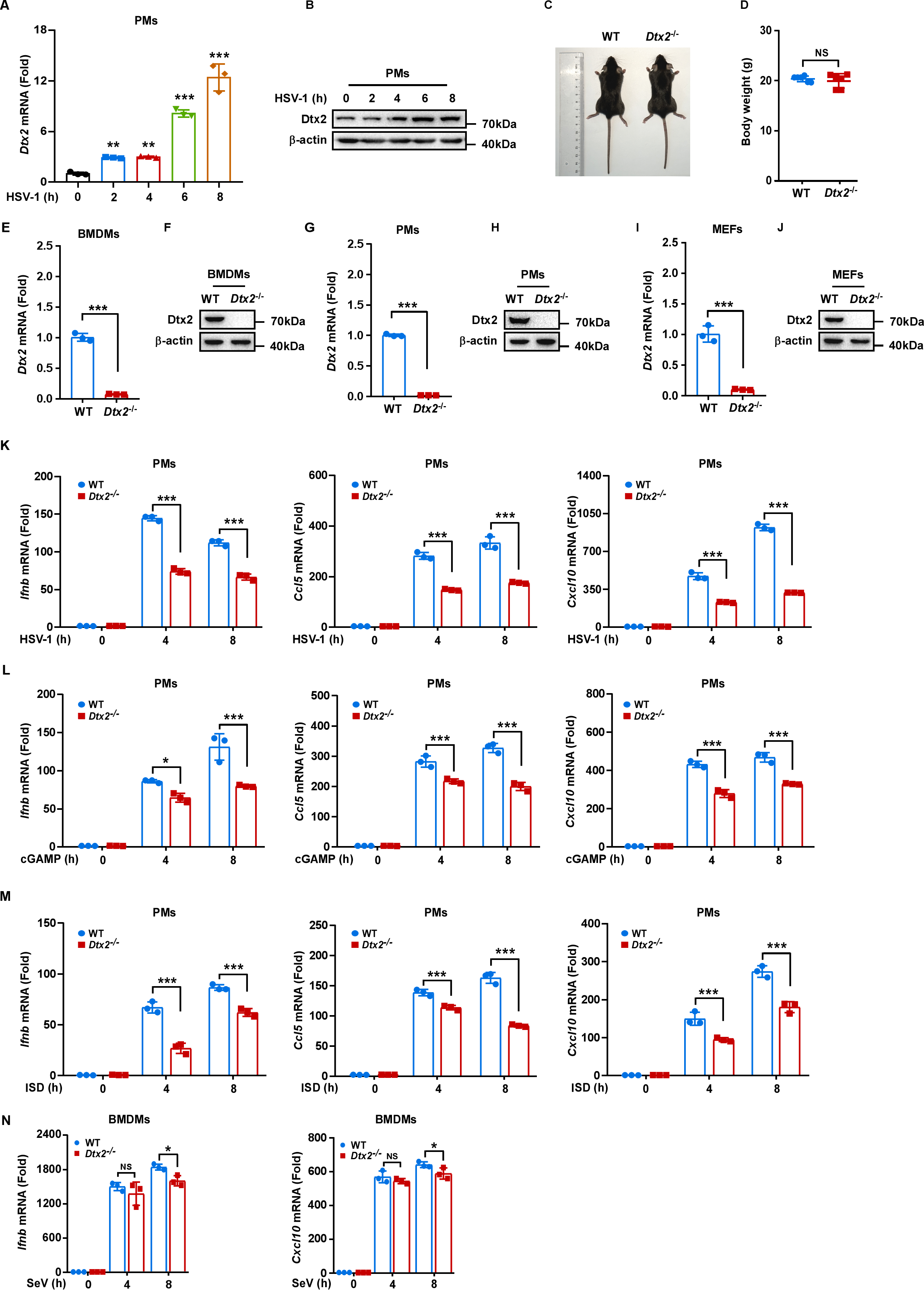


Figure S1. DTX2 positively regulates dsDNA-induced type I interferon response. A, B) Dtx2 expression in PMs infected with HSV-1 for the indicated time was detected by RT-PCR (A) and western blot assay (B). C) The image of sex- and age-matched WT and *Dtx2*^-/-^ mice. D) The body weight of sex- and age-matched WT and *Dtx2*^-/-^ mice (n = 6). E-J) Dtx2 expression in BMDMs (E, F), PMs (G, H), and MEFs (I, J) from WT and *Dtx2*^-/-^ mice was detected by RT-PCR assay and western blot assay. K-M) WT and *Dtx2*^-/-^ PMs were infected with HSV-1(K), or stimulated with cGAMP (L) and ISD (M) for the 0, 4, and 8 h. RT-PCR was used to measure the mRNA expression of *Ifnb*, *Ccl5*, and *Cxcl10*. N) WT and *Dtx2*^-/-^ BMDMs were infected with SeV for the 0, 4, and 8 h. RT-PCR was used to measure the mRNA expression of *Ifnb* and *Cxcl10*. Data in (D, E, G, I, K-N) are shown as mean ± SD of three independent experiments. **p* < 0.05, ***p* < 0.01, ****p* <0.001, NS means no significance.


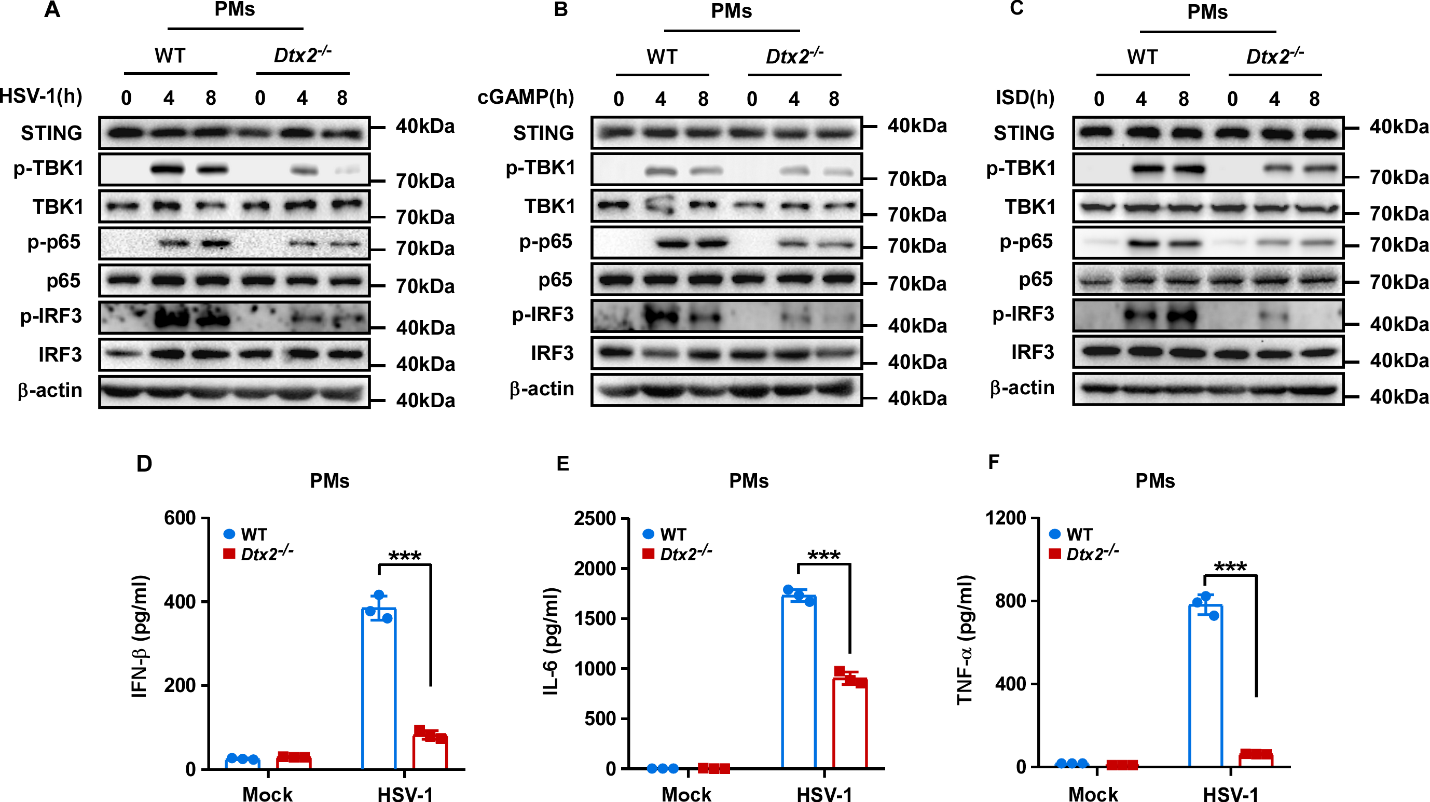


Figure S2. DTX2 is required for dsDNA-induced STING-type I interferon signaling. A-C) WT and *Dtx2*^-/-^ PMs were infected with HSV-1 (A), stimulated with cGAMP (B) or ISD (C) for 0, 4, and 8 h. Cells were collected and lysed for western blotting. D-F) WT and *Dtx2*^-/-^ PMs were infected with HSV-1 for 36 h. IFN-β (D), IL-6 (E), and TNF-α (F) production was then quantified in each group using ELISA assay. Data in (D-F) are shown as mean ± SD of three independent experiments. ****p* <0.001.


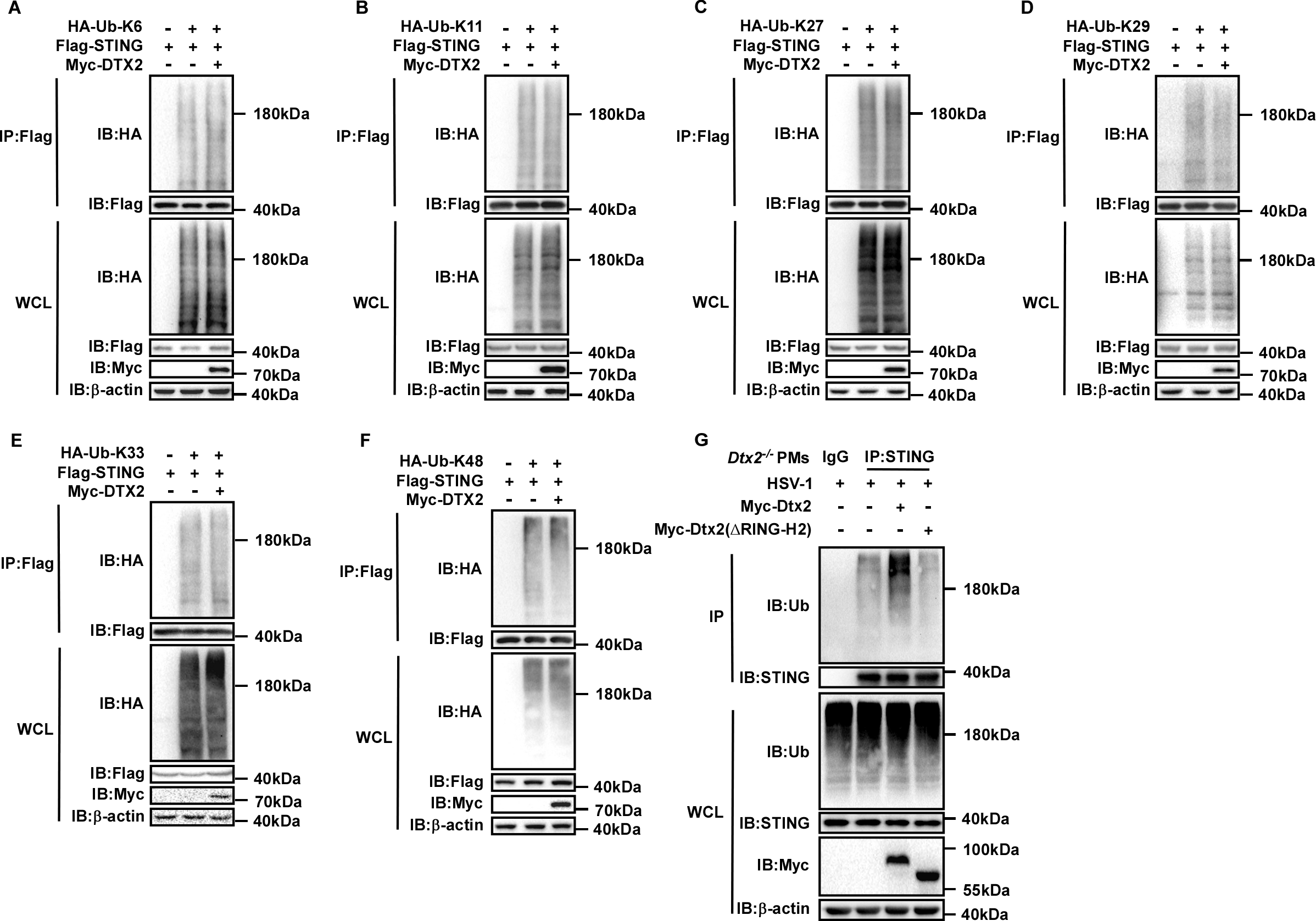


Figure S3. DTX2 promotes STING ubiquitination. A-F) HEK293T cells transfected with Myc-DTX2, Flag-STING, and HA-tagged K6 (A), K11 (B), K27 (C), K29 (D), K33 (E), and K48 (F) mutant plasmids for 24 h. Cell lysate was subjected to Co-IP using anti-Flag beads, followed by western blot analysis with anti-HA antibody. (G) *Dtx2*^-/-^ PMs transfected with Myc-Dtx2 and Myc-Dtx2(ΔRING-H2) mutant plasmids for 24 h. The endogenous ubiquitination of STING were measured by Co-IP assay with anti-STING antibody, followed by western blotting with indicated antibodies.


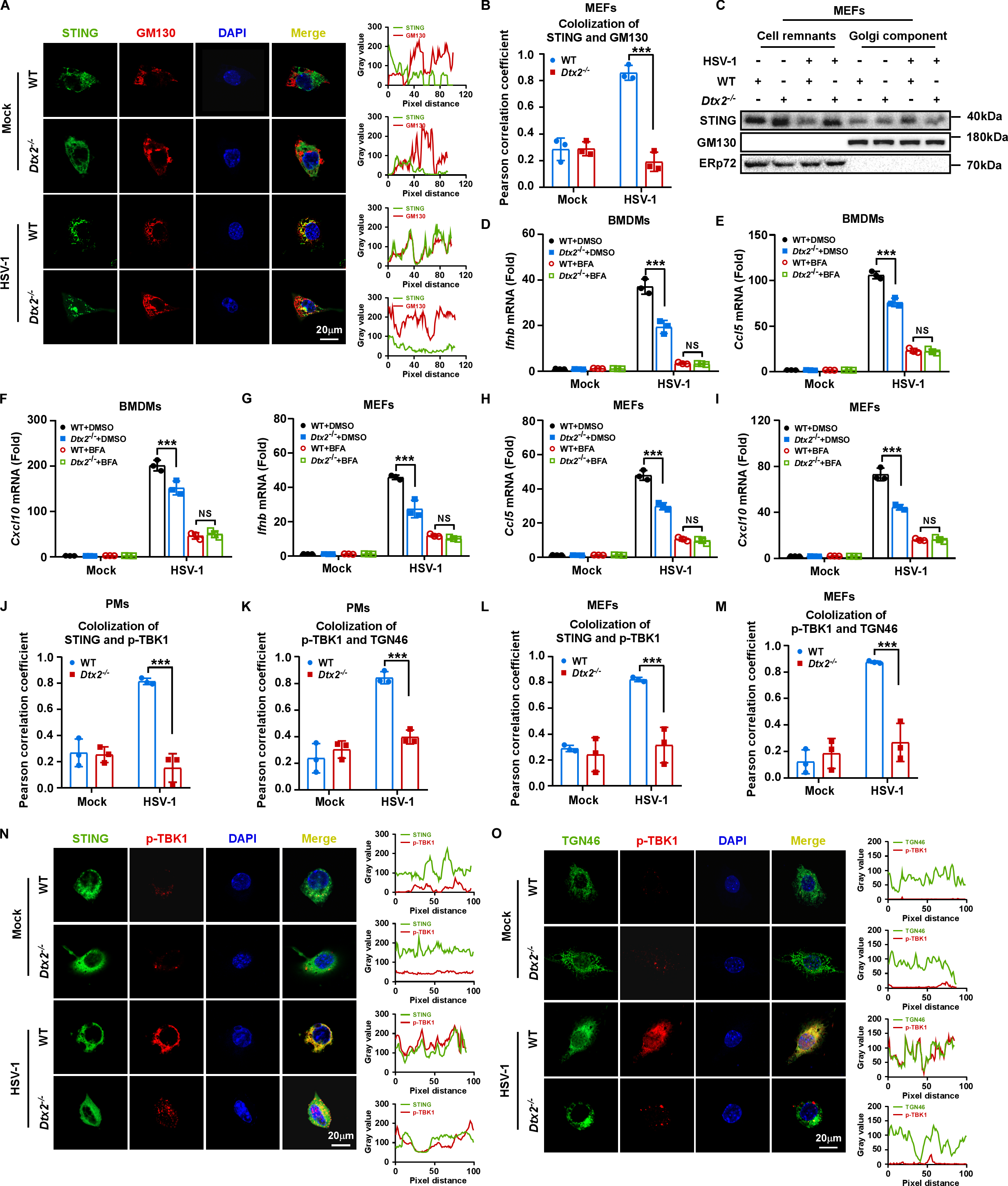


Figure S4. *Dtx2*-deficient impairs STING translocation. A, B) Representative images illustrating the co-localization of STING and GM130 in HSV-1-infected WT and *Dtx2*-deficient MEFs (A). Co-localization of STING and GM130 in HSV-1-infected WT and *Dtx2*-deficient MEFs was analyzed using ImageJ software, and Pearson correlation coefficients were statistically evaluated (B). Scale bar = 20 μm. C) The Golgi apparatus was fractionated from WT and *Dtx2*^-/-^ MEFs infected with HSV-1 for 4 h. Cell remnants and Golgi apparatus-enriched components were separately analyzed by western blotting. D-I) WT and *Dtx2*^-/-^ BMDMs and MEFs were treated with BFA (5 μg/ml) or DMSO for 3 h, followed by infection with HSV-1 for 4 h. Subsequently, mRNA expression levels of *Ifnb*, *Ccl5*, and *Cxcl10* were quantified by RT-PCR. (J, K) Pearson correlation coefficients for the co-localization of STING and p-TBK1 (J), and p-TBK1 and TGN46 (K) were statistically analyzed in HSV-1-infected WT and *Dtx2*-deficient PMs. L-O) WT and *Dtx2*^-/-^ MEFs were infected with HSV-1 for 4 h. Afterward, the cells were fixed and labeled with the indicated antibodies. Co-localization analysis between STING and p-TBK1, TGN46 and p-TBK1 were conducted using ImageJ software, and Pearson correlation coefficients were statistically evaluated (L, M). Representative images illustrating the co-localization of STING and p-TBK1 (N), TGN46 and p-TBK1 (O) in HSV-1-infected WT and *Dtx2*-deficient MEFs. Scale bar = 20 μm. Data in (B, D-M) are shown as mean ± SD of three independent experiments. ****p* < 0.001, NS means no significance.


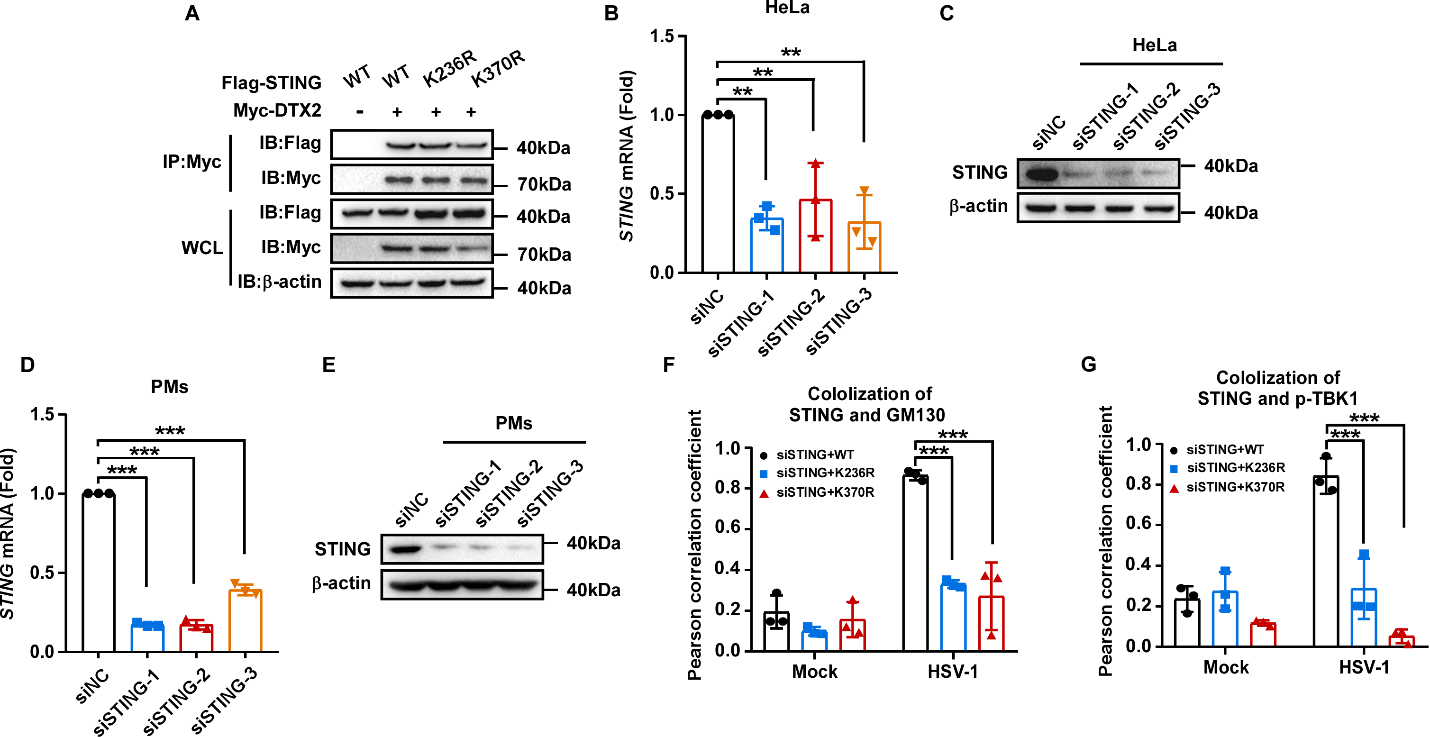


Figure S5. STING K236R and K370R mutations preserve interaction with DTX2 but impair STING translocation. A) HeLa cells were transfected with Myc-DTX2, Flag-STING-WT or mutants (K236R or K370R) for 24 h. The cell lysate was subjected to Co-IP and western blot assays. B, C) After transfection of HeLa cells with siSTING, STING expression was detected by RT-PCR (B) and western blot assay (C). D, E) After transfection of PMs with siSTING, STING expression was detected by RT-PCR (D) and western blot assay (E). (F, G) STING-knockdown HeLa were transfected with Flag-STING (WT) or mutants (K236R and K370R) for 24 h, then infected with HSV-1 for 4 h. Afterward, the cells were fixed and labeled with the indicated antibodies. Co-localization analysis between STING and GM130 (F), STING and p-TBK1 (G) were conducted using ImageJ software, and Pearson correlation coefficients were statistically evaluated. Data in (B, D, E, F, G) are shown as mean ± SD of three independent experiments. ***p* < 0.01, ****p* < 0.001.


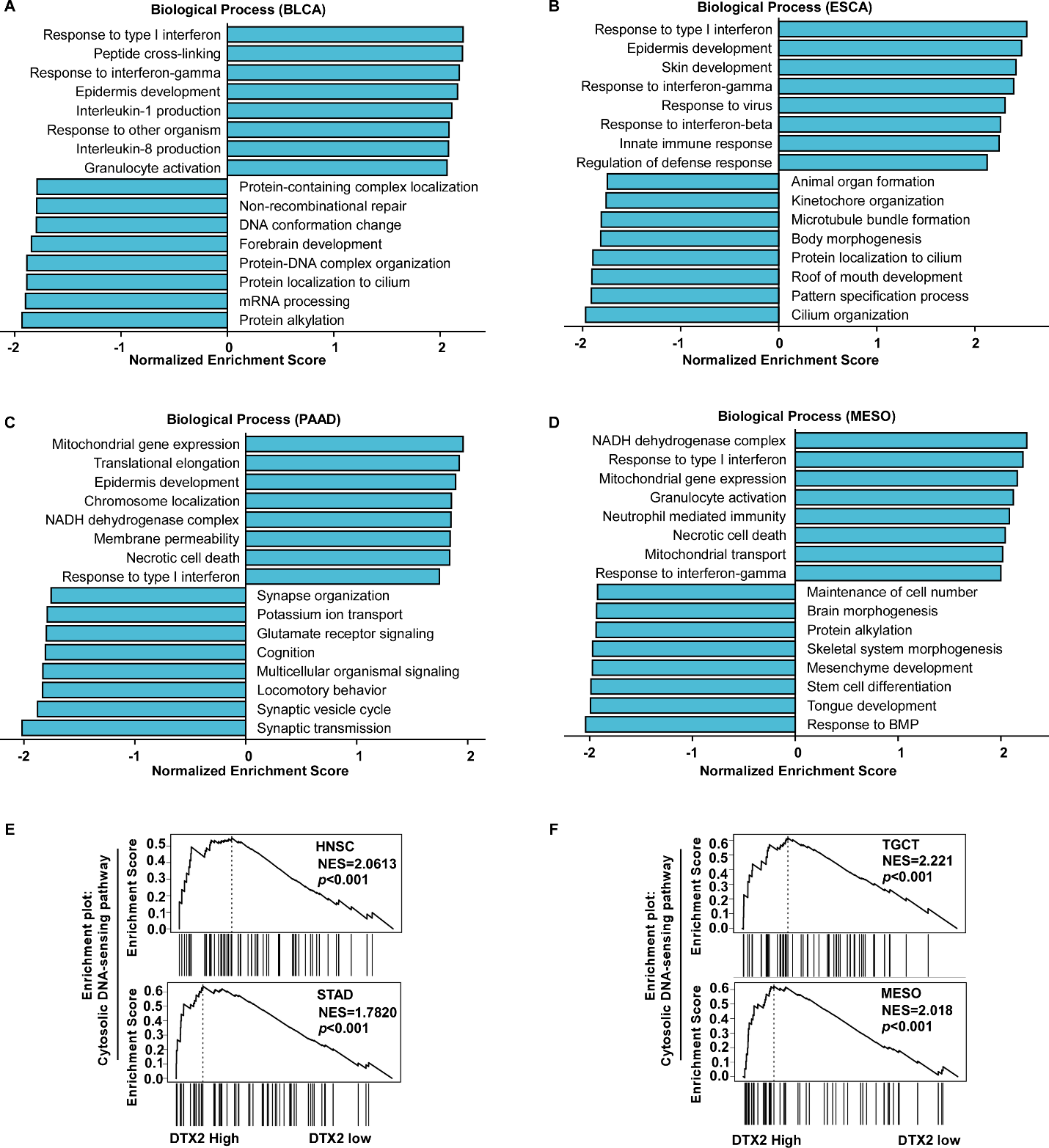


Figure S6. DTX2 expression correlates positively with the STING signal-related pathway across diverse tumor types. A-D) The biological processes associated with DTX2 were analyzed in BLCA, ESCA, PAAD, and MESO using GSEA, and the results are summarized. E-F) KEGG pathway analysis revealed a significant enrichment of the cytosolic DNA-sensing pathway in the DTX2-high group in HNSC, STAD, TGCT, and MESO.


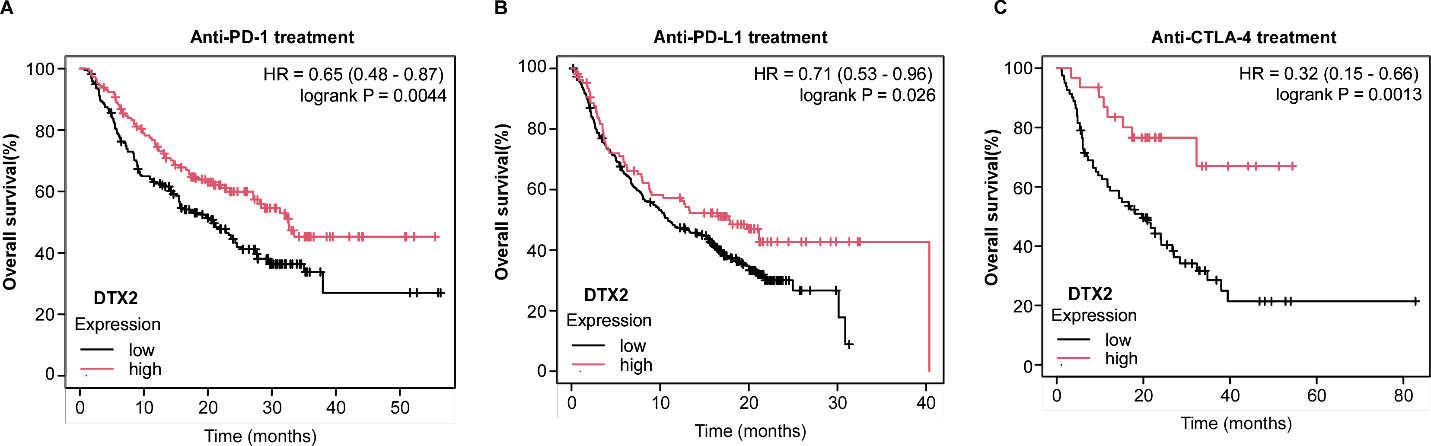


Figure S7. High DTX2 expression is associated with a favorable prognosis in tumor patients treated with immunotherapy. A-C) The correlation between DTX2 expression and Overall survival (OS) was assessed in cancer patients treated with PD-1 (A), PD-L1 (B), or CTLA-4 (C) blockade immunotherapy using the Kaplan-Meier Plotter database.


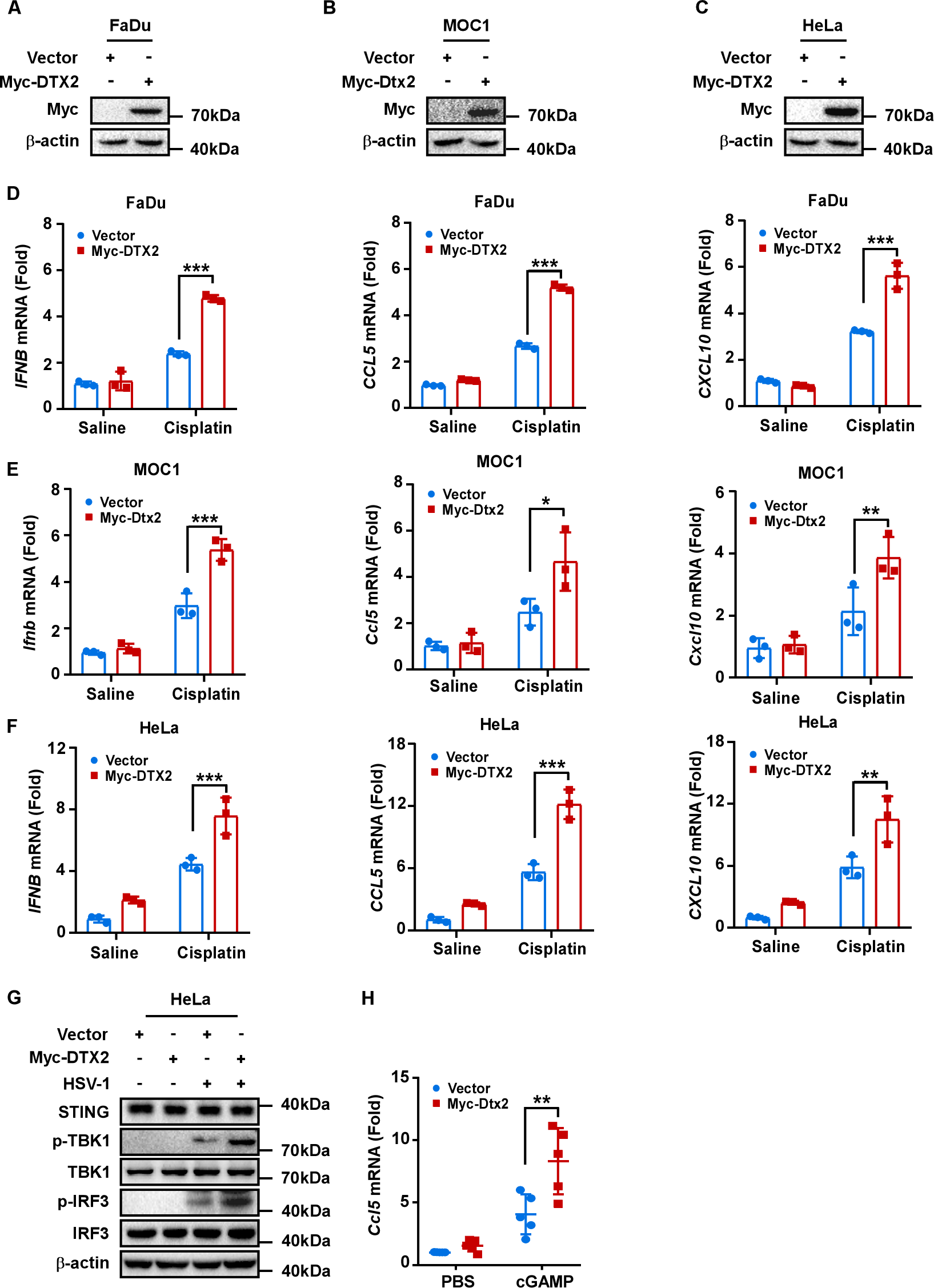


Figure S8. DTX2 enhances type I interferon response in tumor cells. A-C) The expression of DTX2 in FaDu, MOC1, and HeLa cells was detected by western blotting. D-F) RT-PCR was used to quantify the mRNA expression of *IFNB*, *CCL5,* and *CXCL10* in FaDu, MOC1, and HeLa cells with DTX2 overexpression following treatment with cisplatin. G) HeLa cells were stimulated with HSV-1. Cells were collected and lysed for western blotting. H) The expressions of *Ccl5* mRNA in each group of tumor tissues were quantified by RT-PCR. Data in (D-F; H) are shown as mean ± SD of three independent experiments. **p* < 0.05, ***p* < 0.01, ****p* < 0.001.


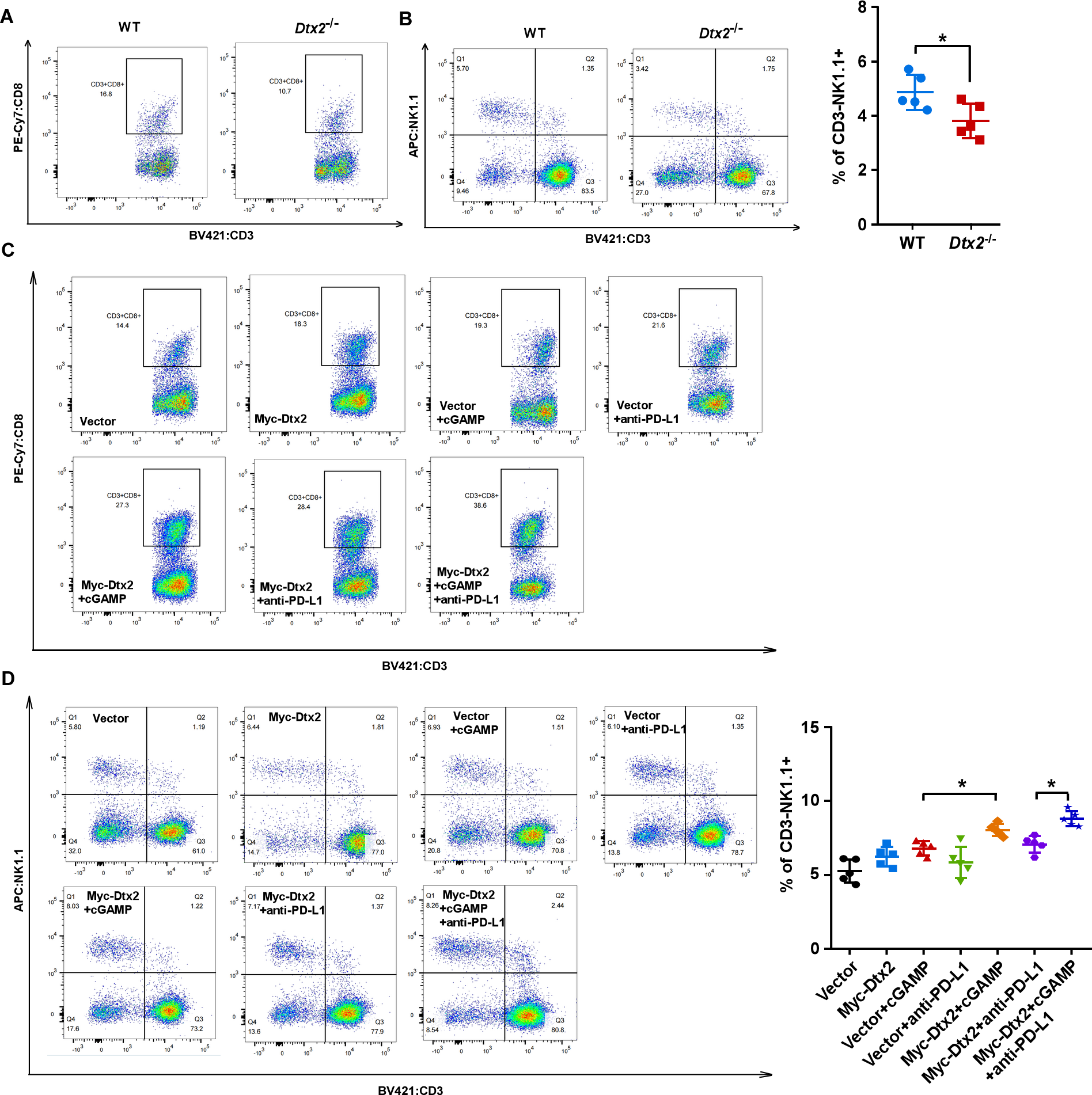


Figure S9. DTX2 promotes the infiltration of CD8T and NK cells in tumor tissues. A) Representative flow cytometry staining of CD8^+^CD3^+^ T cells in the labeled groups. B) Representative flow cytometry staining and the percentage of NK cells in the labeled groups. C) Representative flow cytometry staining of CD8^+^CD3^+^ T cells in in the labeled groups. D) Representative flow cytometry staining and the percentage of NK cells in the labeled groups. n=5 mice/group. **p* < 0.05.
