# Supplementary material for: Deltex E3 ubiquitin ligase 2 potentiates STING-mediated type I interferon response by K63-linked ubiquitination
Source: Cell Death Dis. 2026 Mar 28;17(1):424. doi: 10.1038/s41419-026-08659-4 (PMC13150011; doi:10.1038/s41419-026-08659-4)
Supplement: Supplementary file 2 — Supplementary Table [file 41419_2026_8659_MOESM2_ESM.docx]

| Table S1 The specific primers used for quantitative PCR are listed below | |
| --- | --- |
| Primer | Sequence (5'-3') |
| HSV-F | CAGTGCTTCAGCCGCTACCC |
| HSV-R | TGCCGTTCTTCTGCTTGTCG |
| M-Cxcl10-F | CCAAGTGCTGCCGTCATTTTC |
| M-Cxcl10-R | GGCTCGCAGGGATGATTTCAA |
| M-Ccl5-F | GCTGCTTTGCCTACCTCTCC |
| M-Ccl5-R | TCGAGTGACAAACACGACTGC |
| M-Ifnb-F | ATGGTGGTCCGAGCAGAGAT |
| M-Ifnb-R | CCACCACTCATTCTGAGGCA |
| M-Gapdh-F | AACTTTGGCATTGTGGAAGG |
| M-Gapdh-R | ACACATTGGGGGTAGGAACA |
| H-CXCL10-F | GTGGCATTCAAGGAGTACCTC |
| H-CXCL10-R | TGATGGCCTTCGATTCTGGATT |
| H-CCL5-F | CCAGCAGTCGTCTTTGTCAC |
| H-CCL5-R | CTCTGGGTTGGCACACACTT |
| H-IFNB-F | ATGACCAACAAGTGTCTCCTCC |
| H-IFNB-R | GGAATCCAAGCAAGTTGTAGCTC |
| H-STING-F | CCAGAGCACACTCTCCGGTA |
| H-STING-R | CGCATTTGGGAGGGAGTAGTA |
| H-ACTIN-F | GCCGGGACCTGACTGACTAC |
| H-ACTIN-R | CGGATGTCCACGTCACACTT |
