# Supplementary material for: Deltex E3 ubiquitin ligase 2 potentiates STING-mediated type I interferon response by K63-linked ubiquitination
Source: Cell Death Dis. 2026 Mar 28;17(1):424. doi: 10.1038/s41419-026-08659-4 (PMC13150011; doi:10.1038/s41419-026-08659-4)

Figure 1D

1.Dtx2

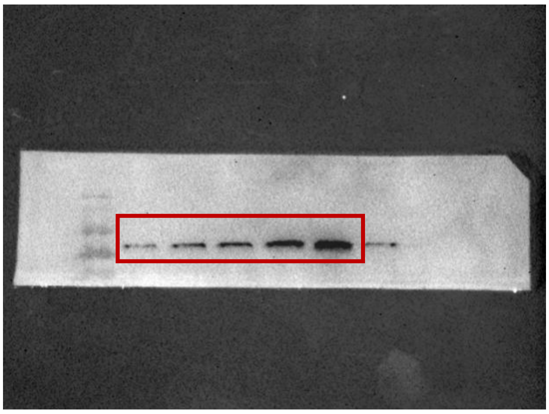

2.β-actin

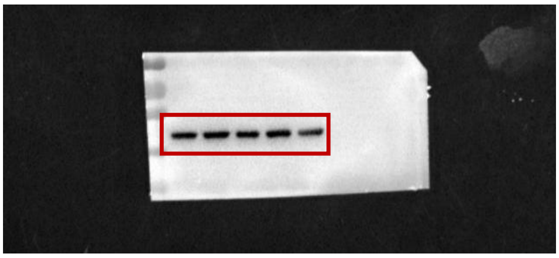

Figure 1 F

1.Dtx2

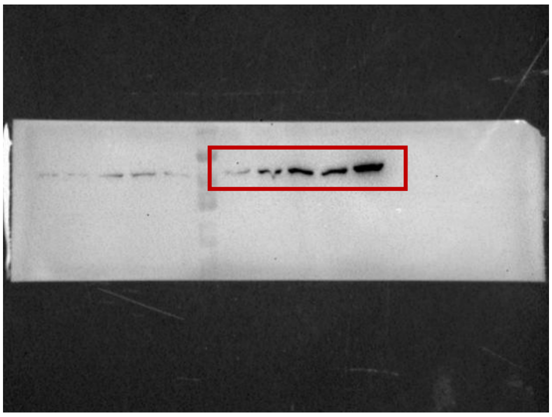

2.β-actin

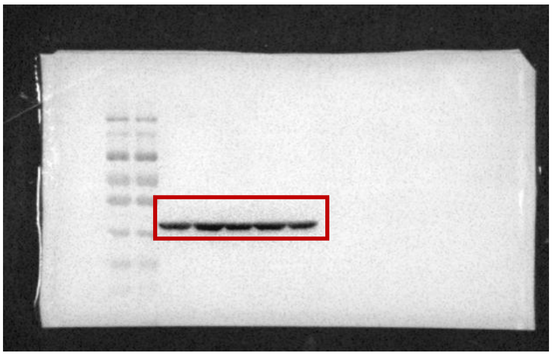

Figure 2 A

1.STING

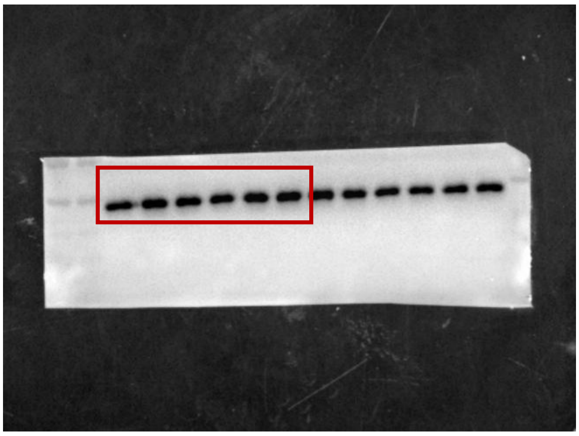

2.p-TBK1

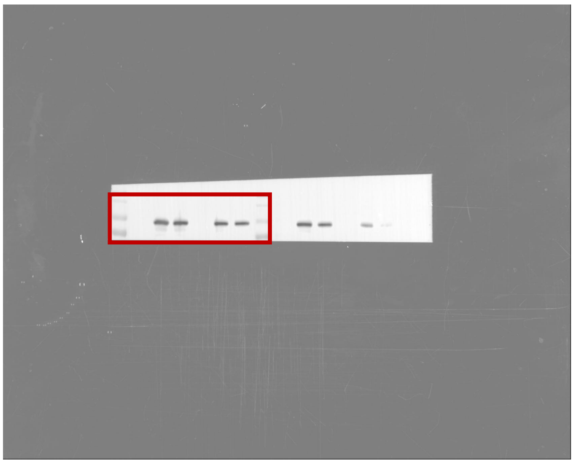

3.TBK1

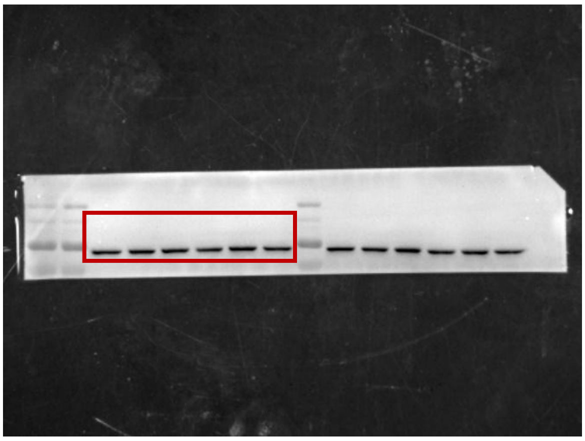

4.p-p65

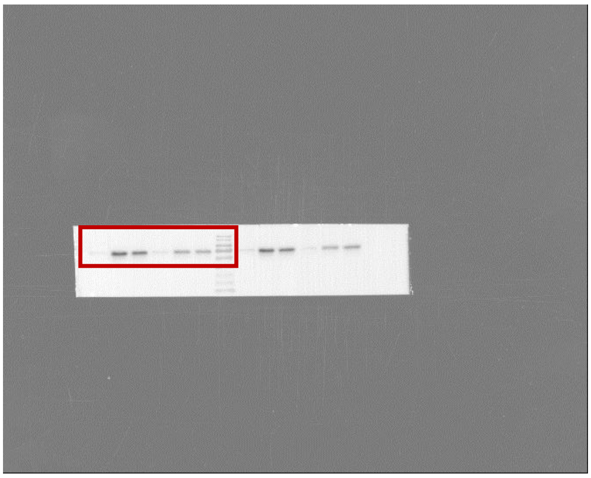

5.p65

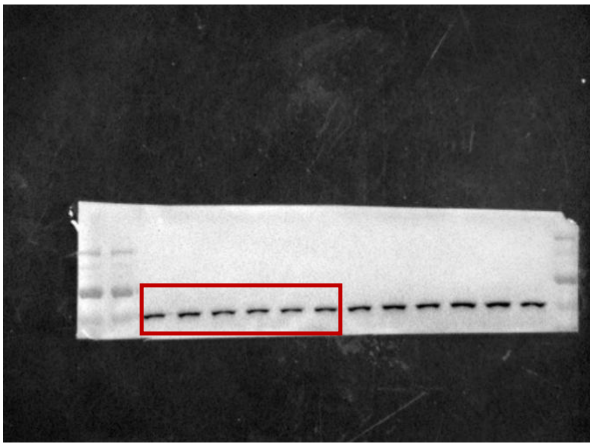

6.p-IRF3

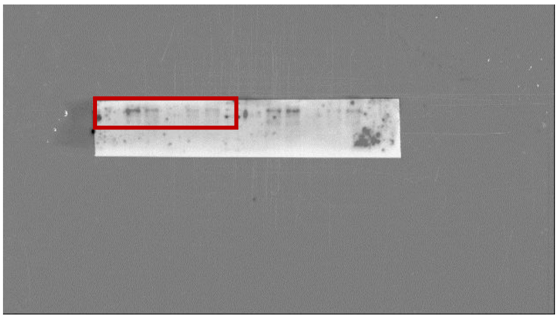

7.IRF3

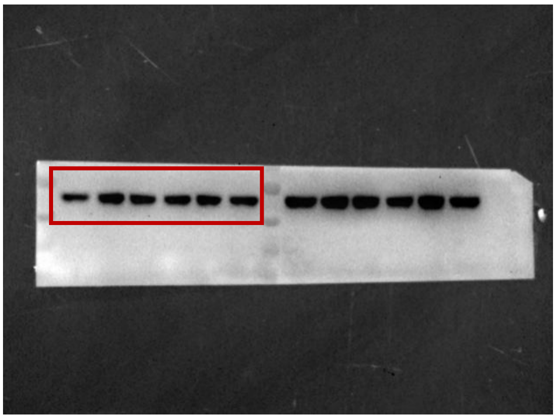

8.β-actin

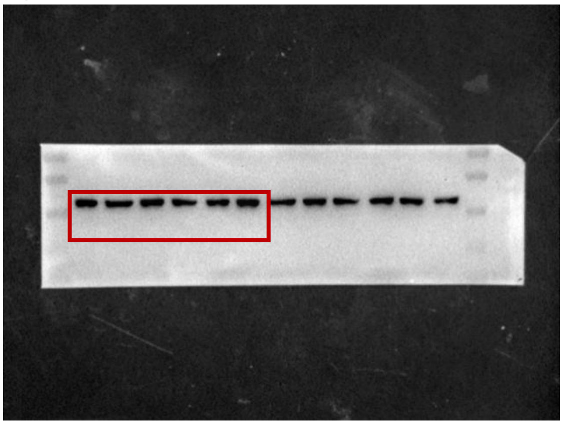

Figure 2 B

1.STING

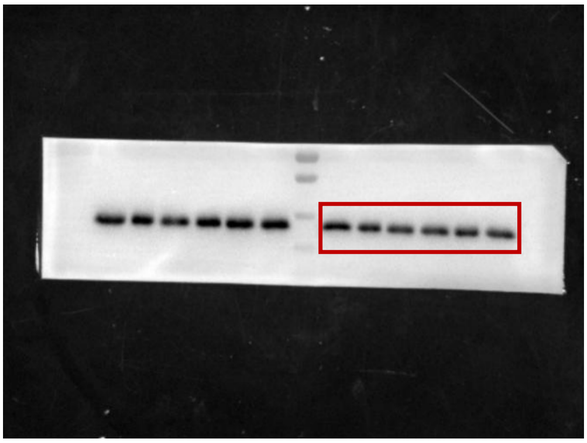

2.p-TBK1

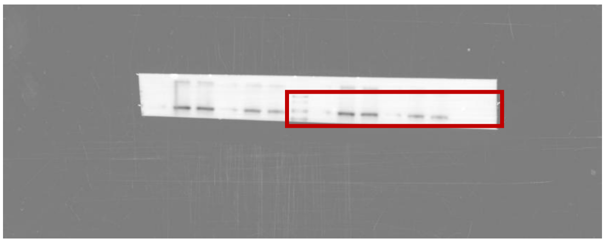

3.TBK1

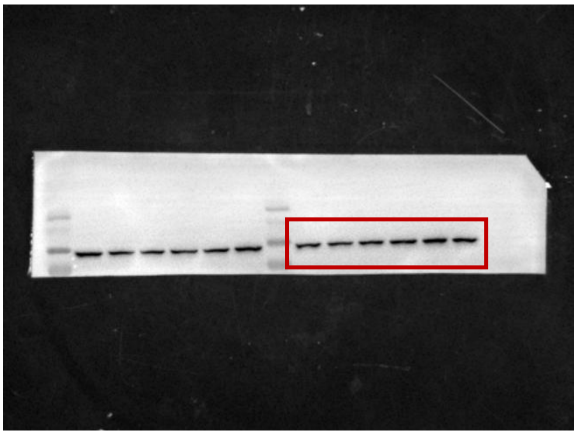

4.p-p65

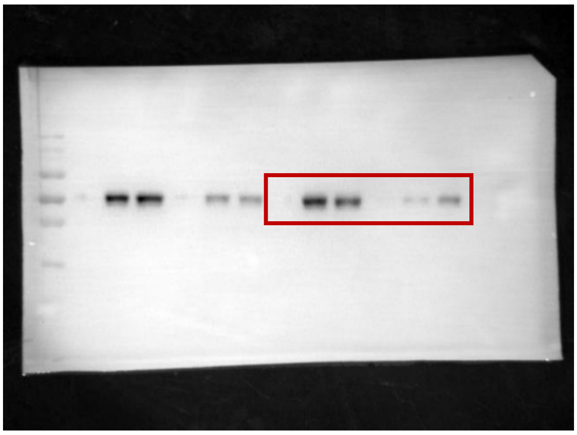

5.p65

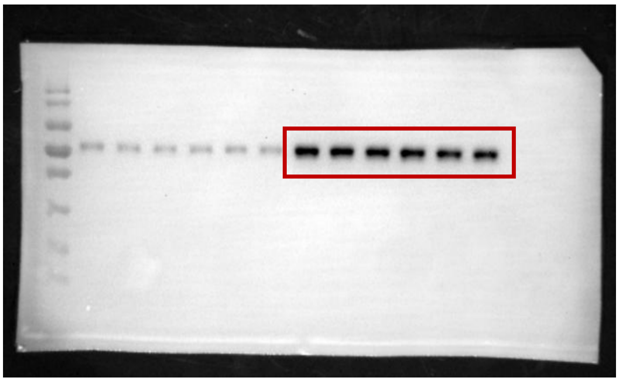

6.p-IRF3

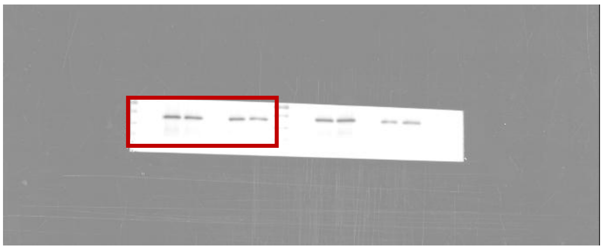

7.IRF3

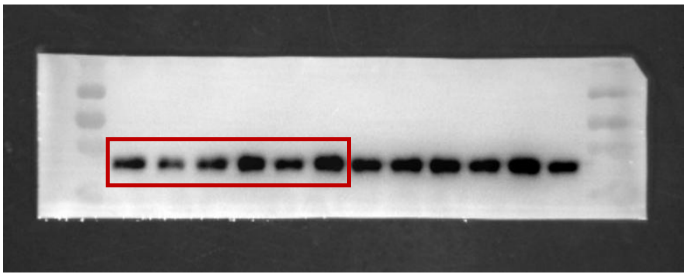

8.β-actin

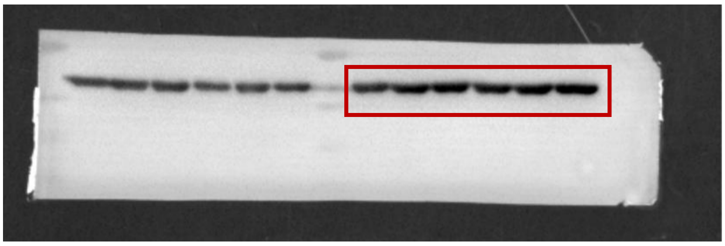

Figure 2 C

1.STING

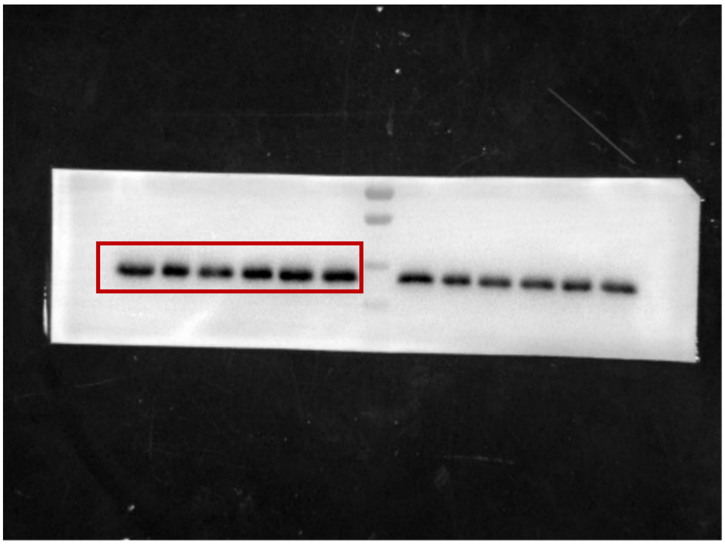

2.p-TBK1

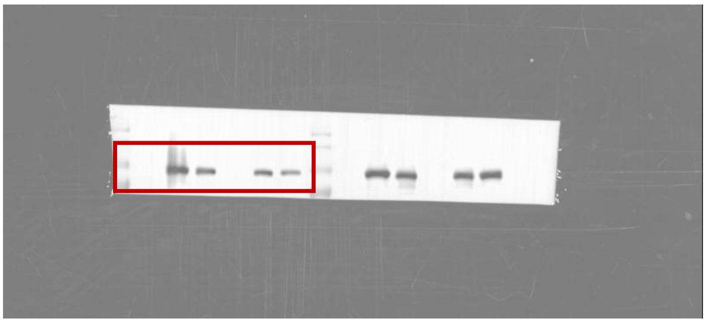

3.TBK1

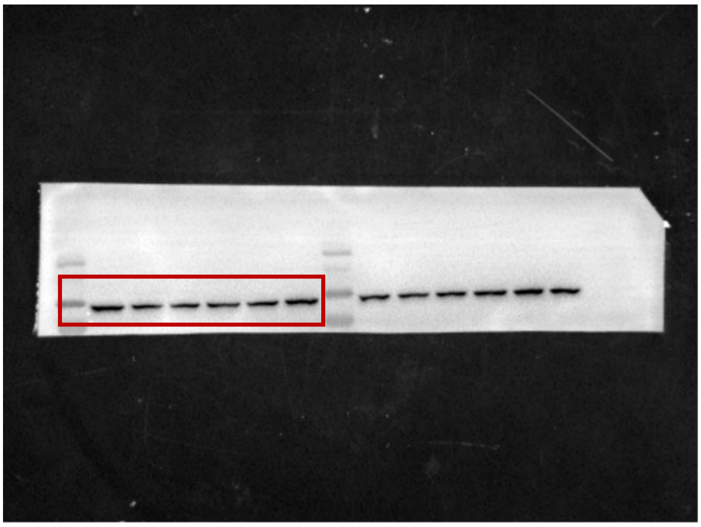

4.p-p65

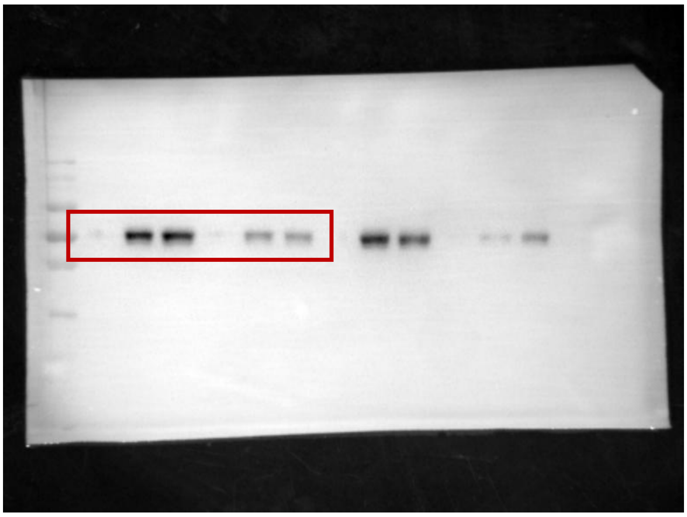

5.p65

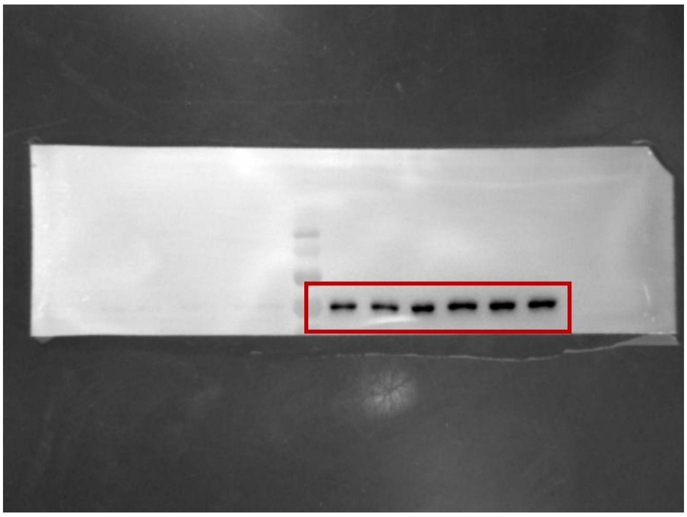

6.p-IRF3

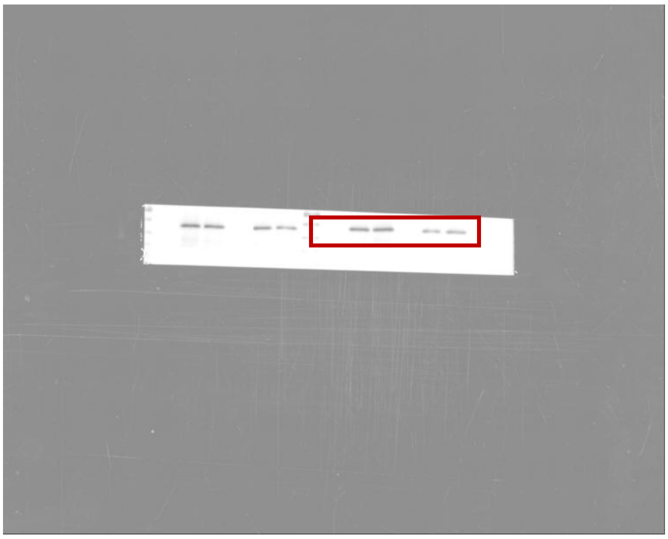

7.IRF3

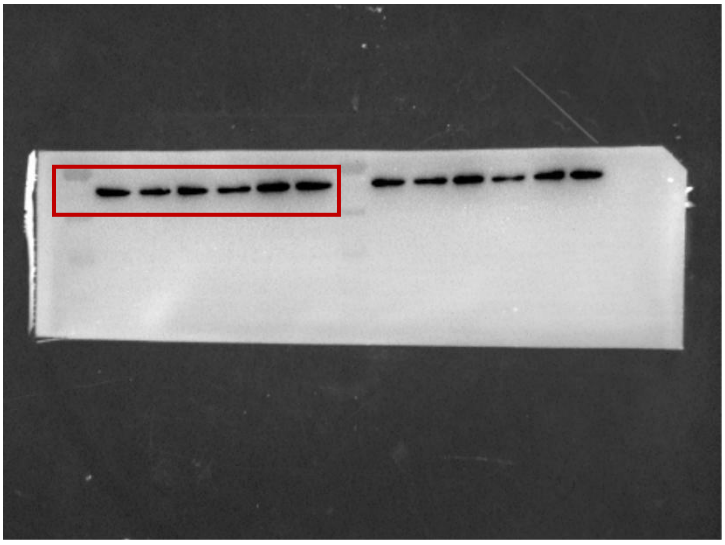

8.β-actin

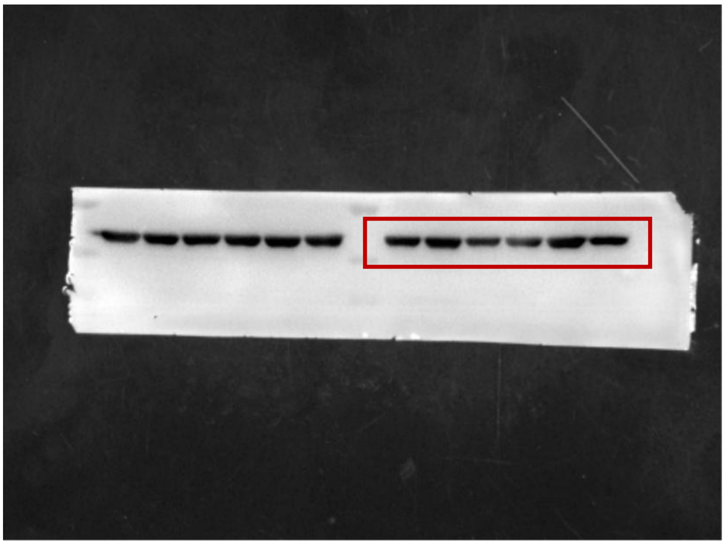

Figure 2 D

1.STING

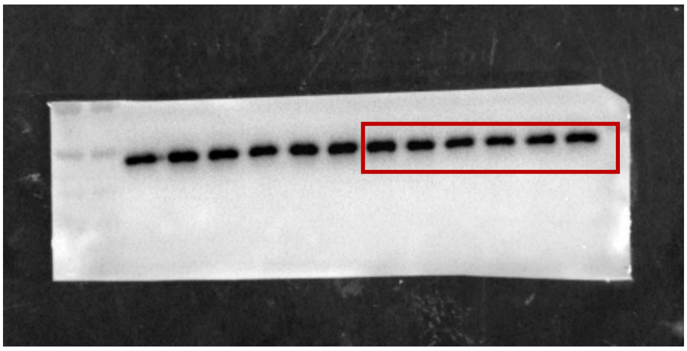

2. p-TBK1

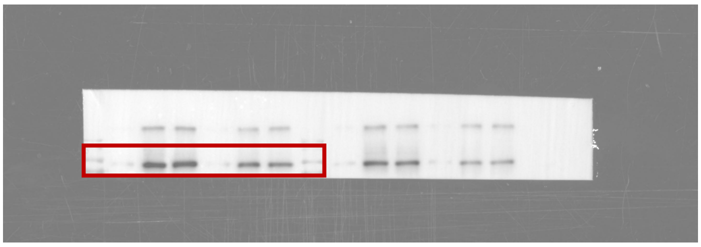

3.TBK1

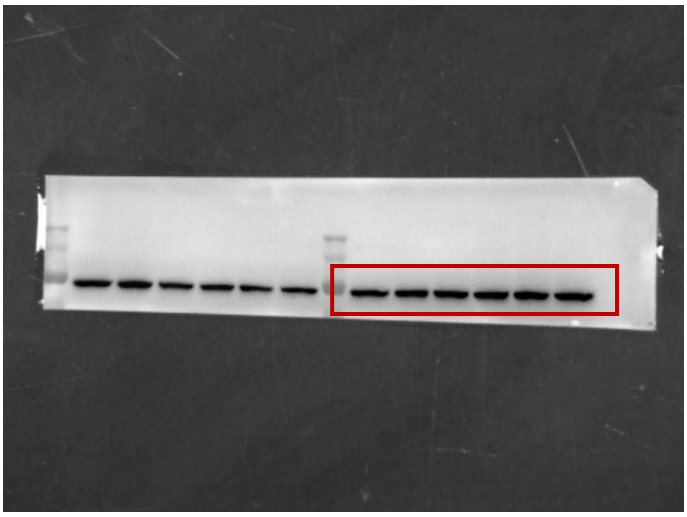

4.p-p65

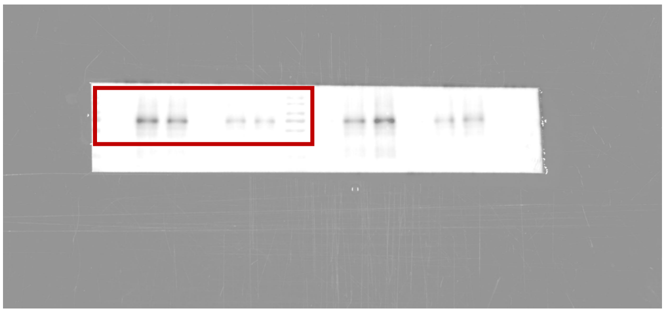

5.p65

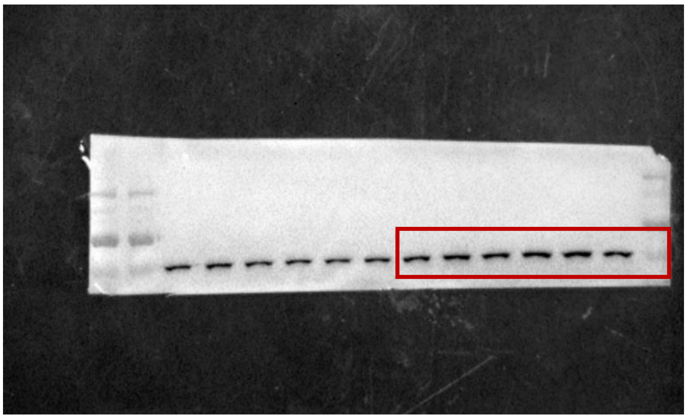

6.p-IRF3

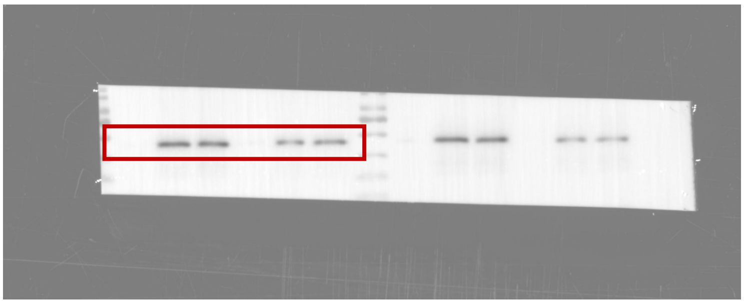

7.IRF3

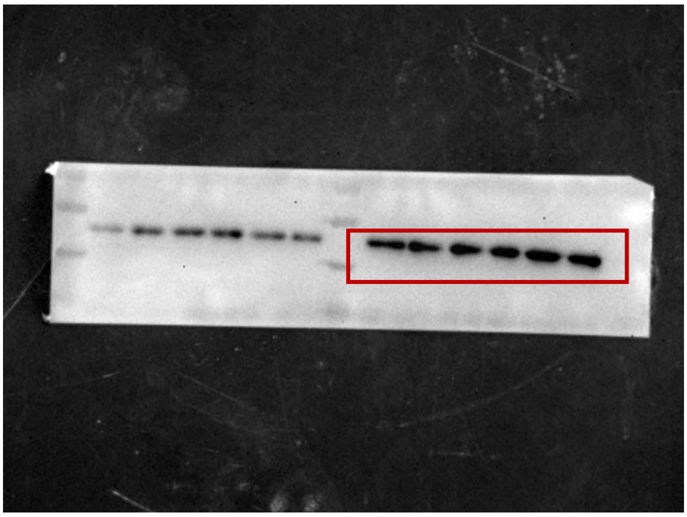

8.β-actin

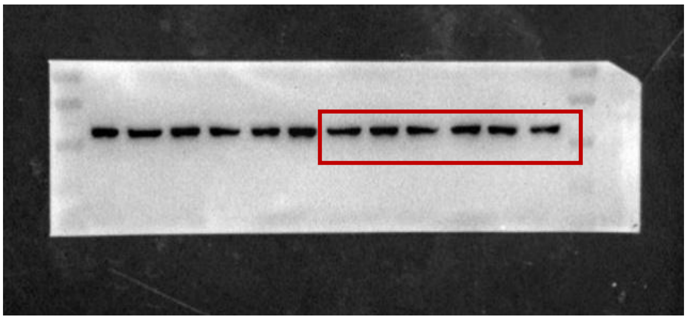

Figure 2 E

1. STING

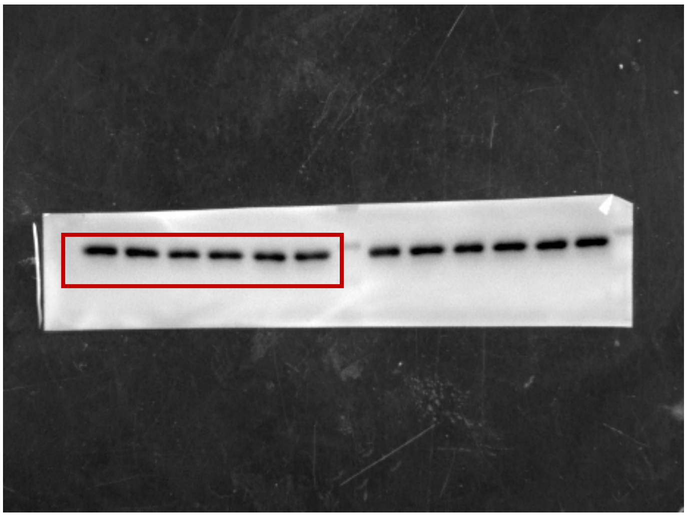

2.p-TBK1

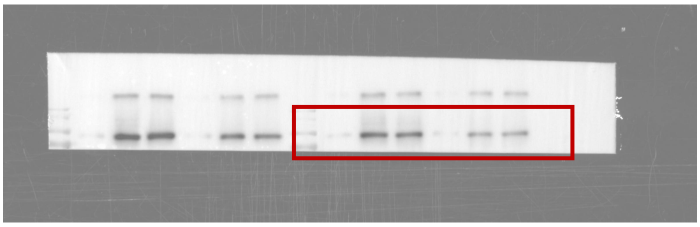

3.TBK1

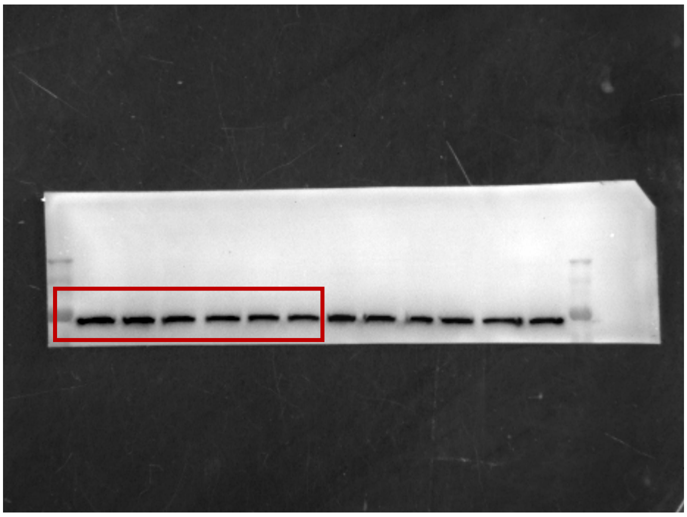

4.p-p65

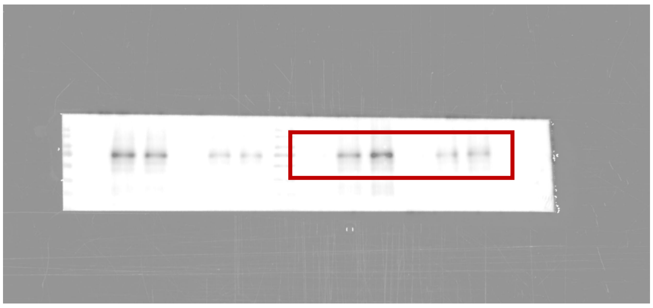

5.p65

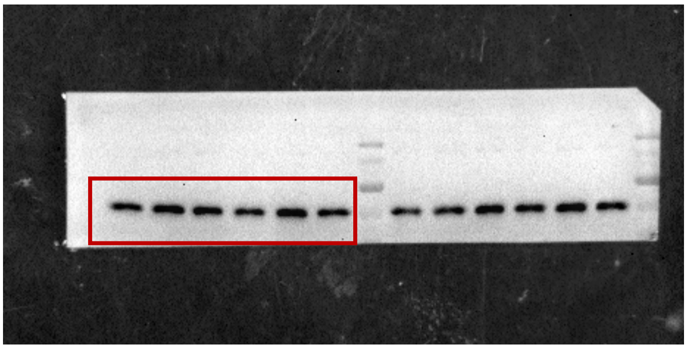

6.p-IRF3

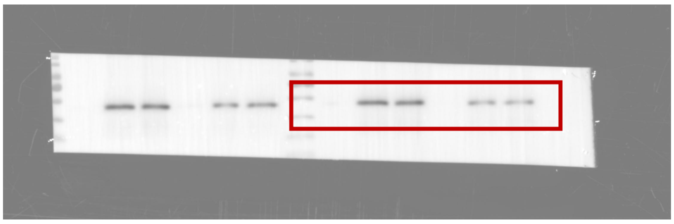

7.IRF3

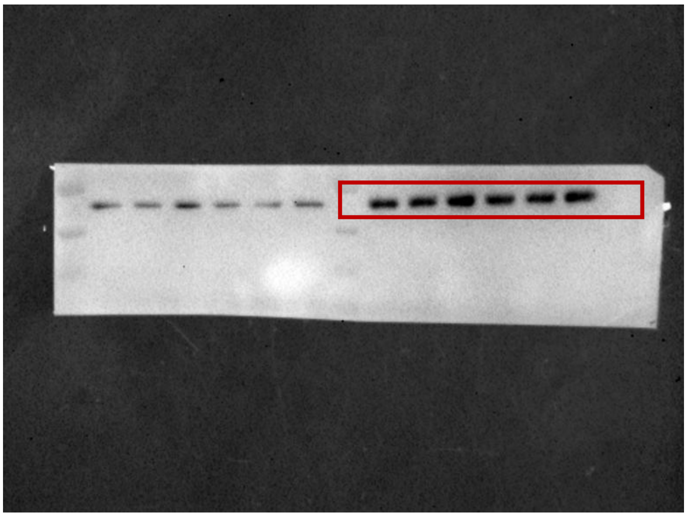

8.β-actin

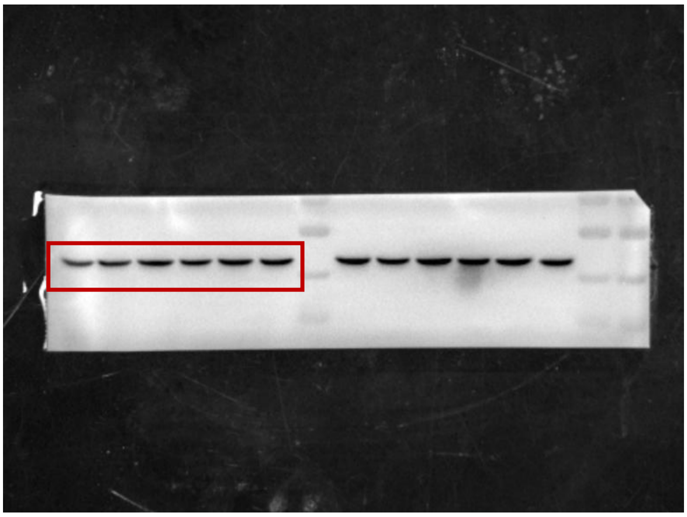

Figure 2 F

1. STING

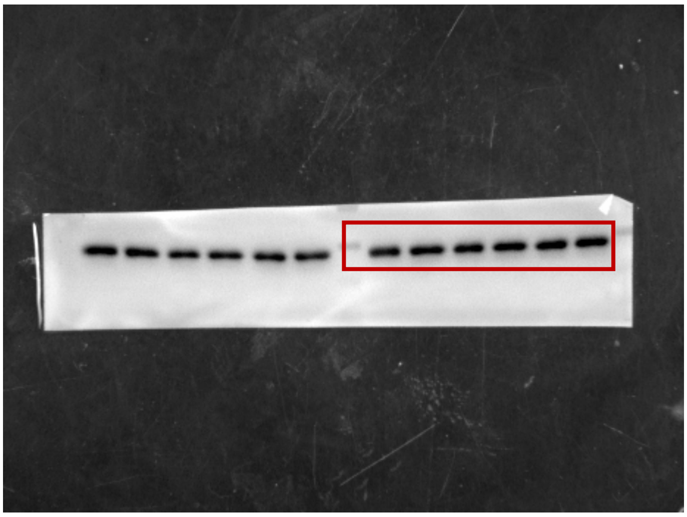

2.p-TBK1

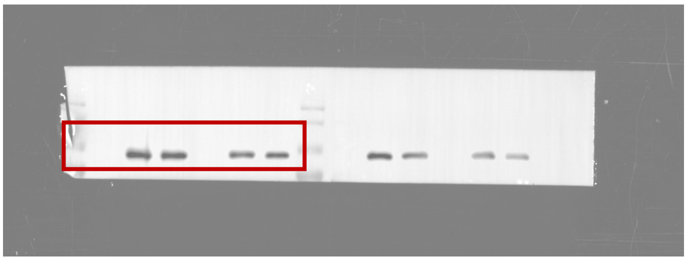

3.TBK1

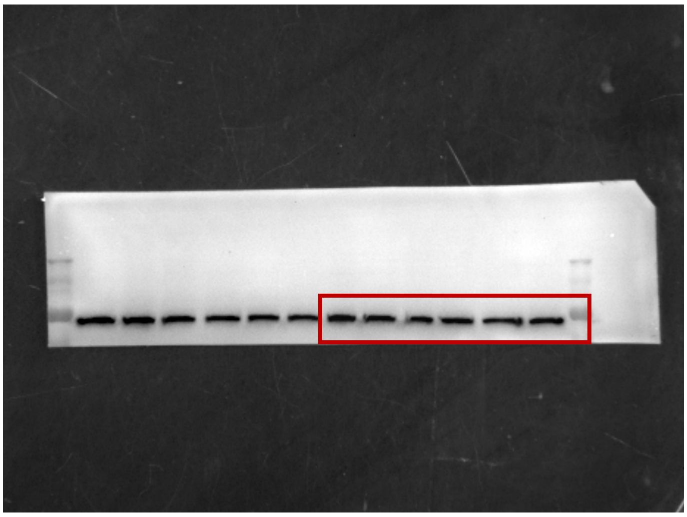

4.p-p65

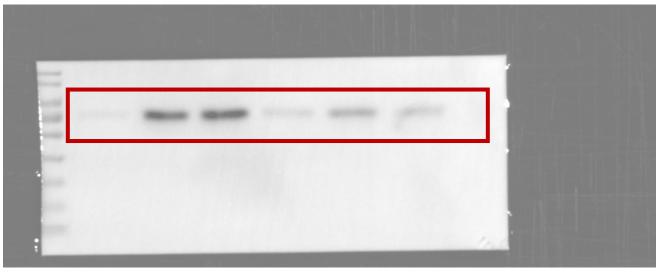

5.p65

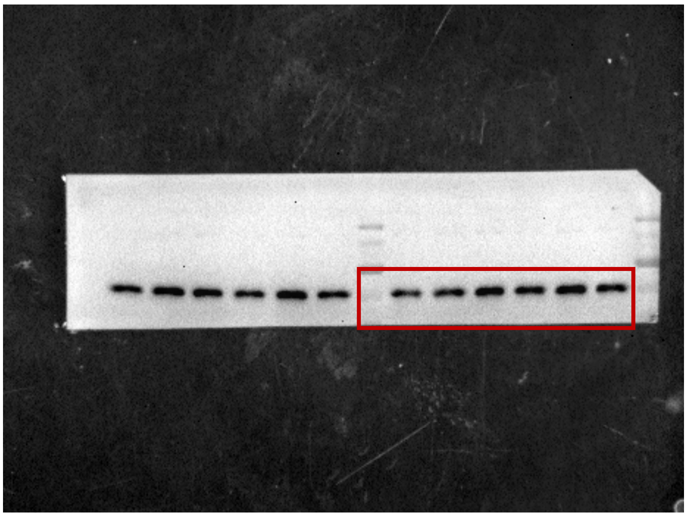

6.p-IRF3

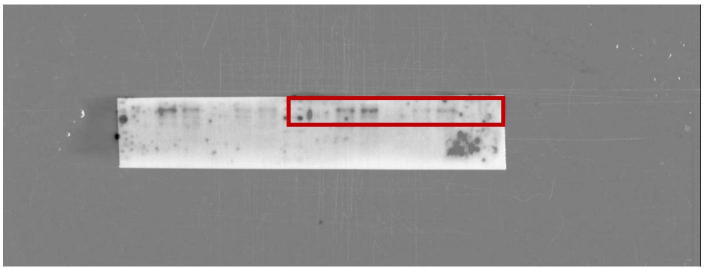

7.IRF3

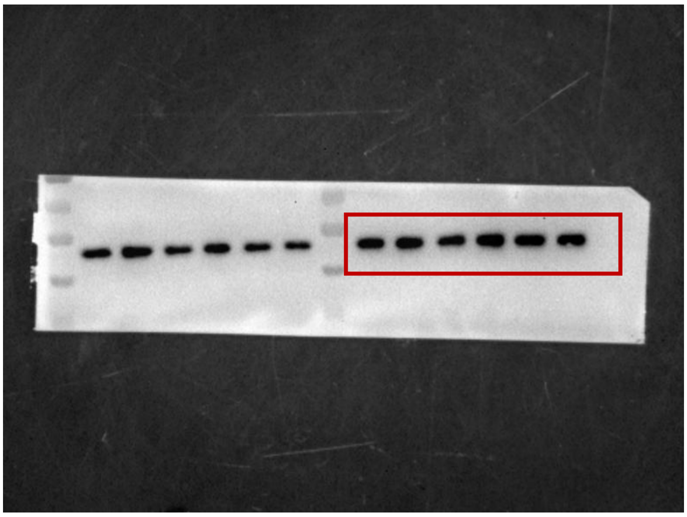

8.β-actin

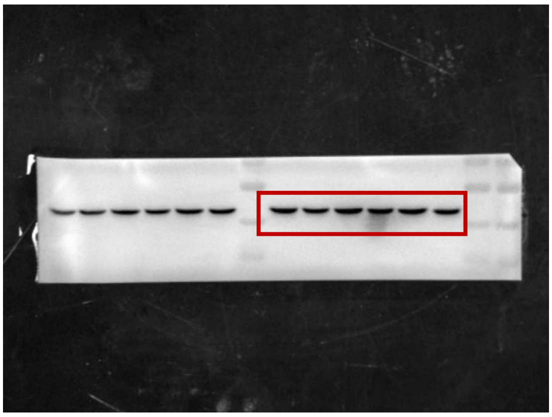

Figure 2 G

1.IRF3

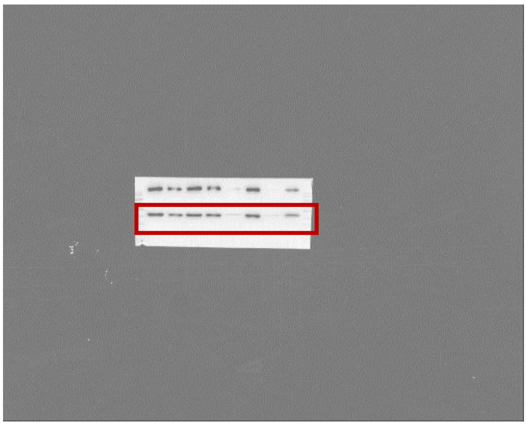

2.p65

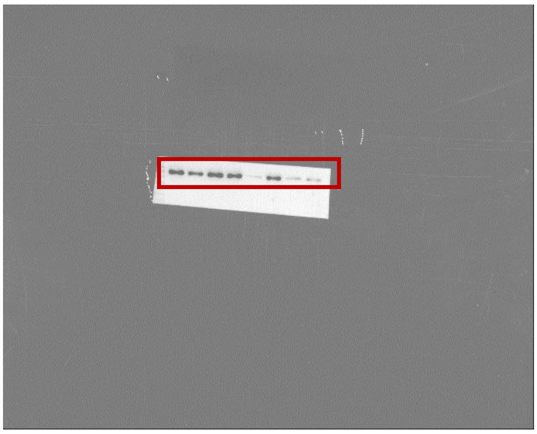

3.H3

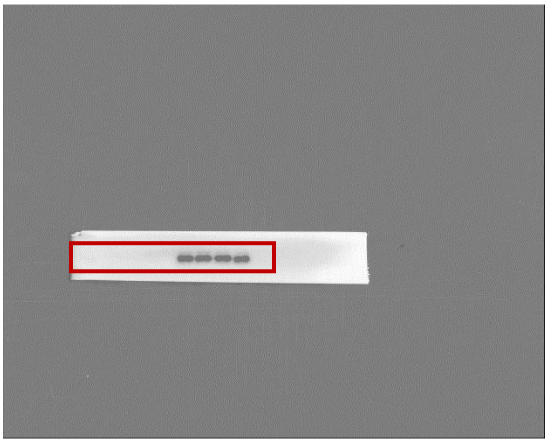

4.β-actin

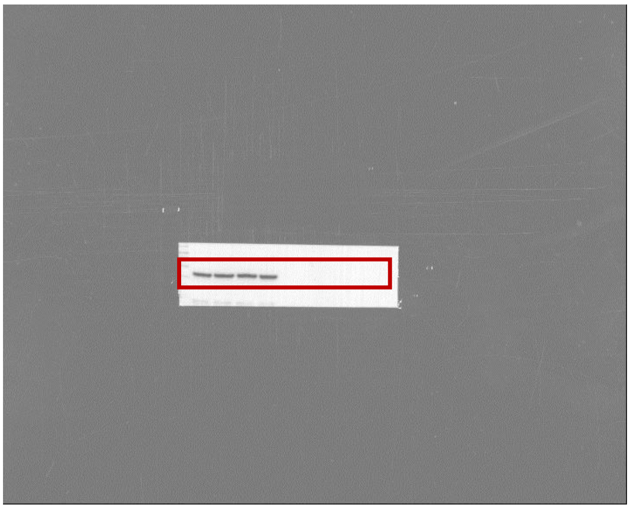

Figure 2 H

1.IRF3

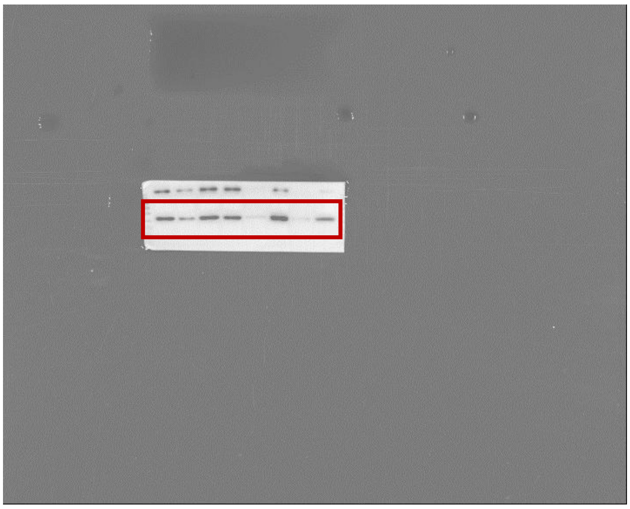

2.p65

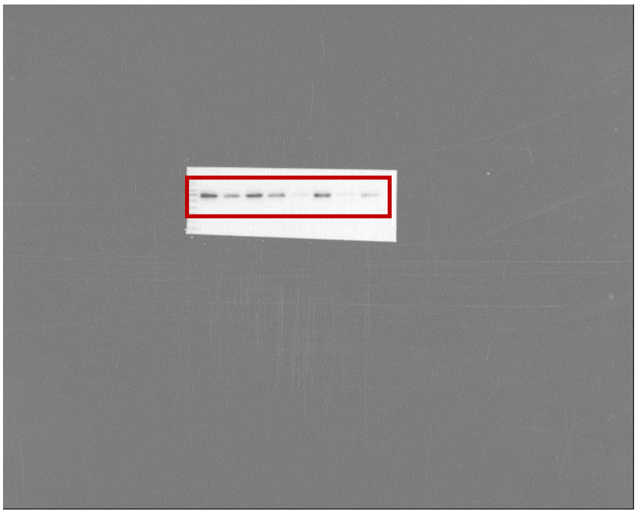

3.H3

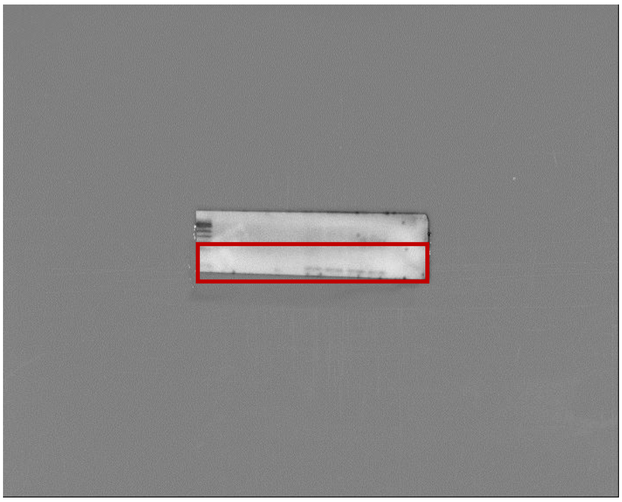

4.β-actin

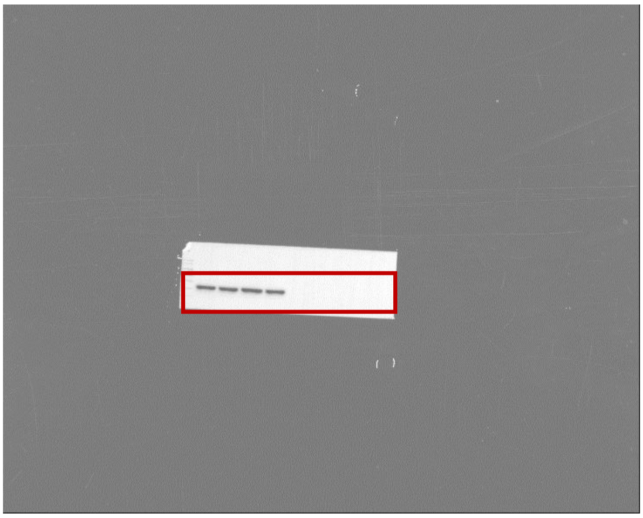

Figure 2 I

1.IRF3

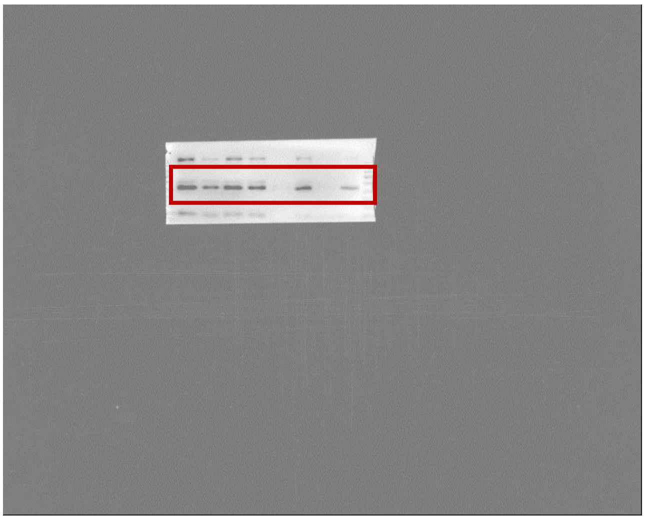

2.p65

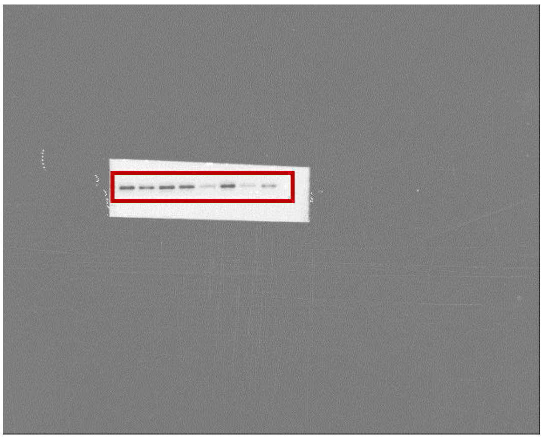

3.H3

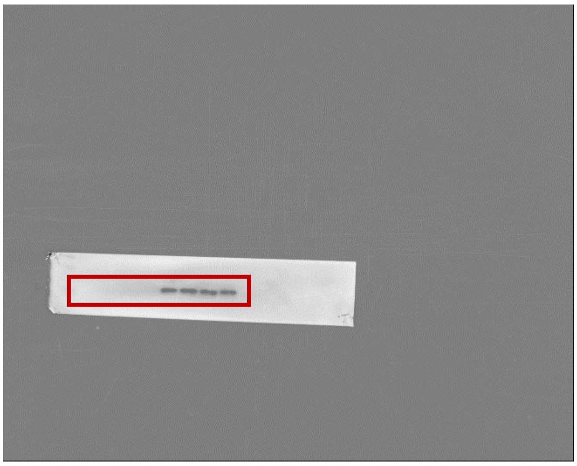

4.β-actin

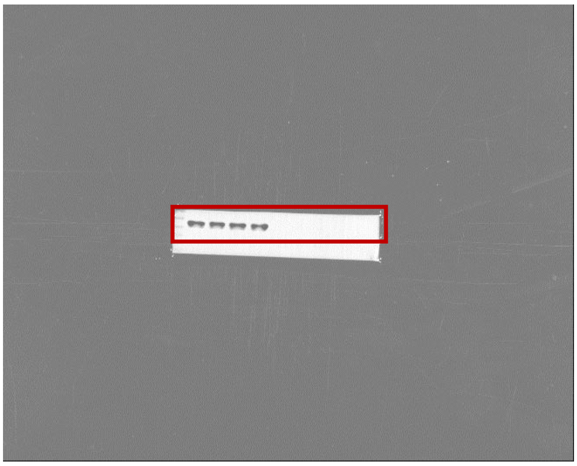

Figure 3 B

1.IP:Myc-DTX2

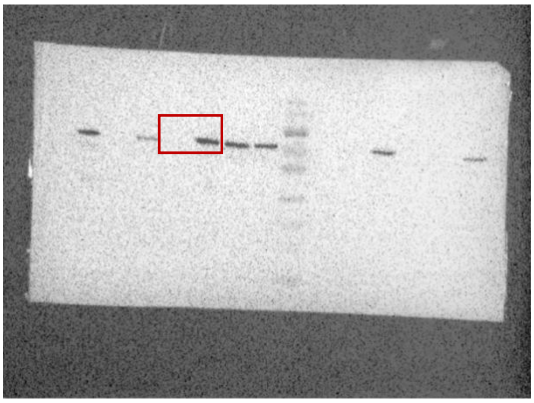

2.IP:Flag-STING

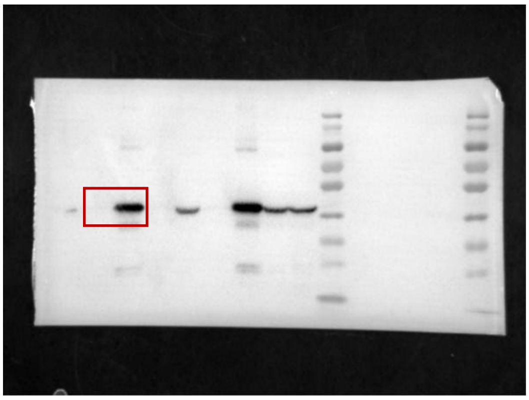

3.Input: Myc-DTX2

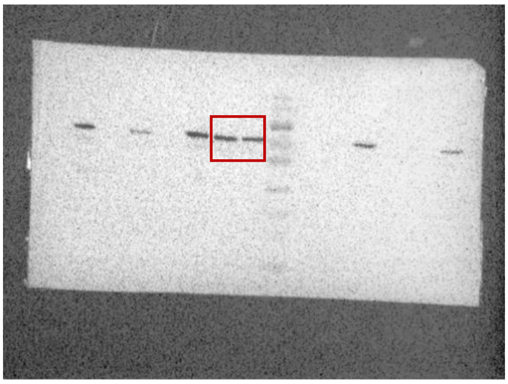

4.Input: Flag-STING

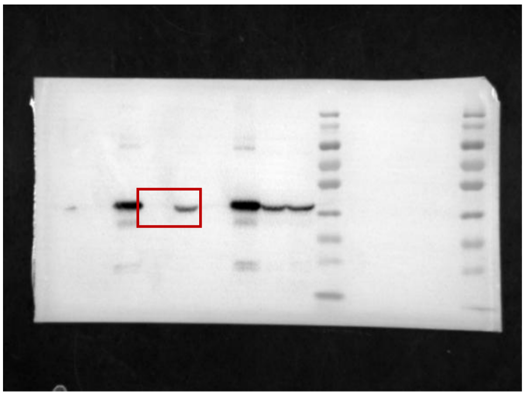

5. Input:β-actin

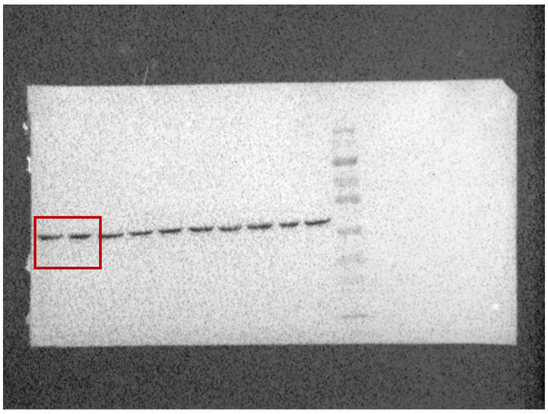

Figure 3 C

1. IP:Flag-STING

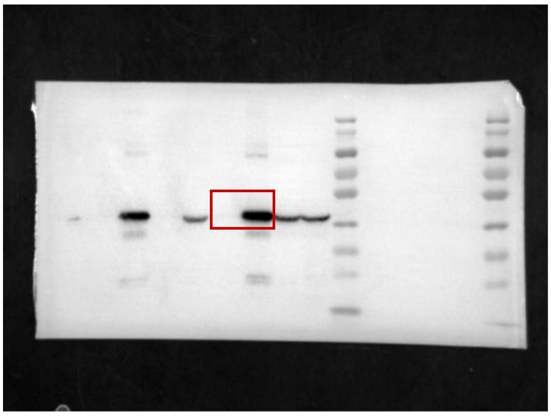

2. IP:Myc-DTX2

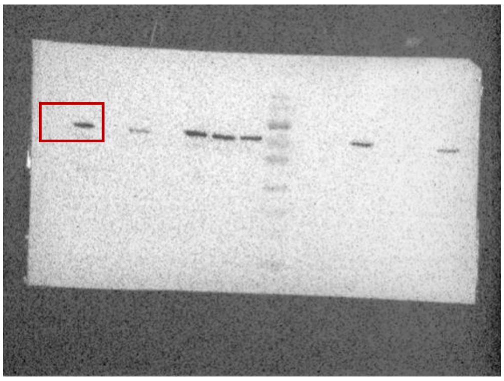

3. Input: Flag-STING

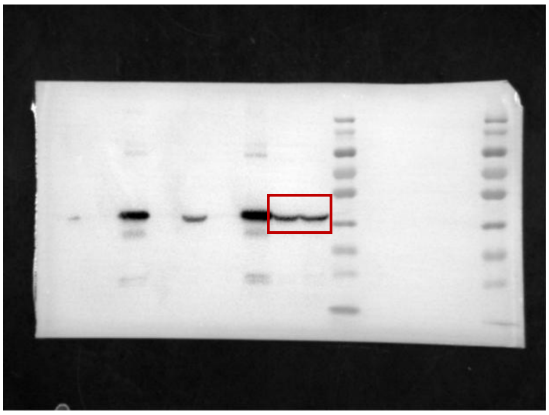

4. Input: Myc-DTX2

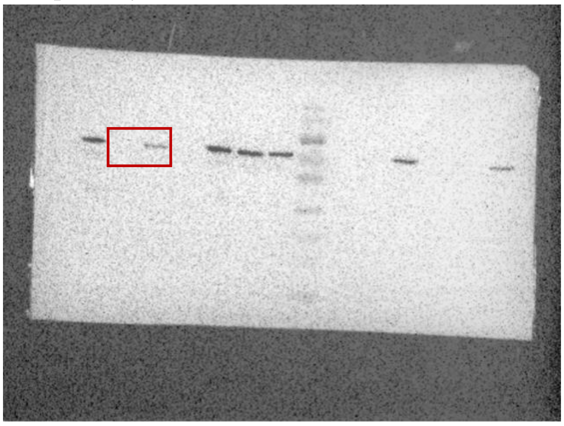

5. Input:β-actin

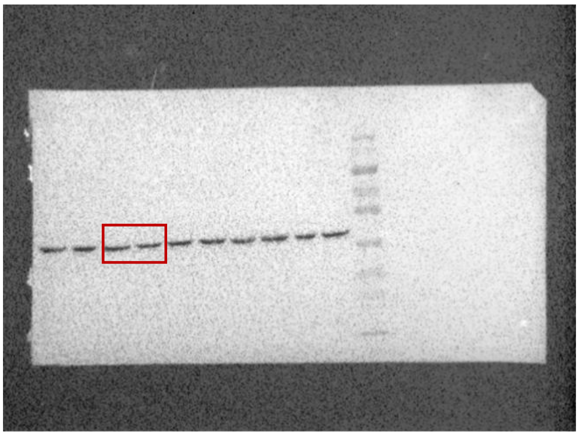

Figure 3 D

1.IP: Dtx2

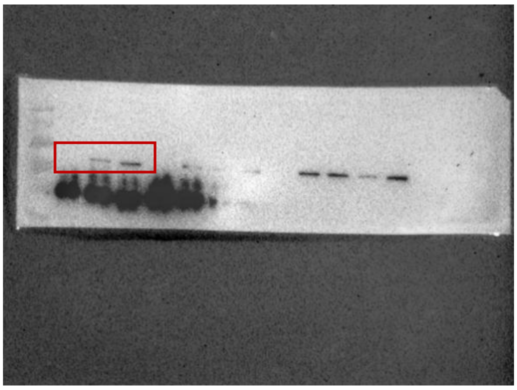

2.IP: STING

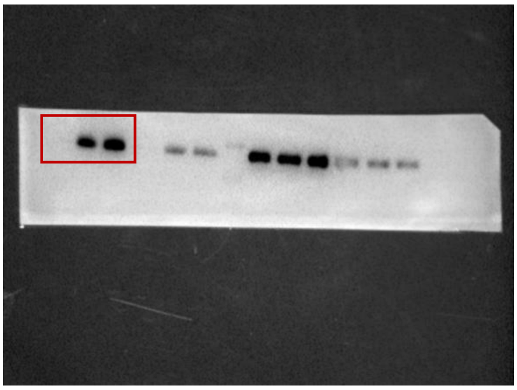

3.Input: Dtx2

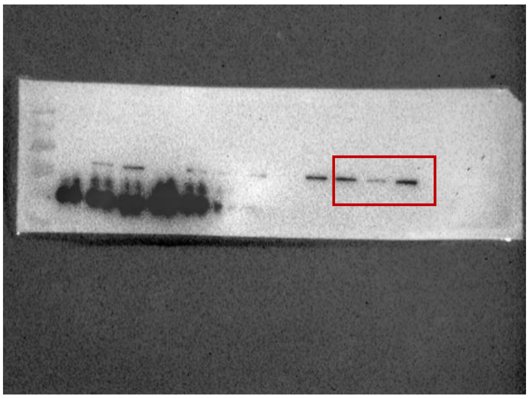

4.Input: STING

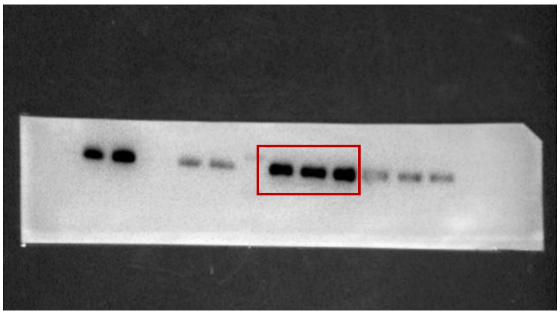

5. Input:β-actin

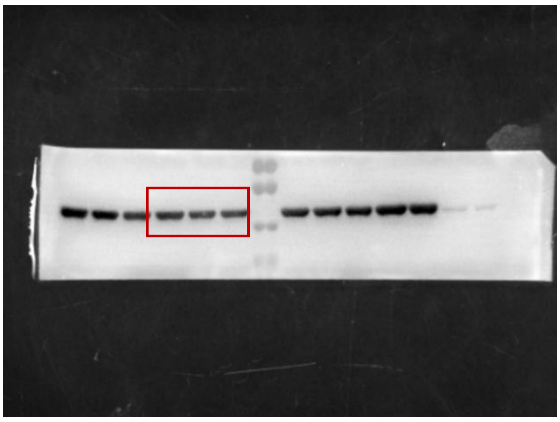

Figure 3 E

1.IP: Dtx2

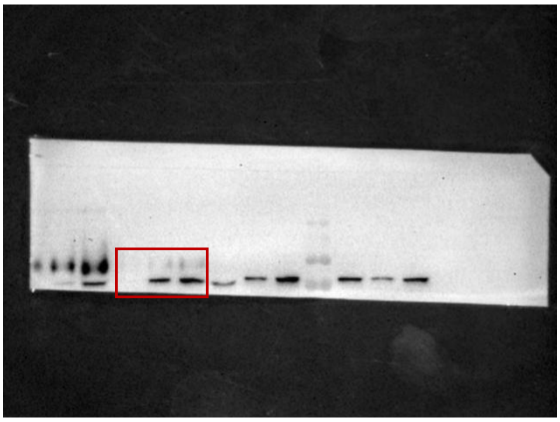

2.IP: STING

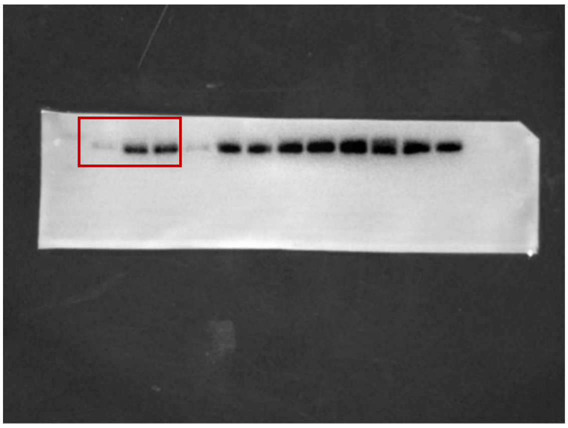

3.Input: Dtx2

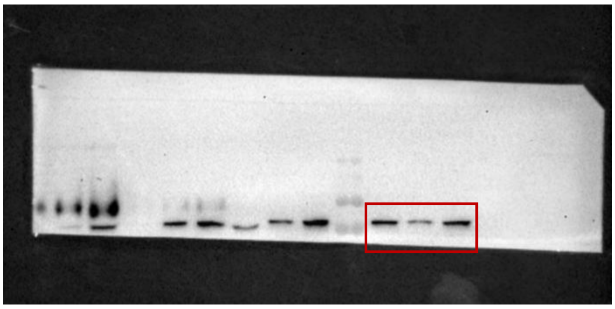

4.Input: STING

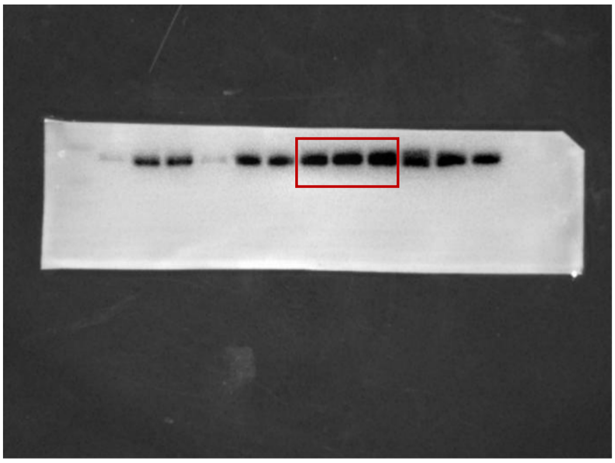

5. Input:β-actin

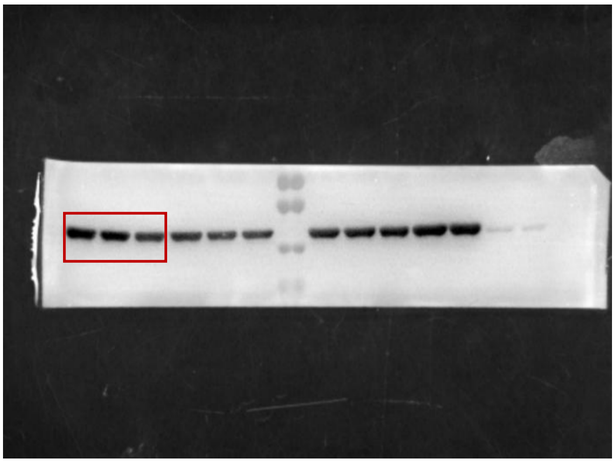

Figure 3 F

1. GST-Pull-down:His

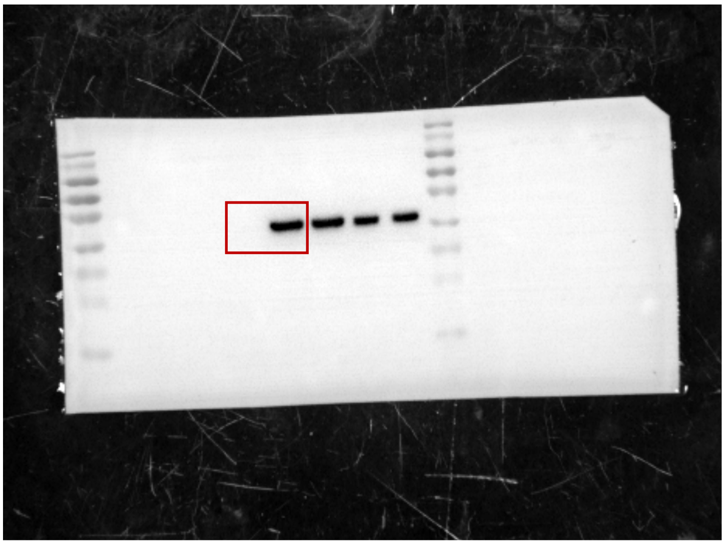

2.GST-Pull-down:GST

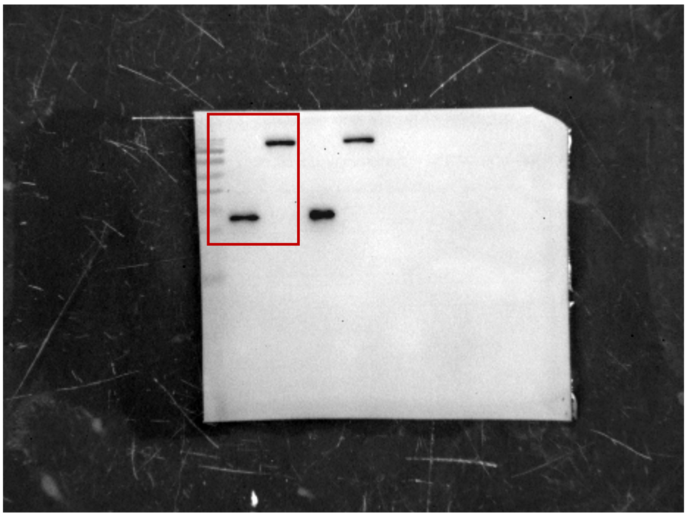

3.Lysate:His

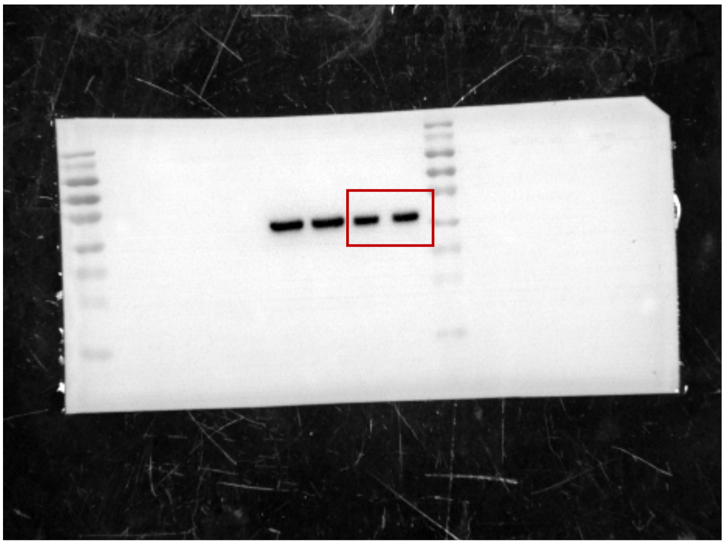

4.Lysate:GST

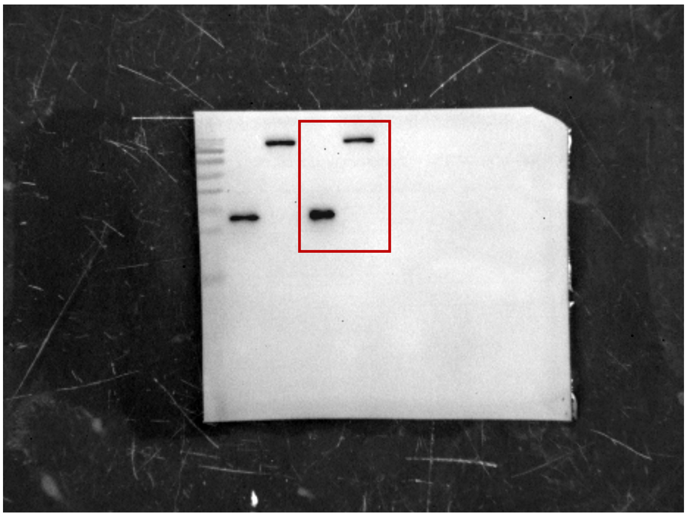

Figure 3 H

1. IP:Flag

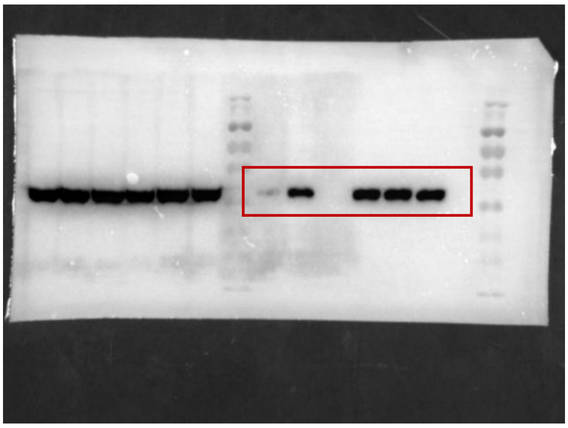

2. IP:Myc

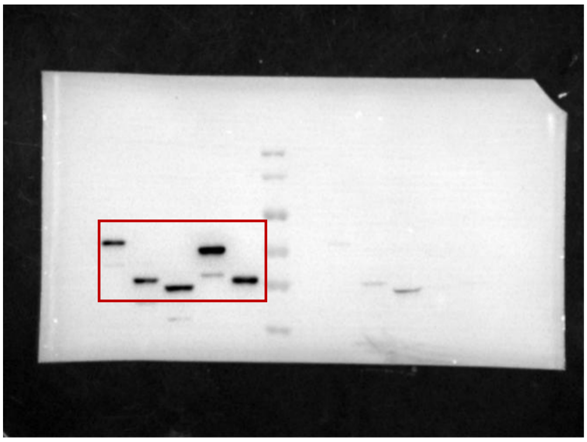

3. Input: Flag

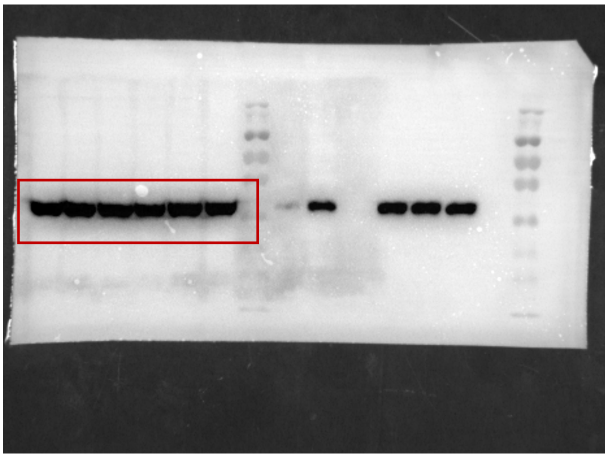

4. Input: Myc

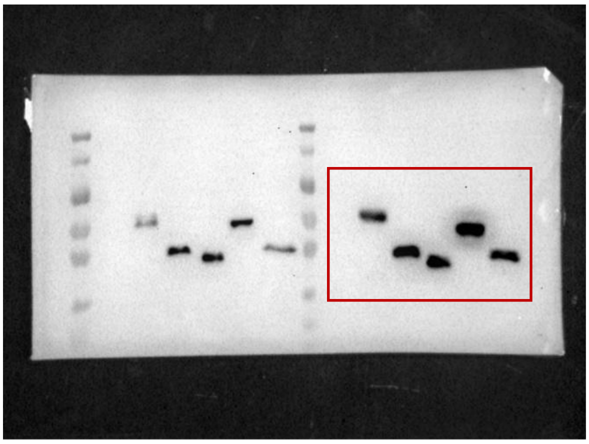

5. Input:β-actin

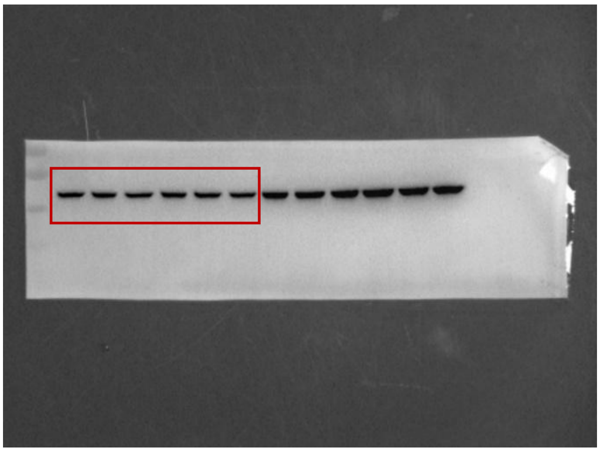

Figure 3 I

1.IP:Myc

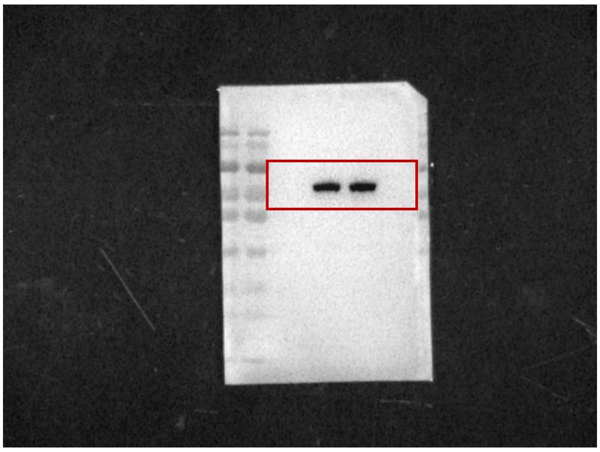

2.IP:Flag

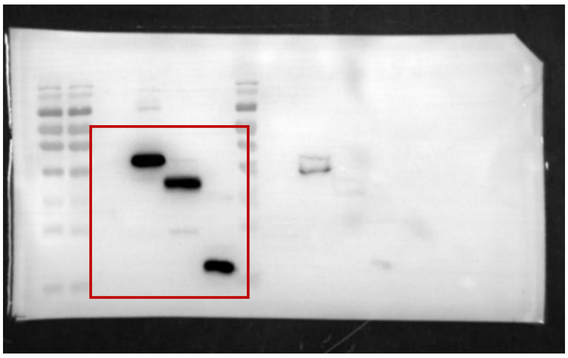

3.Input: Myc

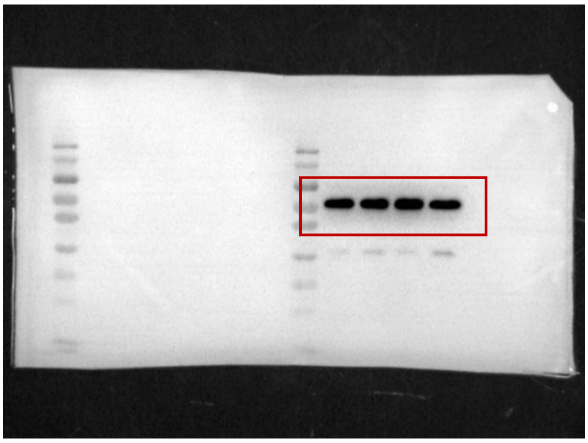

4.Input: Flag

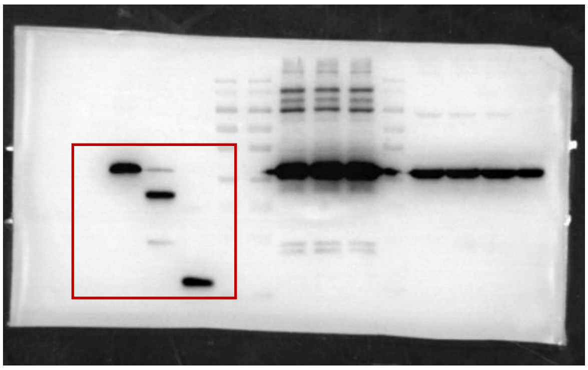

5. Input:β-actin

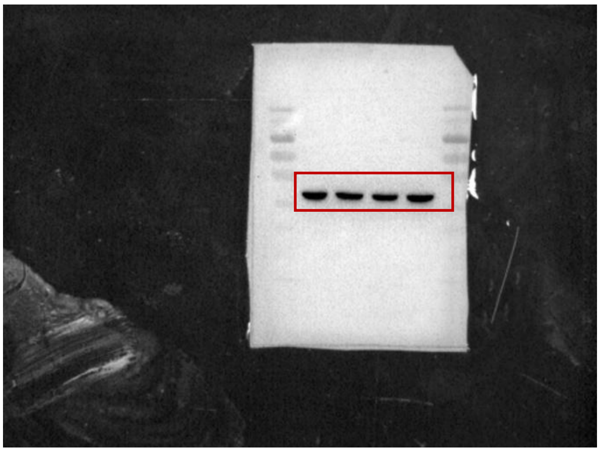

Figure 3 J

1.IP:HA-Ub

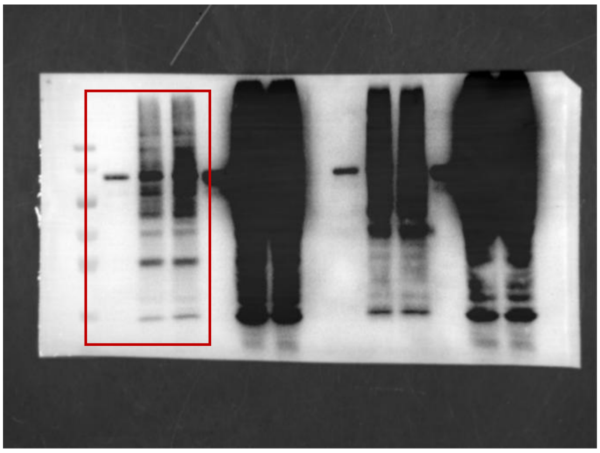

2.IP:Flag-STING

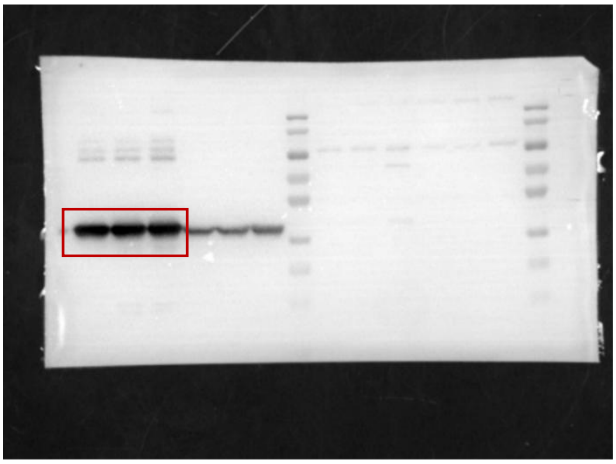

3. Input:HA-Ub

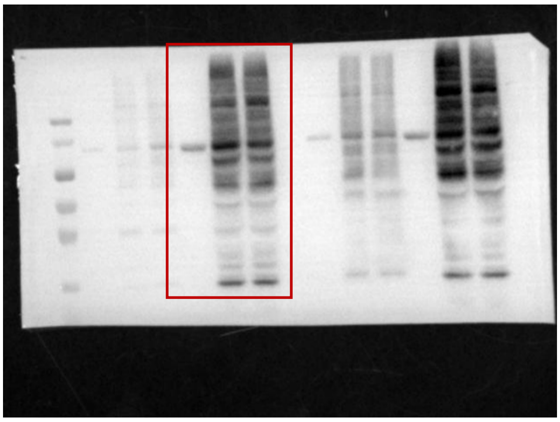

4.Input: Flag-STING

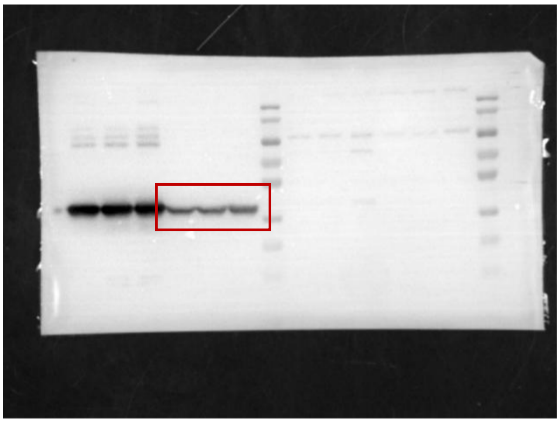

5.Input: Myc-DTX2

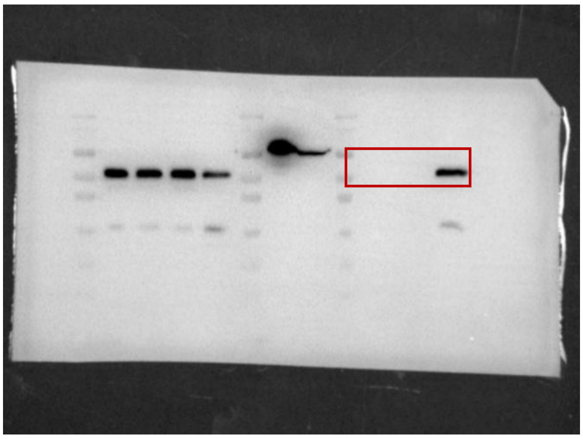

6.Input:β-actin

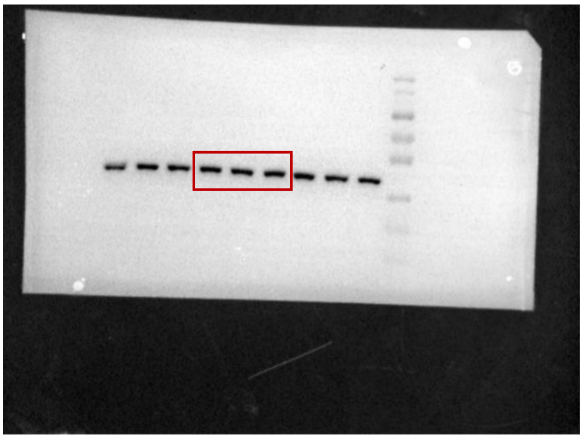

Figure 3 K

1.IP:HA-Ub

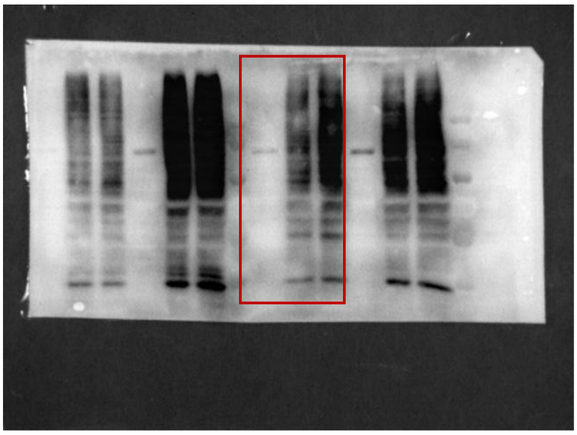

2.IP:Flag-STING

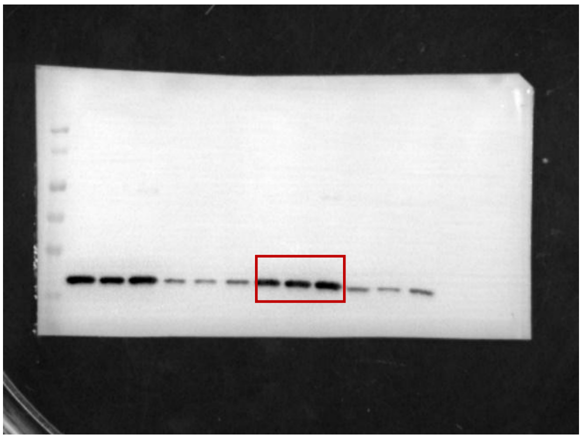

3. Input:HA-Ub

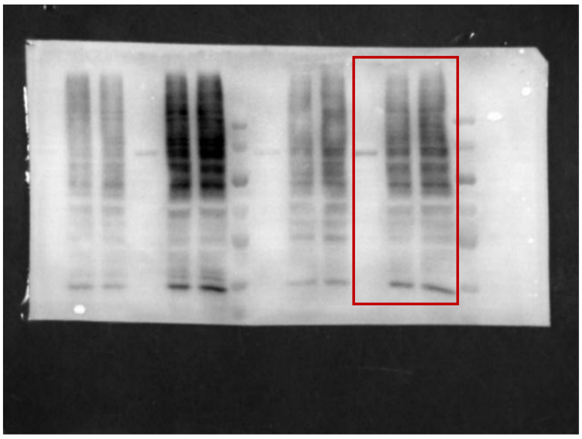

4.Input: Flag-STING

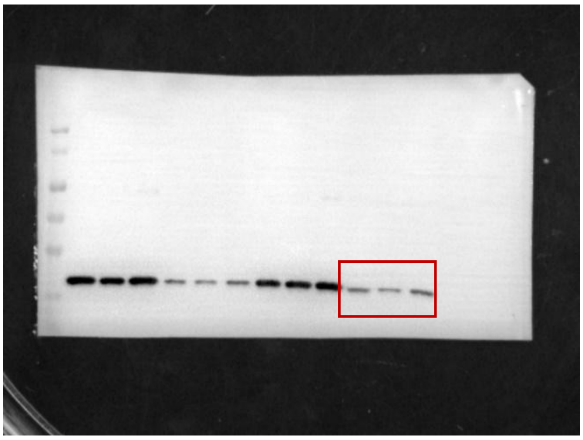

5.Input: Myc-DTX2

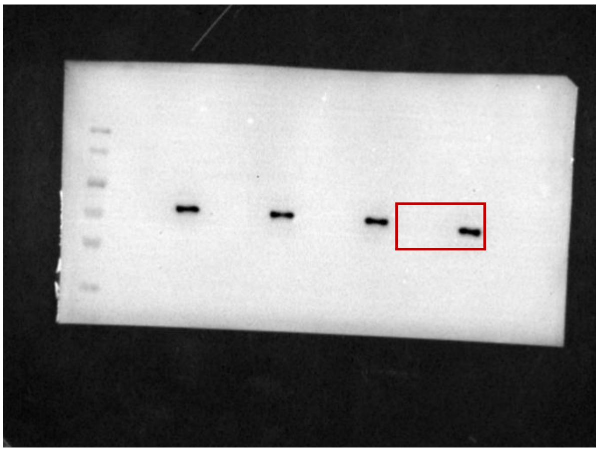

6.Input:β-actin

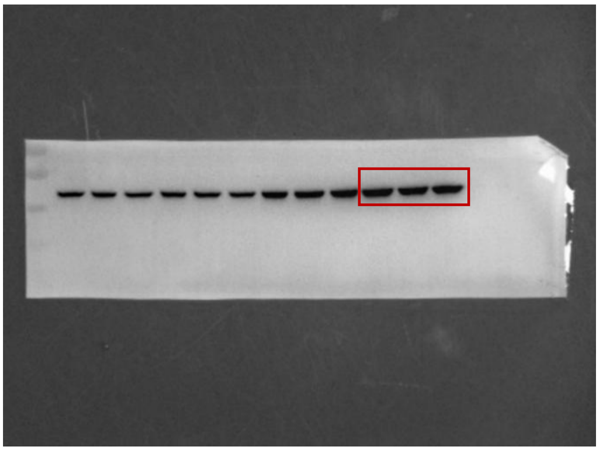

Figure 3 L

1. IP:Ub

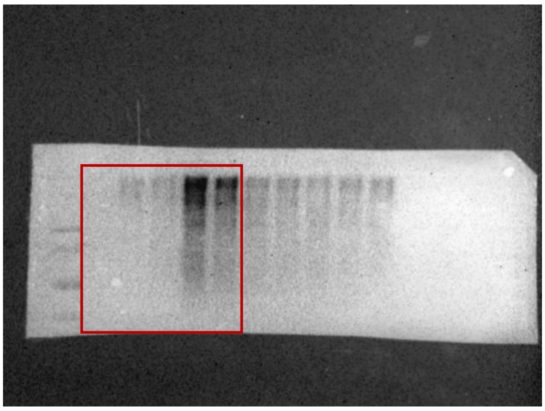

2.IP:Ub-K63

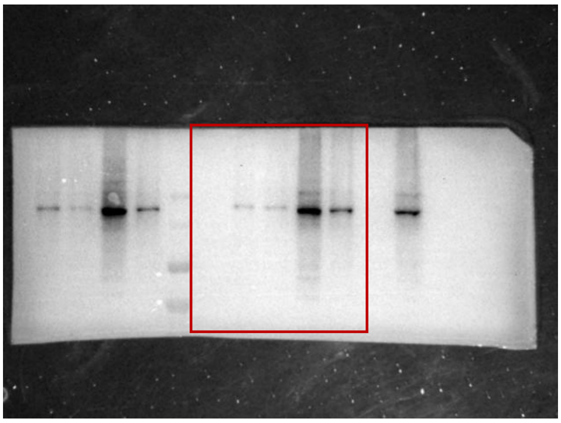

3.IP: STING

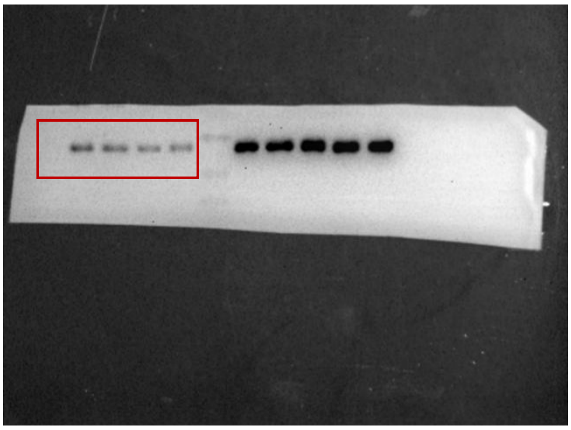

4.Input:Ub

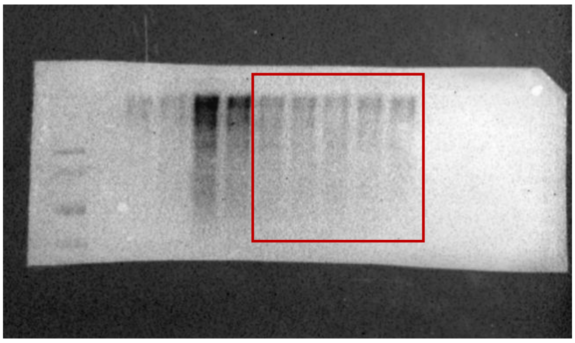

5. Input:Ub-K63

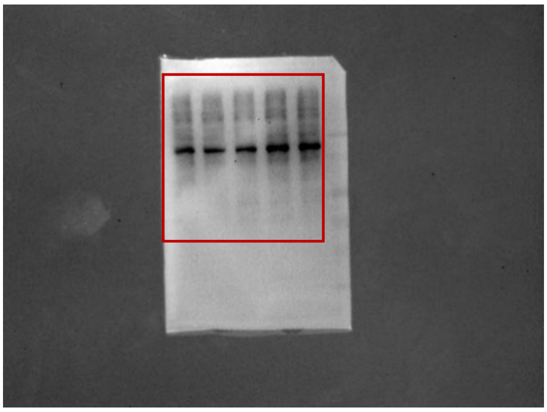

6. Input: STING

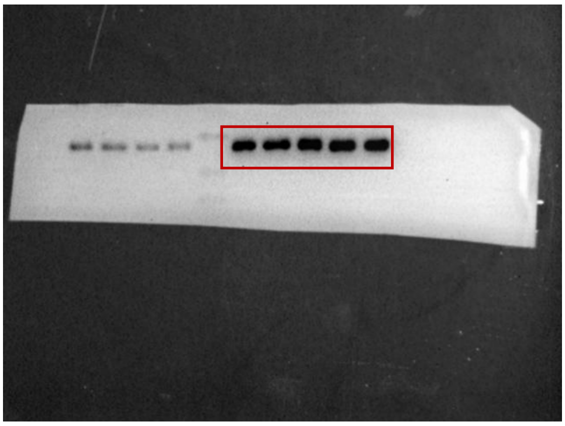

7. Input: Dtx2

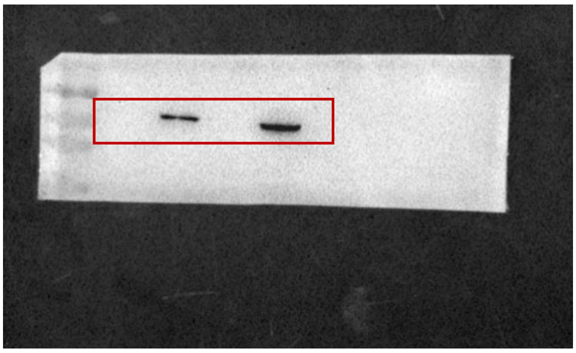

8. Input:β-actin

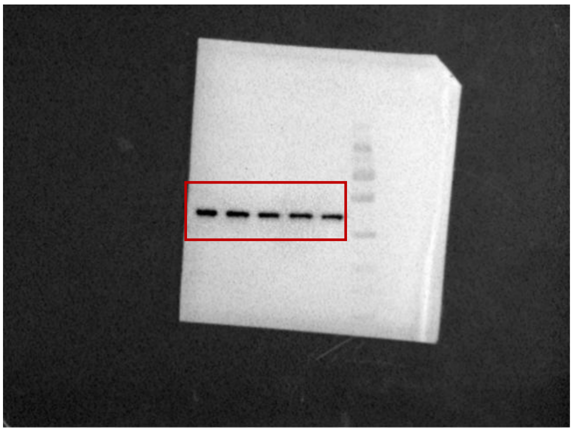

Figure 3 M

1. IP:Ub

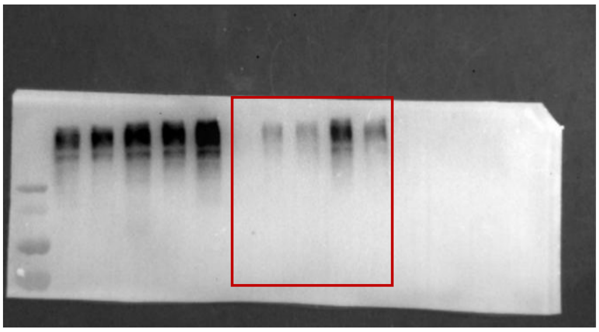

2.IP:Ub-K63

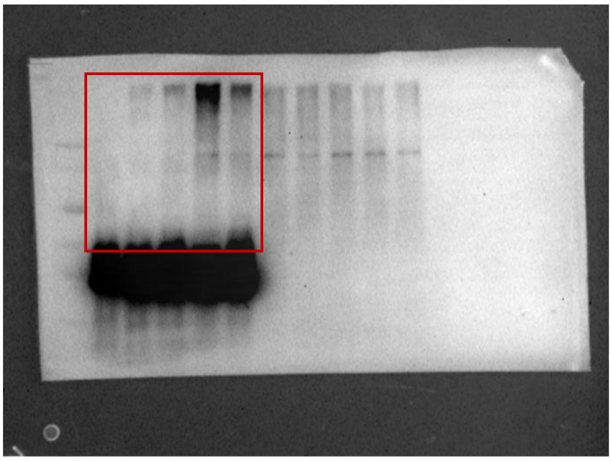

3.IP: STING

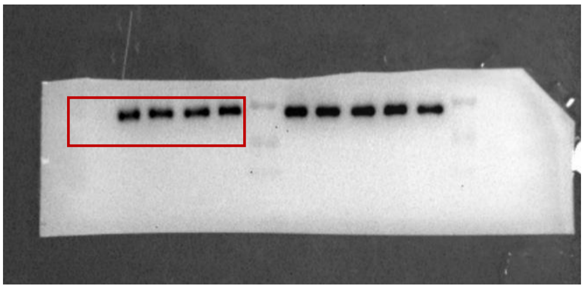

4.Input:Ub

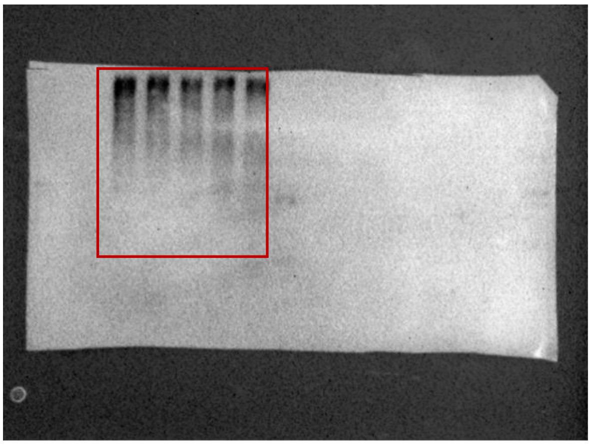

5. Input:Ub-K63

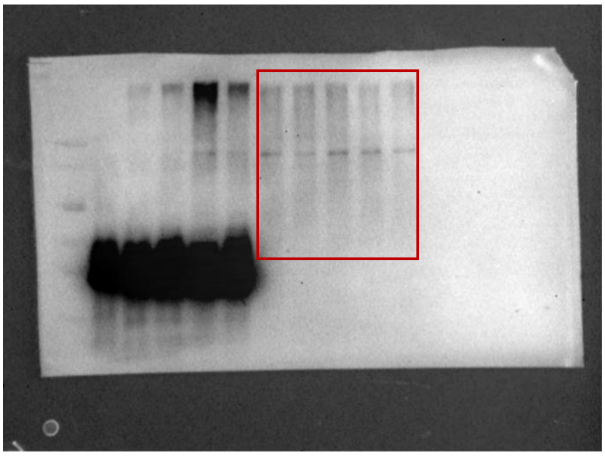

6. Input: STING

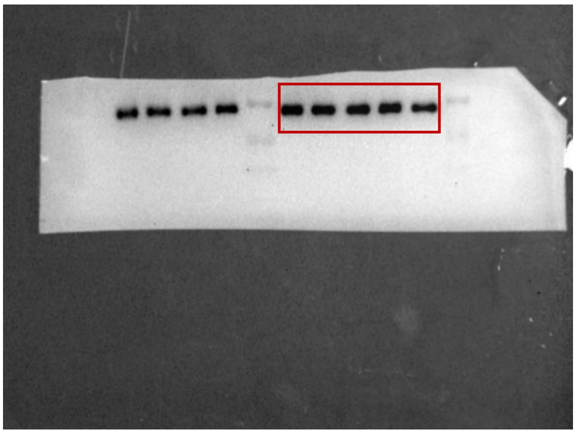

7. Input: Dtx2

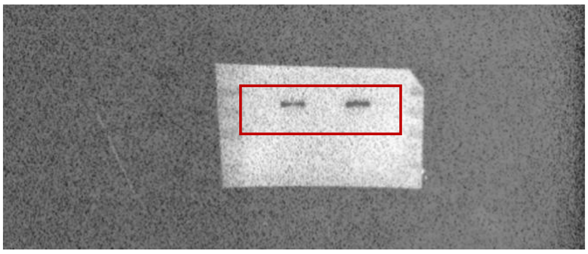

8. Input:β-actin

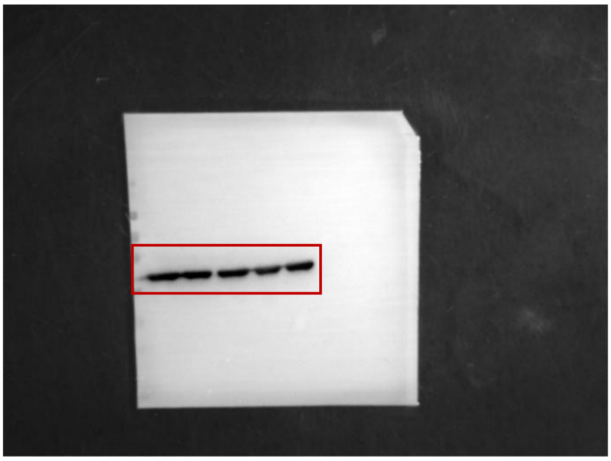

Figure 4 C

1. GM130

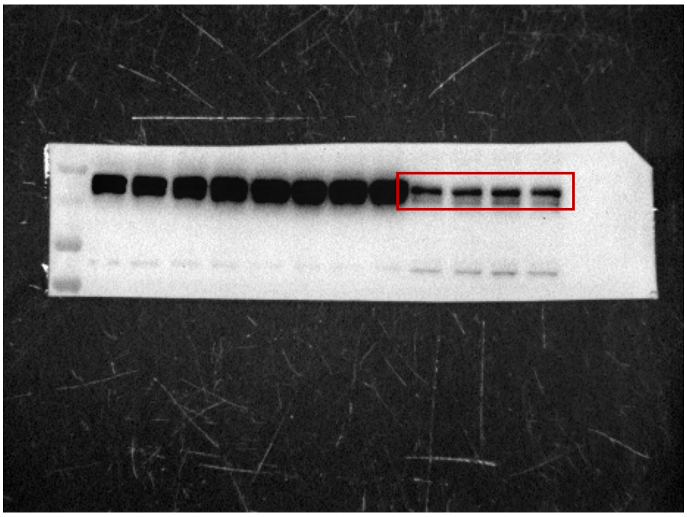

2. ERp72

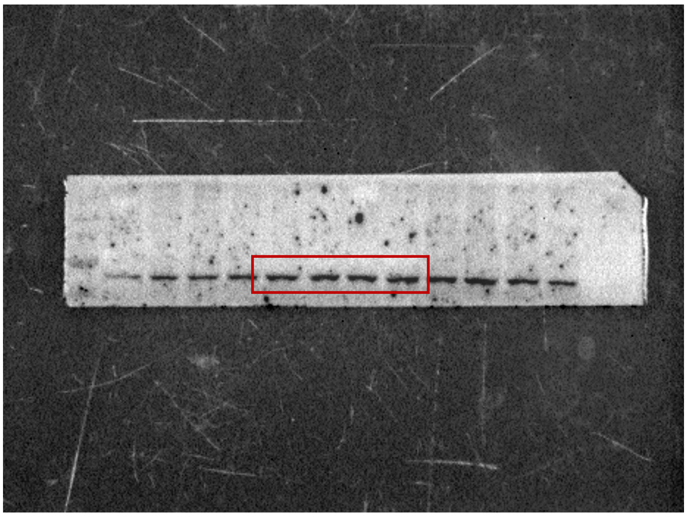

3.β-actin

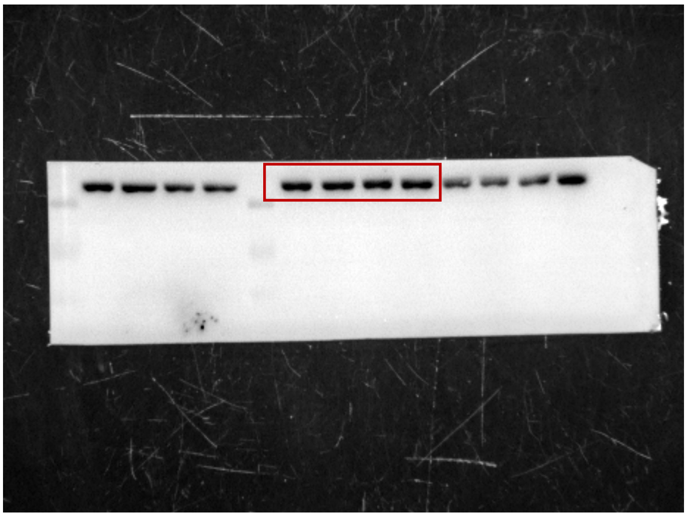

4.STING

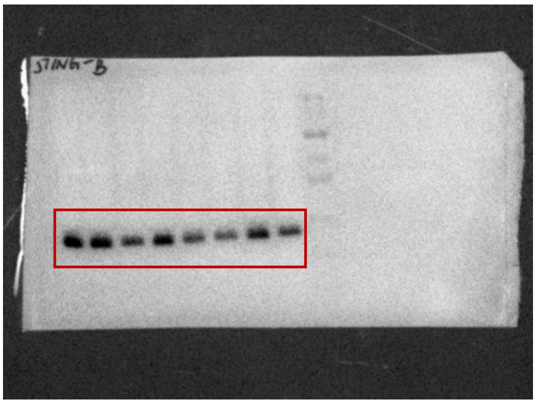

5.GM130

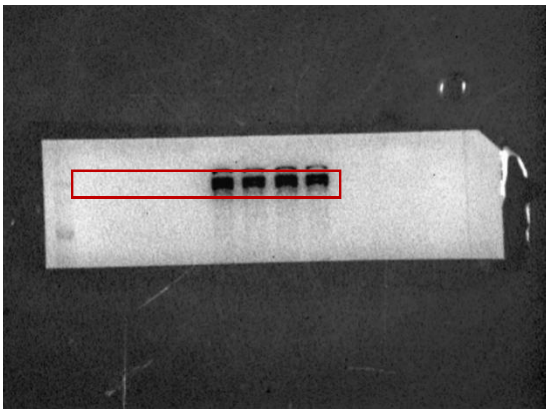

6.ERp72

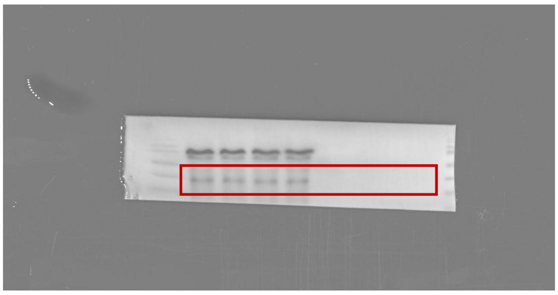

Figure 4 D

1. GM130

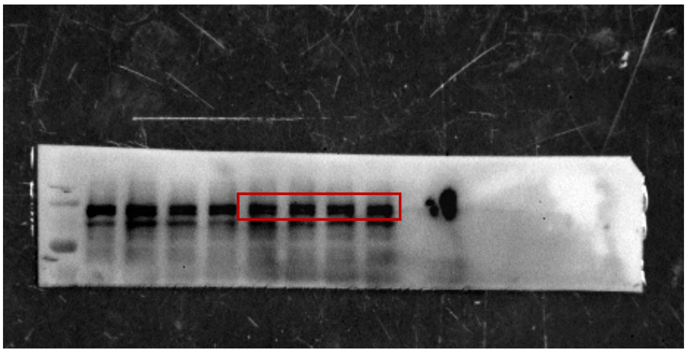

2.ERp72

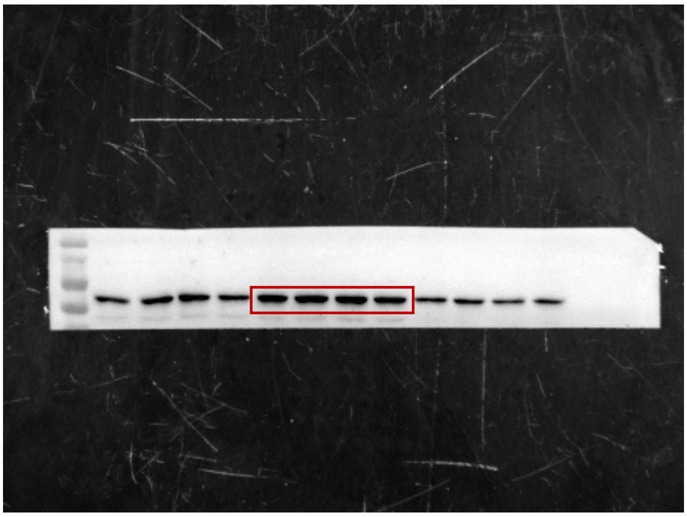

3.β-actin

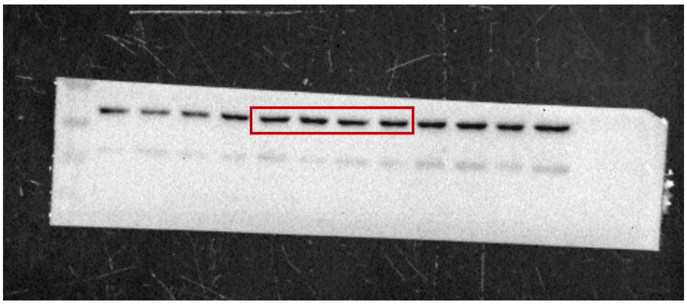

4.STING

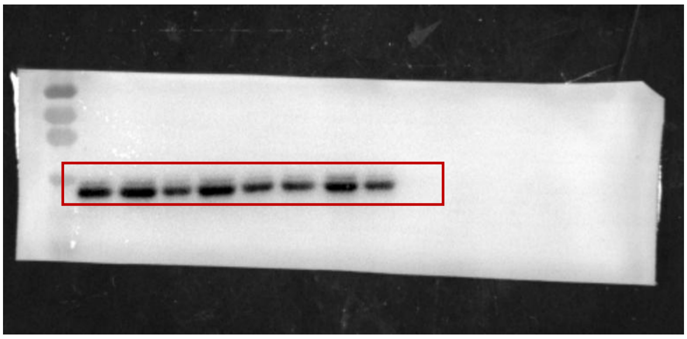

5.GM130

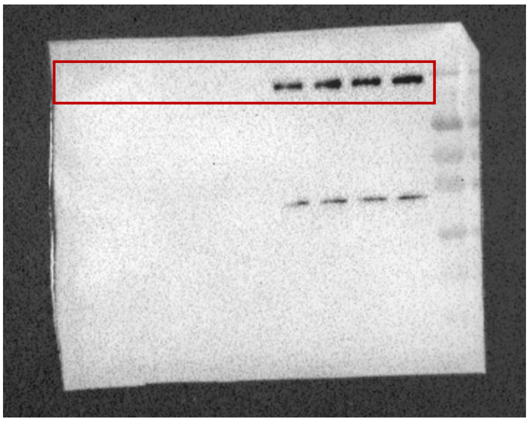

6.ERp72

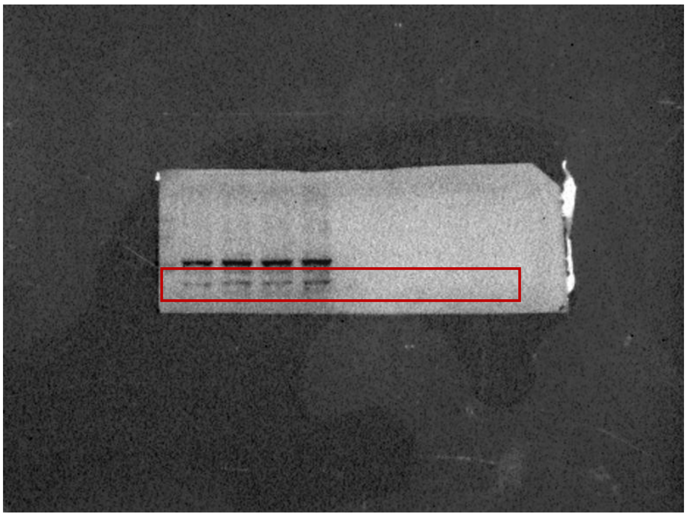

Figure 4 H

1.IP: TBK1

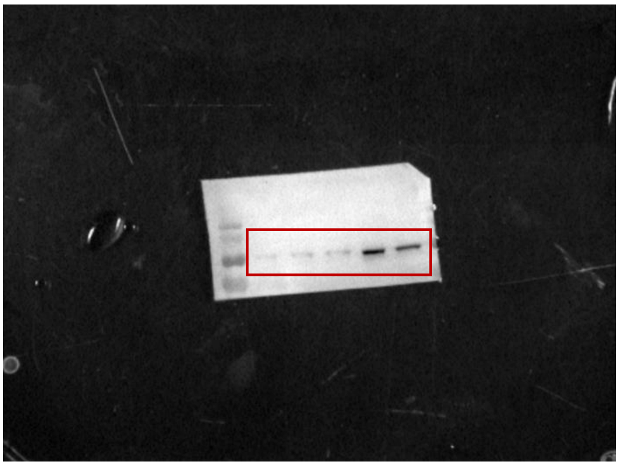

2.IP: STING

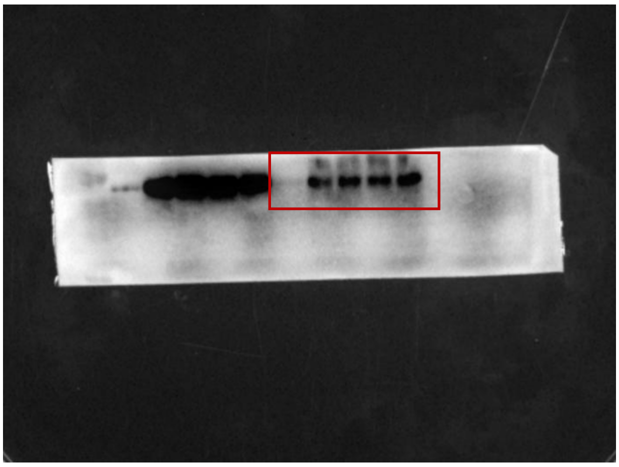

3.Input: TBK1

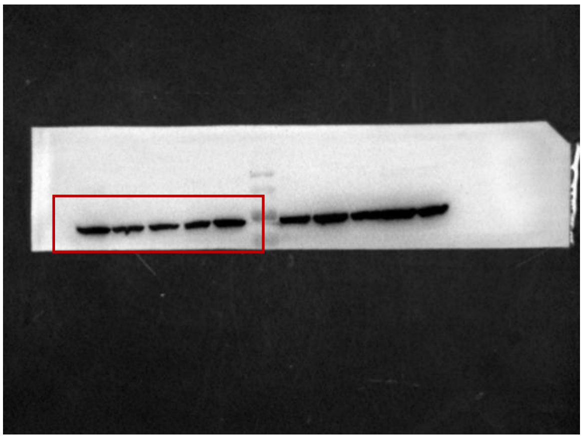

4.Input: STING

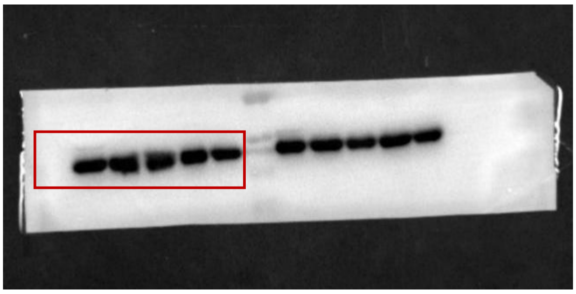

5.Input: Dtx2

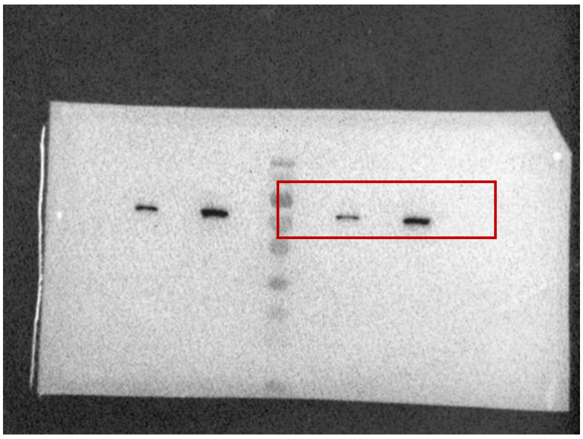

6.Input:β-actin

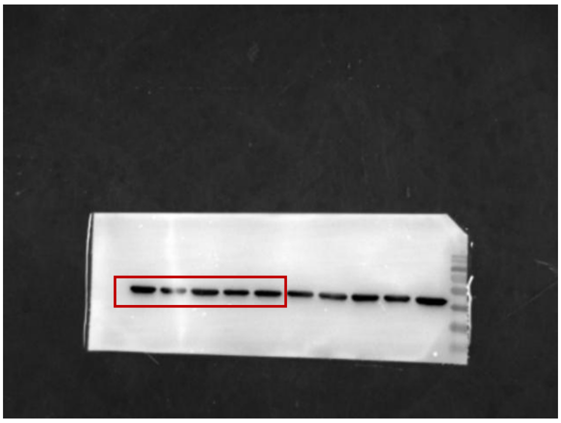

Figure 4 I

1.IP: TBK1

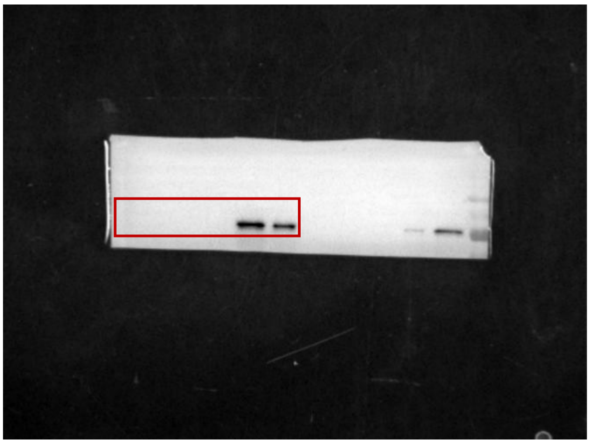

2.IP: STING

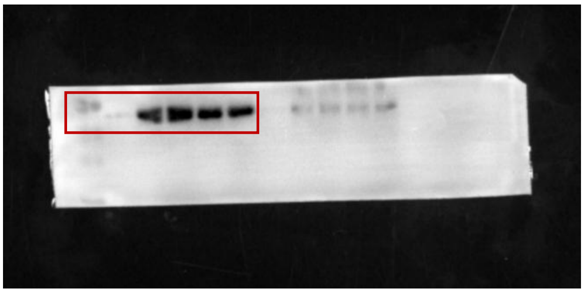

3.Input: TBK1

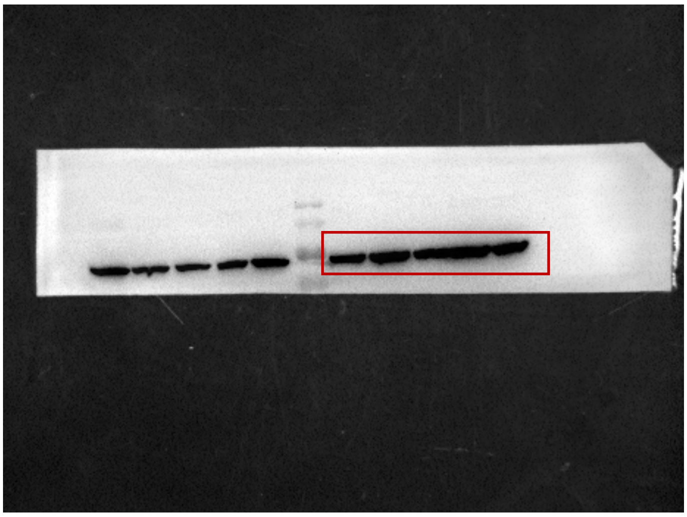

2. Input: STING

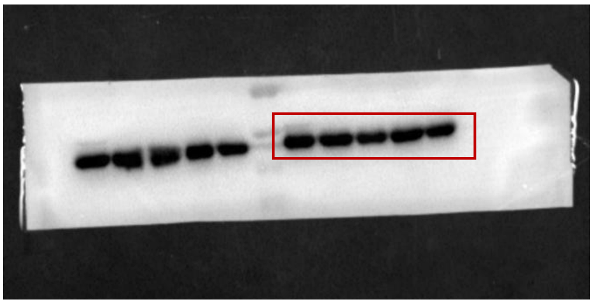

5.Input: Dtx2

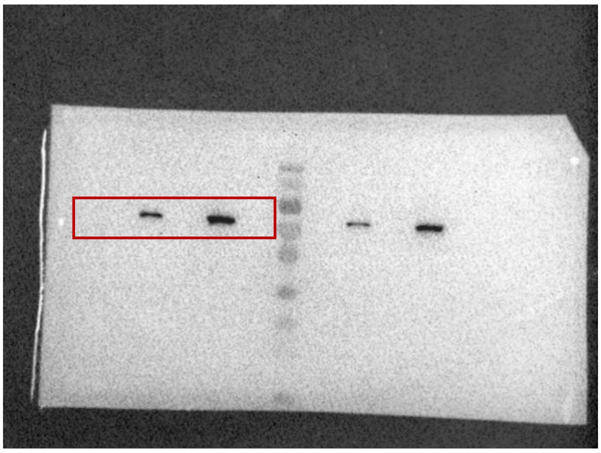

6.Input:β-actin

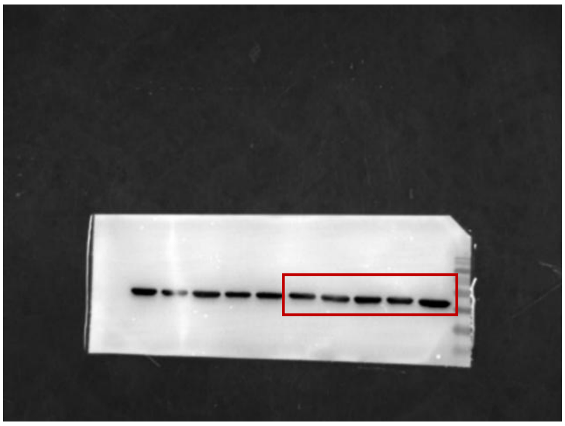

Figure 4 J

1.IP:HA-STING

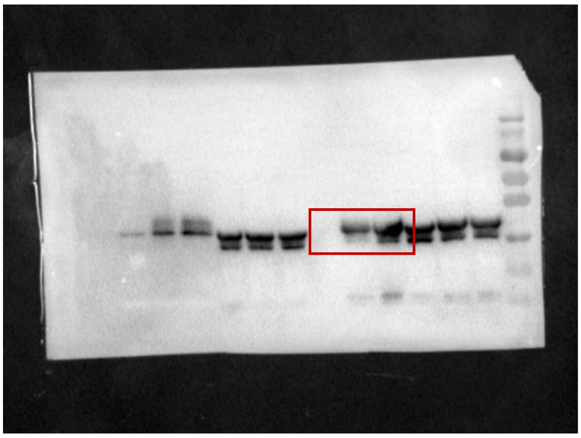

2.IP:Flag-TBK1

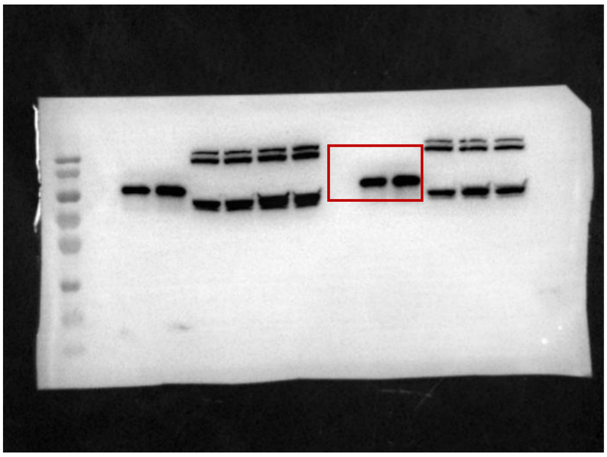

3.Input: HA-STING

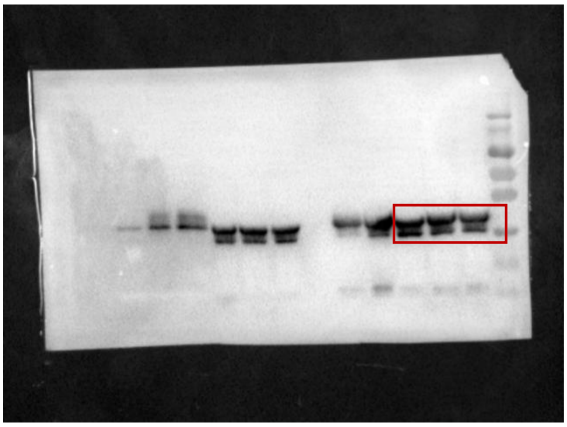

3. Input: Flag-TBK1

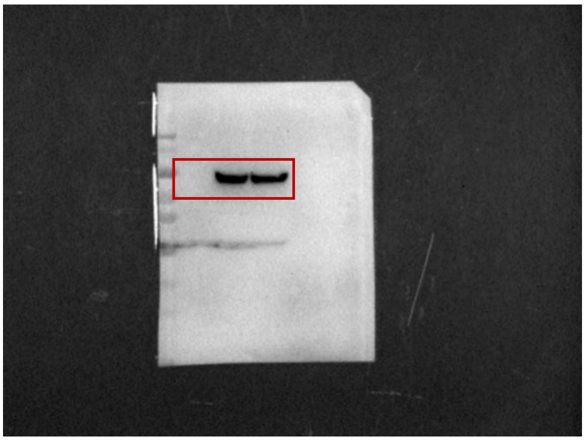

5.Input: Myc-DTX2

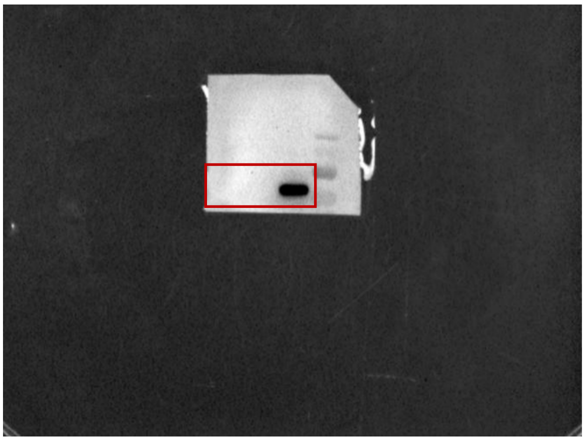

6.Input:β-actin

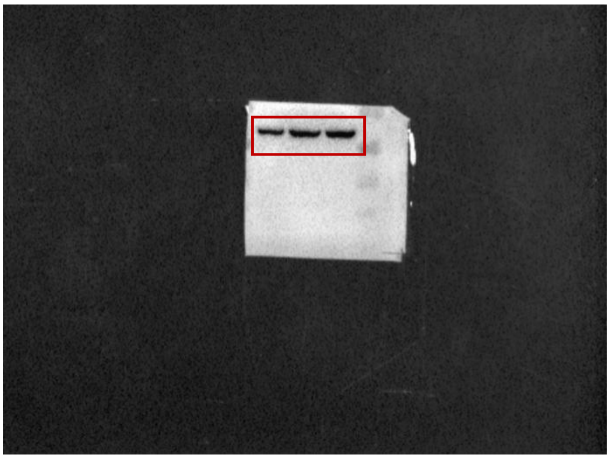

Fig 5 A

1. IP:HA-Ub

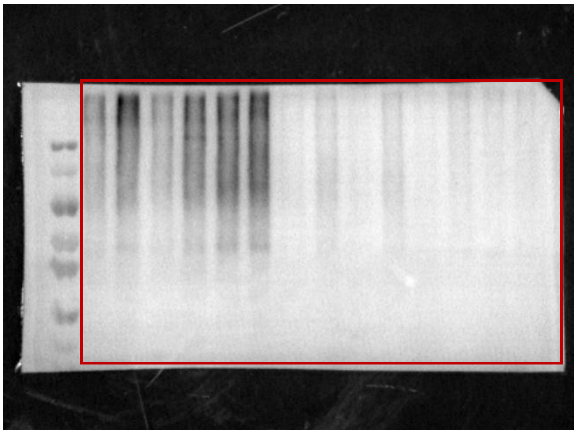

2.IP:Flag-STING

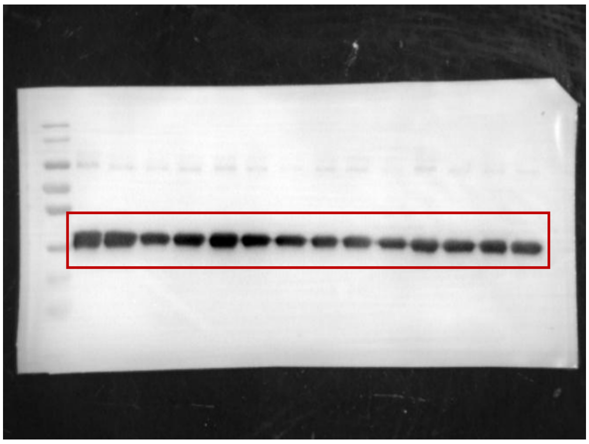

3. Input:HA-Ub

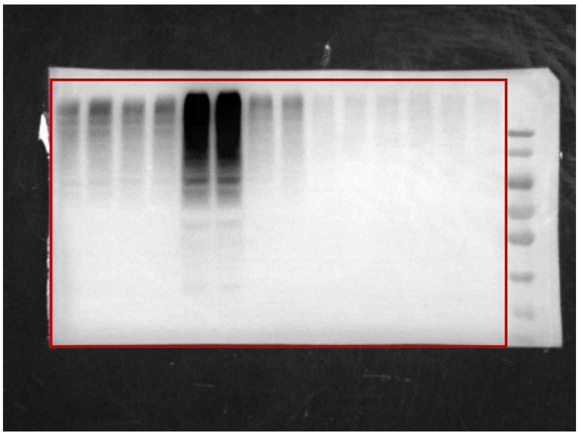

4.Input: Flag-STING

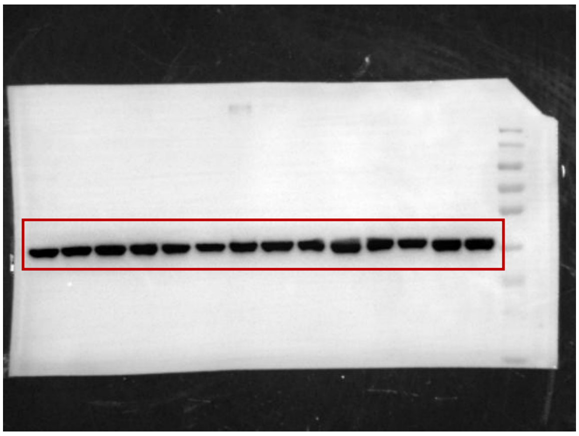

5.Input: Myc-DTX2

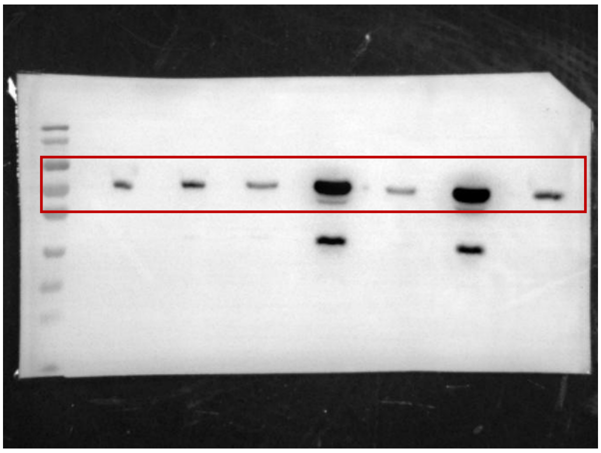

6.Input:β-actin

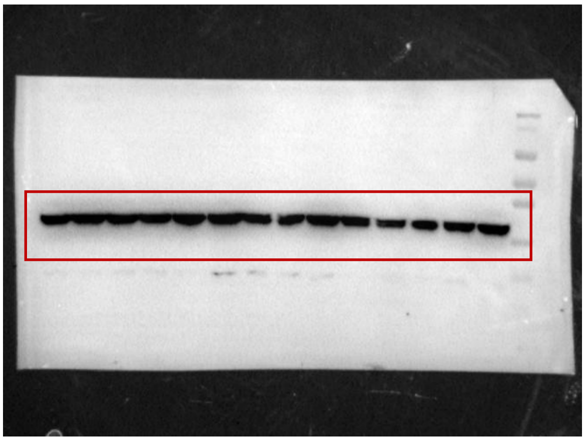

Fig 5 C

1.IP: Myc

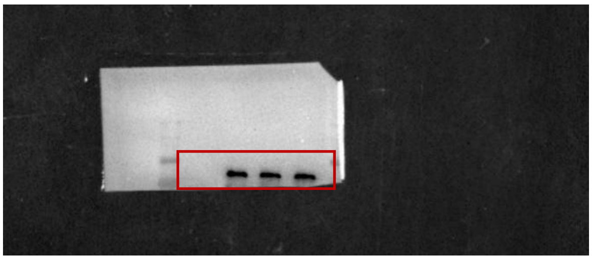

2.IP: Flag

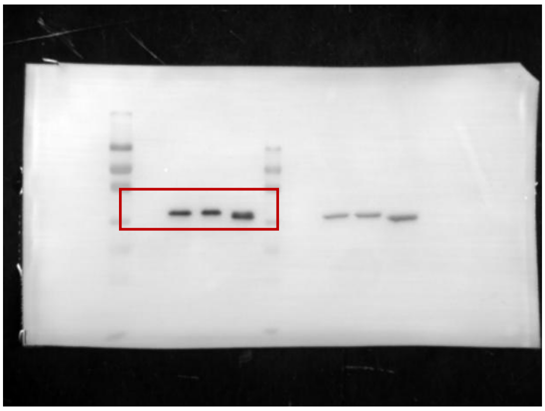

3.Input: Myc

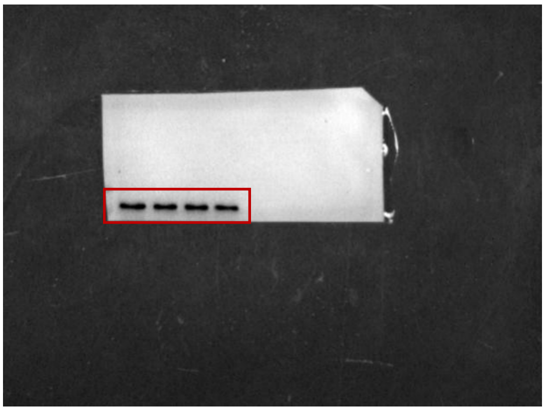

4.Input: Flag

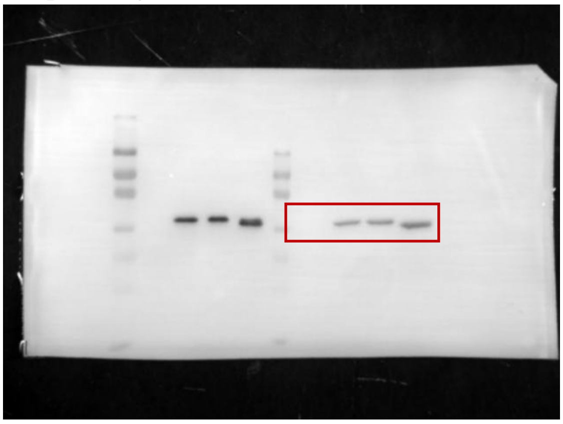

5.Input:β-actin

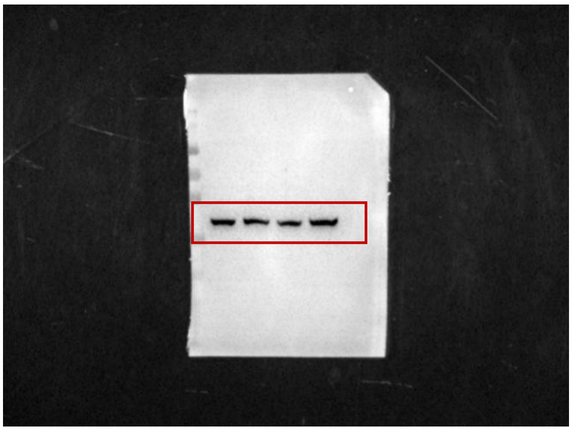

Fig 5 D

1. IP: HA-TBK1

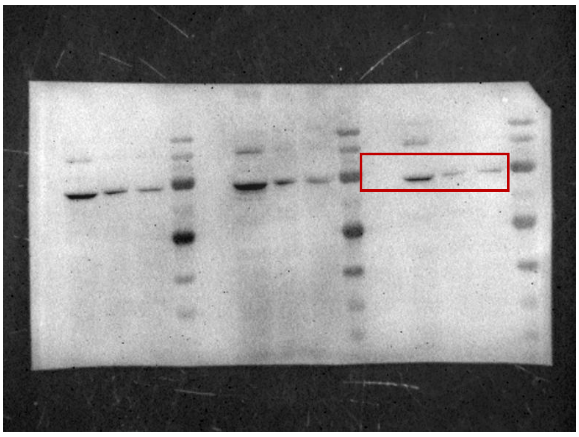

2.IP: Flag-STING

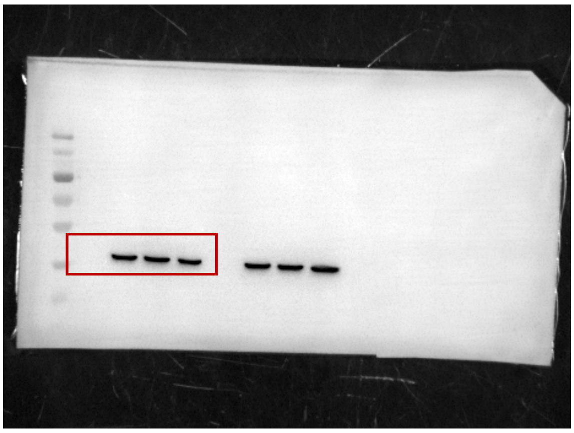

3. Input: HA-TBK1

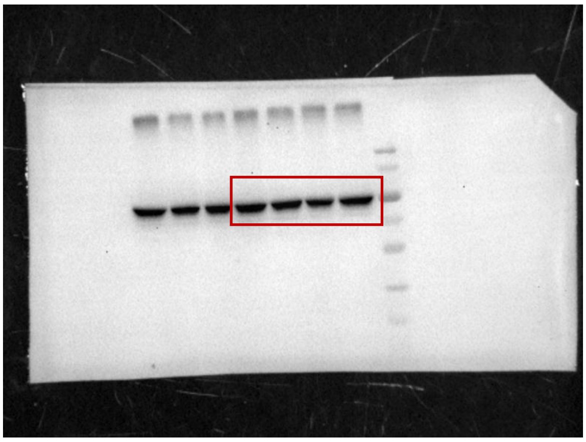

4.Input: Flag-STING

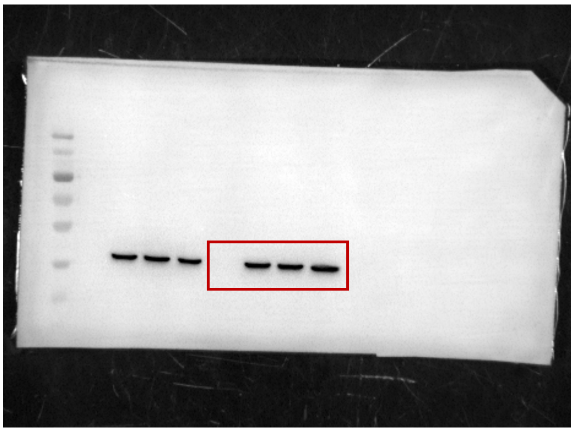

5.Input:β-actin

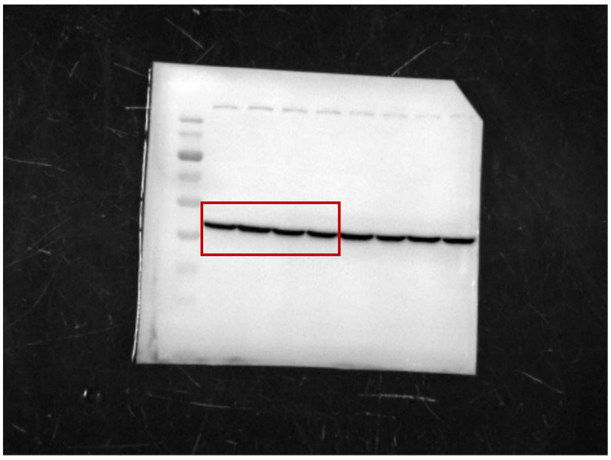

Figure 7 J

1.STING

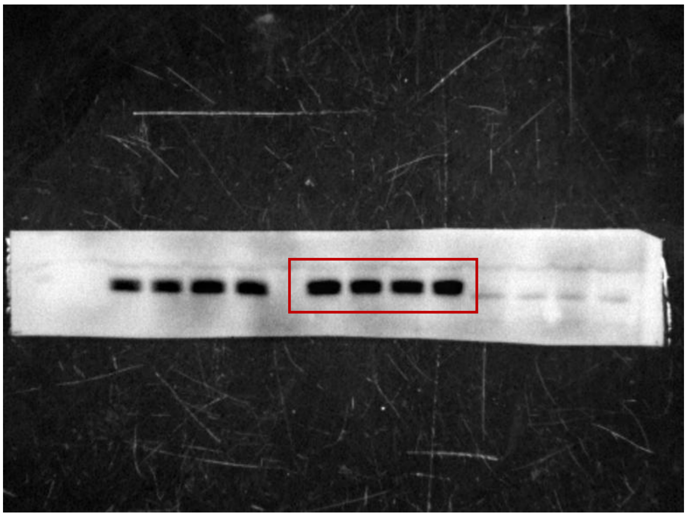

2.p-TBK1

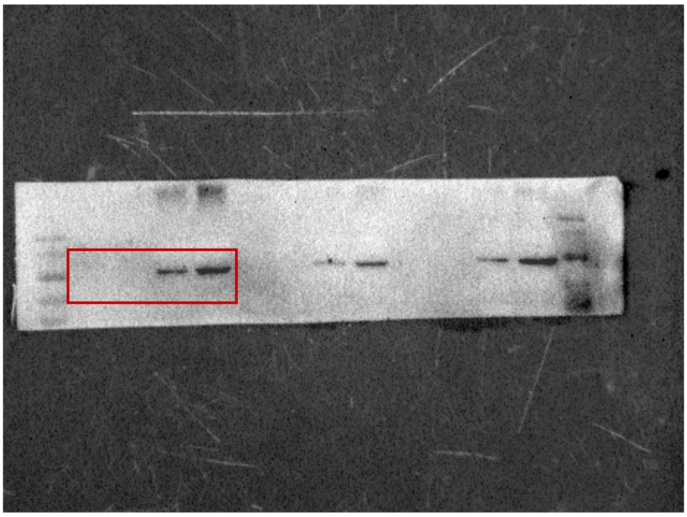

3.TBK1

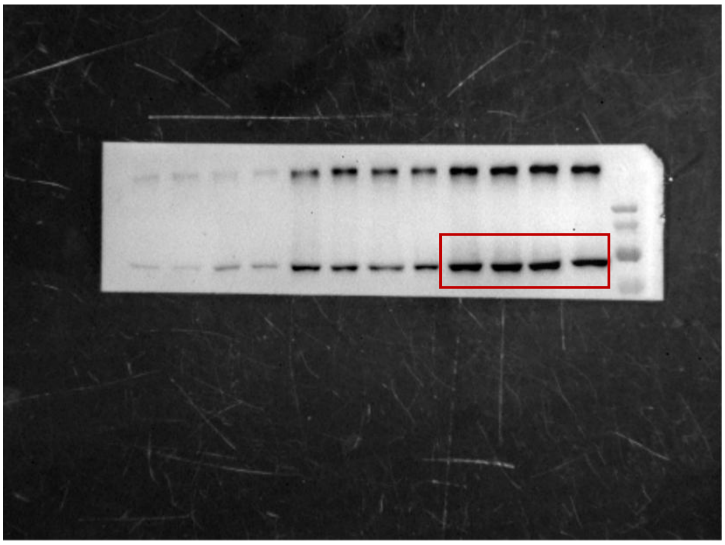

4.p-IRF3

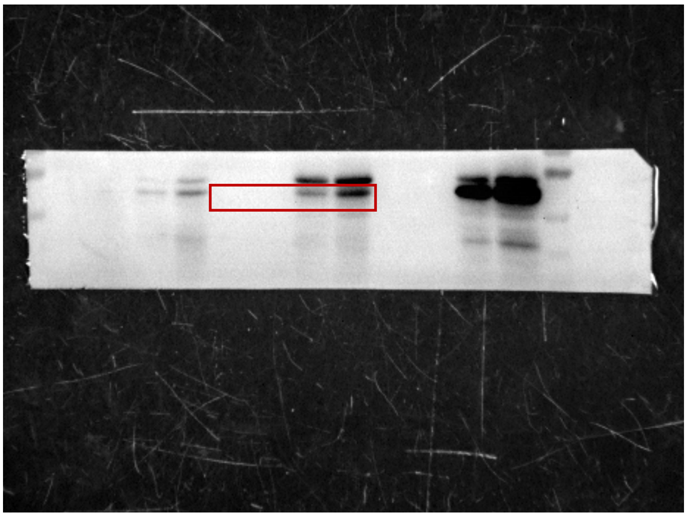

5.IRF3

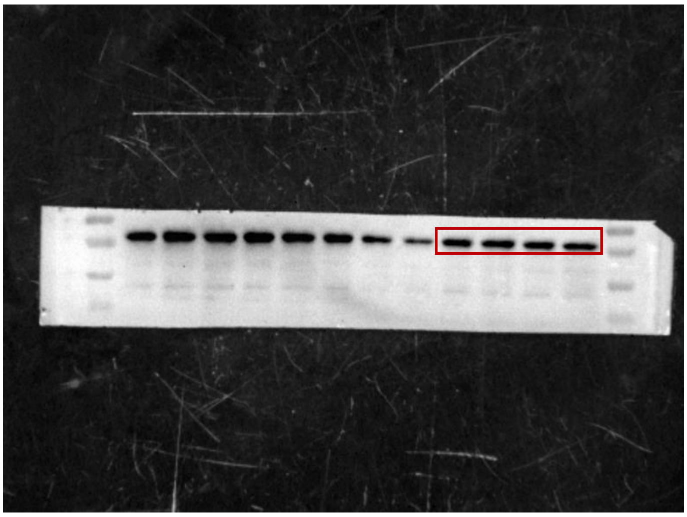

6.β-actin

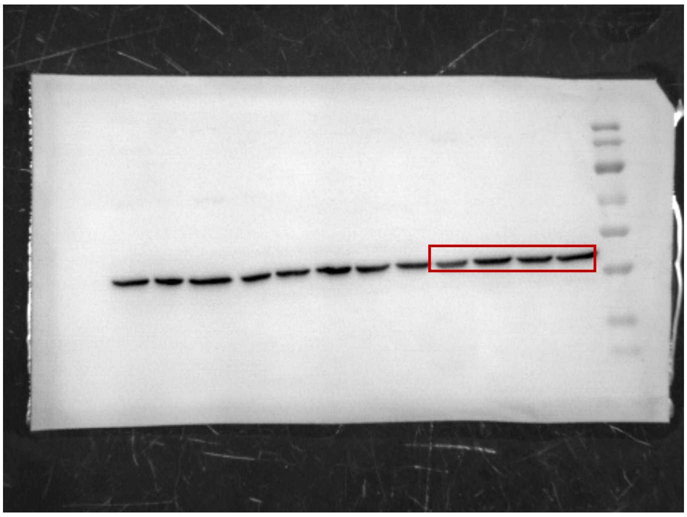

Figure 7 K

1.STING

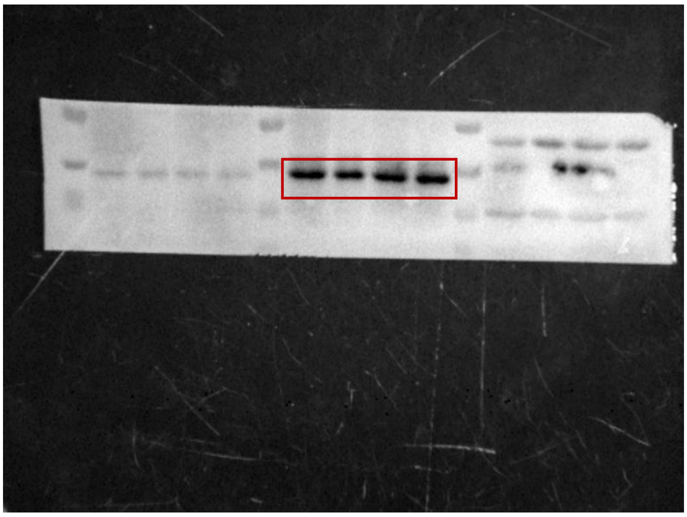

2.p-TBK1

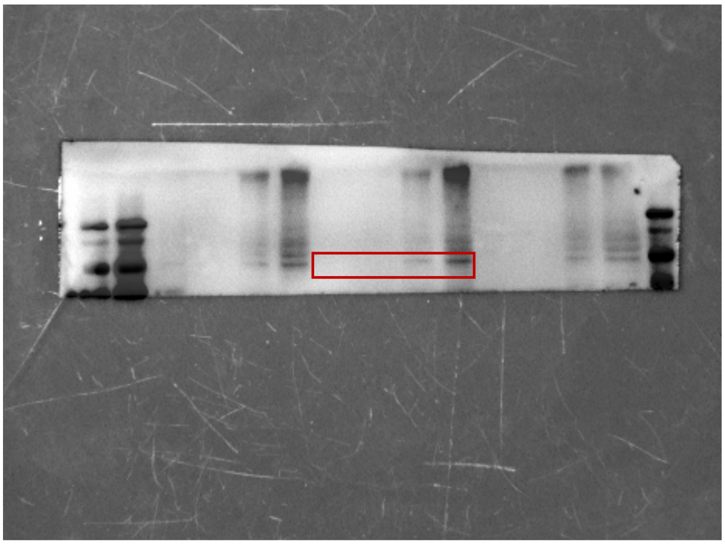

3.TBK1

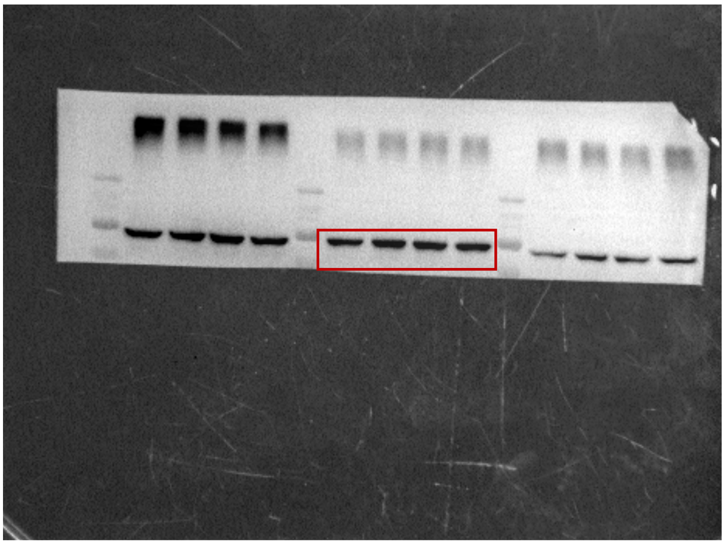

4.p-IRF3

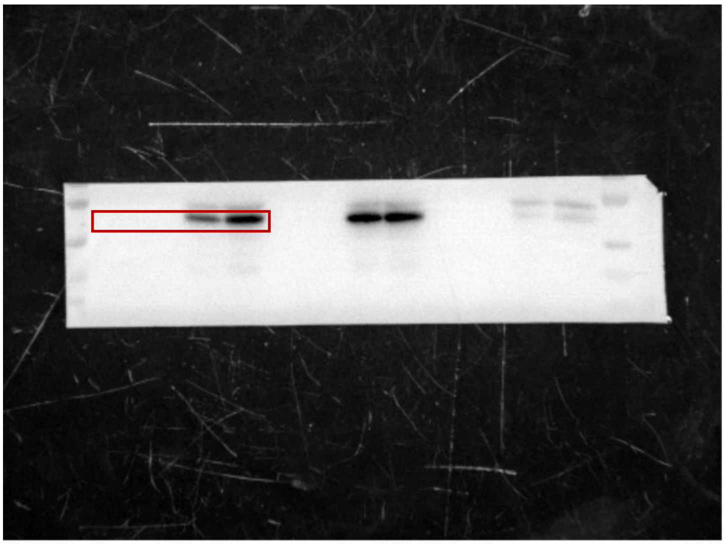

5.IRF3

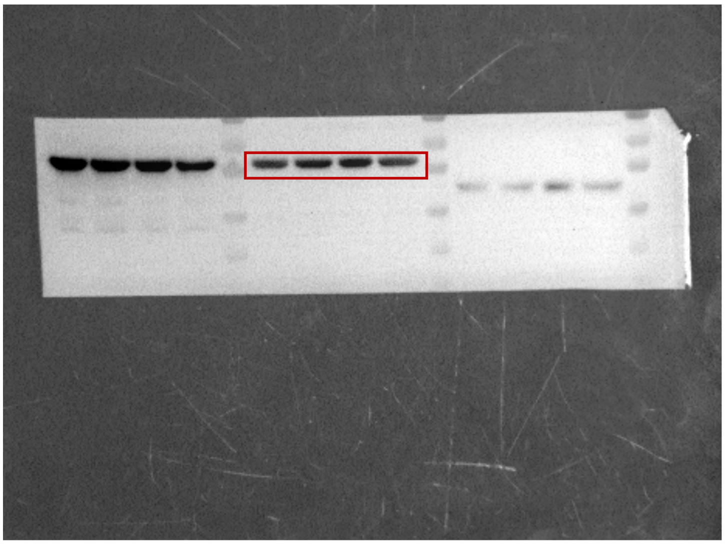

6.β-actin

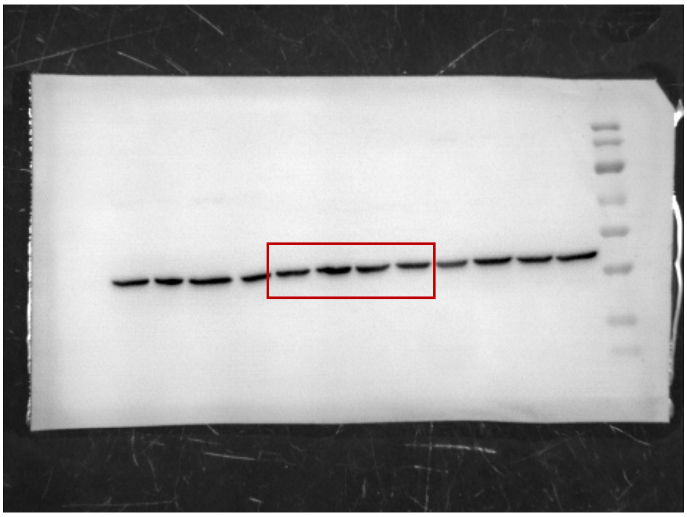

Figure S1 B

1.Dtx2

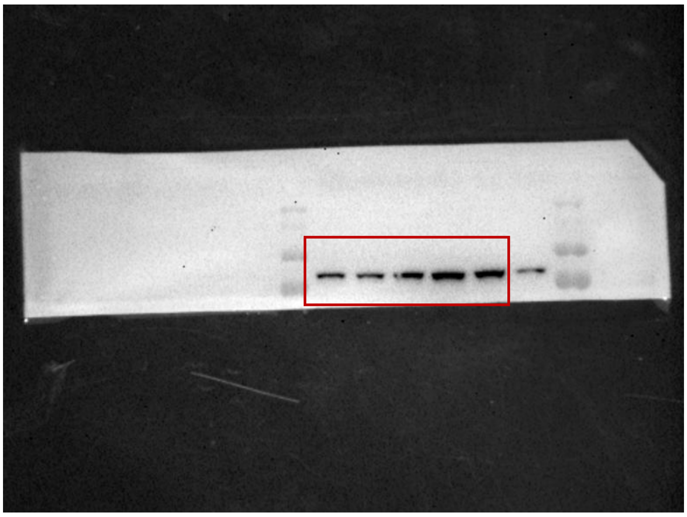

2. β-actin

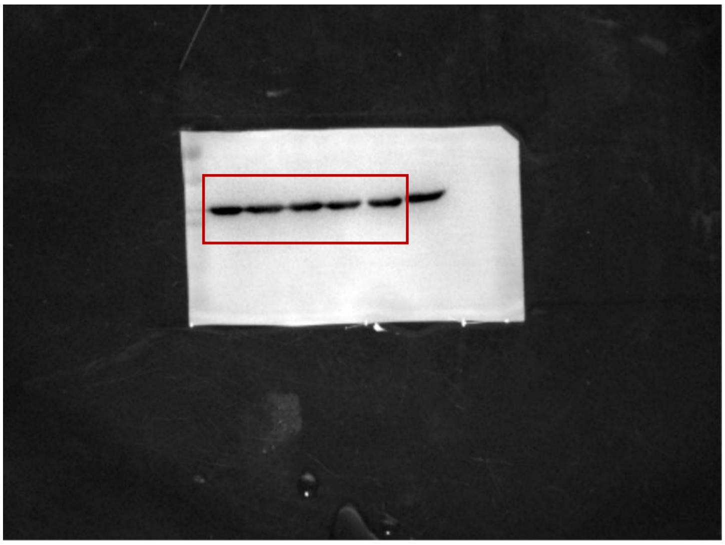

Figure S1 F

1.Dtx2

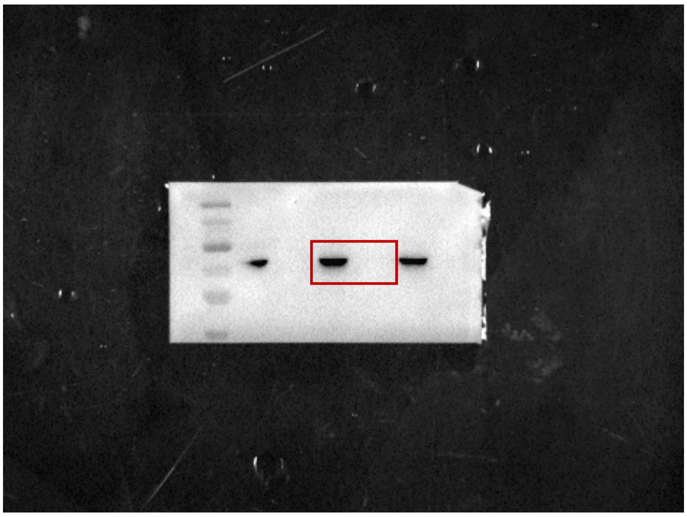

2. β-actin

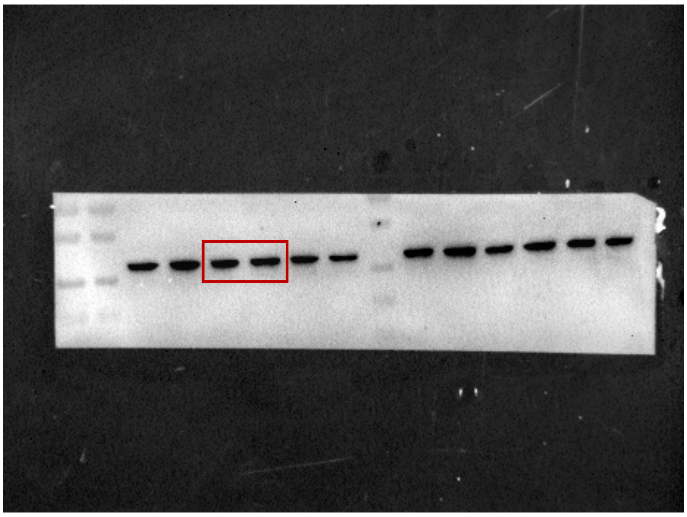

Figure S1 H

1.Dtx2

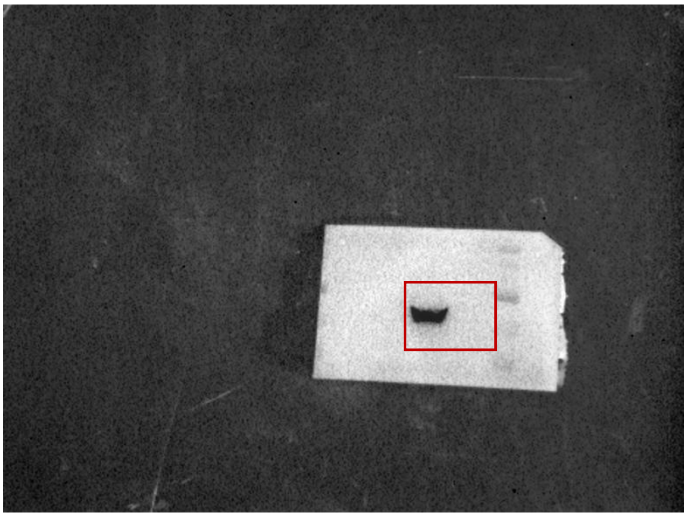

2. β-actin

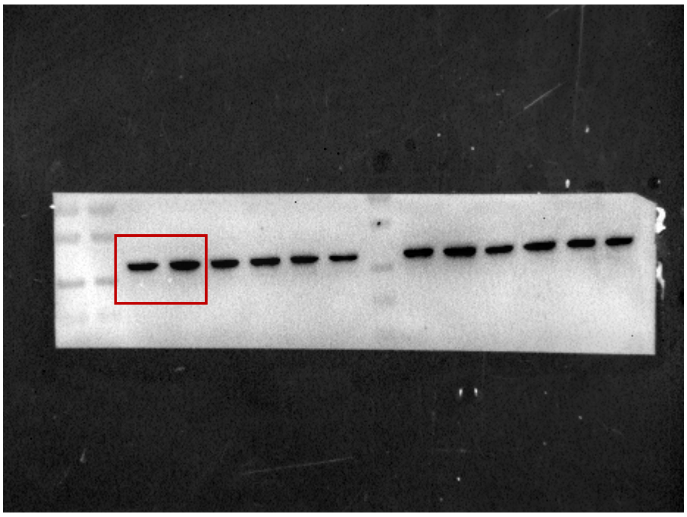

Figure S1 J

1.Dtx2

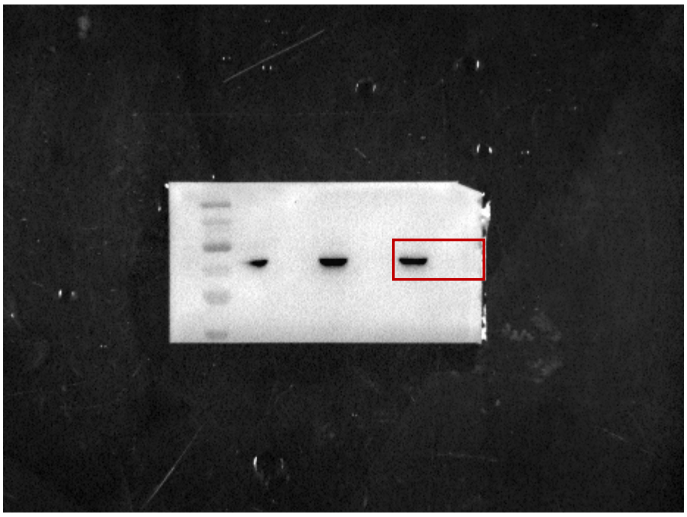

2. β-actin

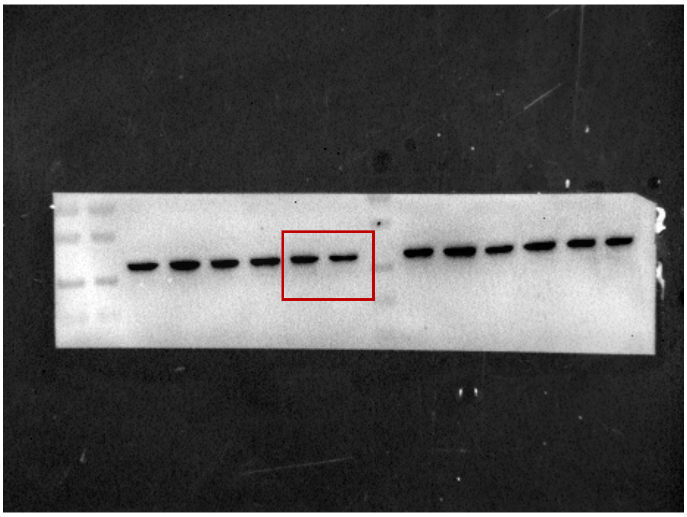

Figure S2 A

1.STING

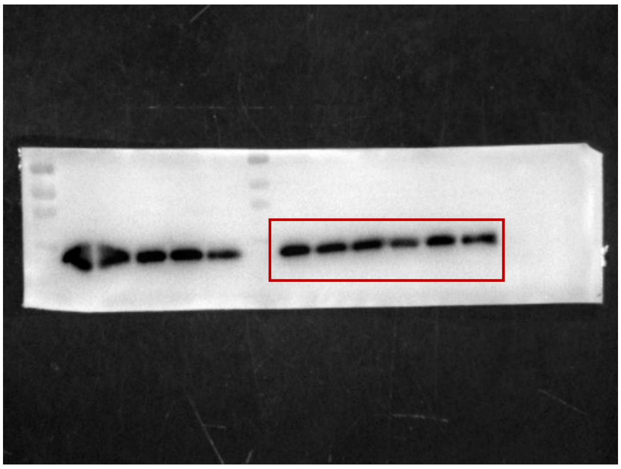

2.p-TBK1

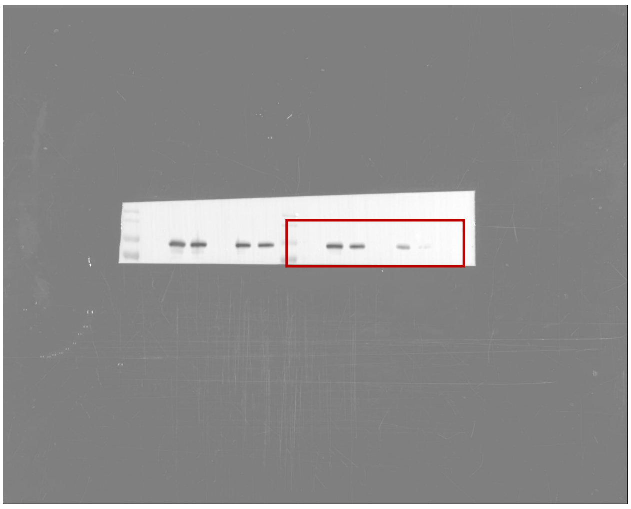

3.TBK1

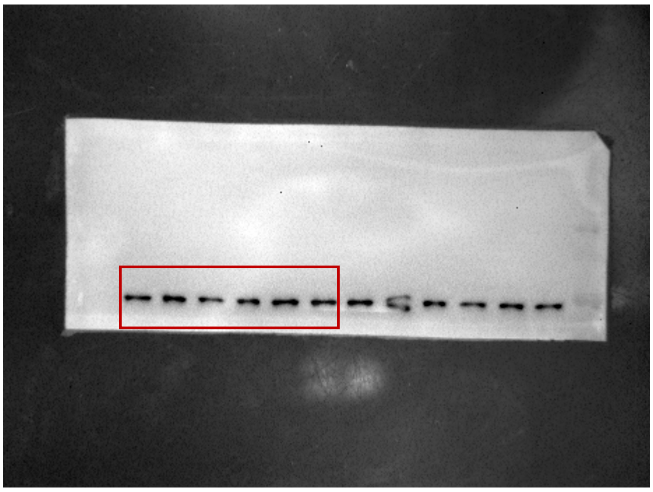

4.p-p65

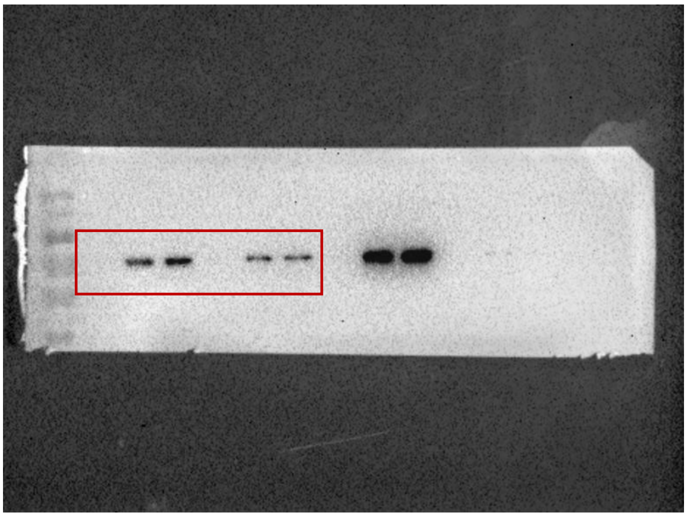

5.p65

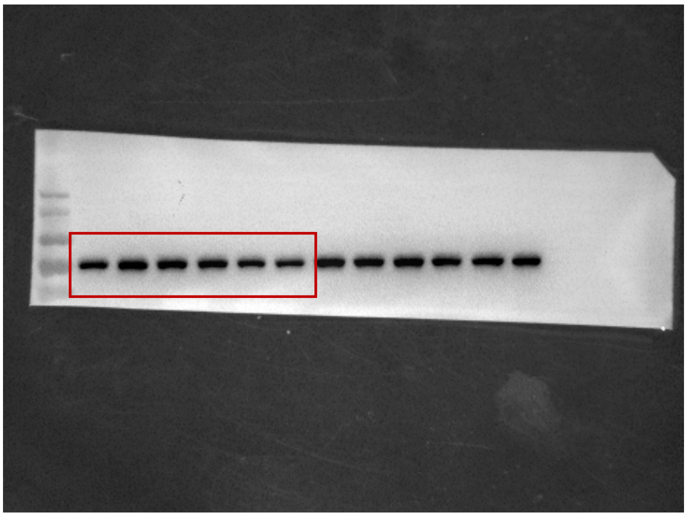

6.p-IRF3

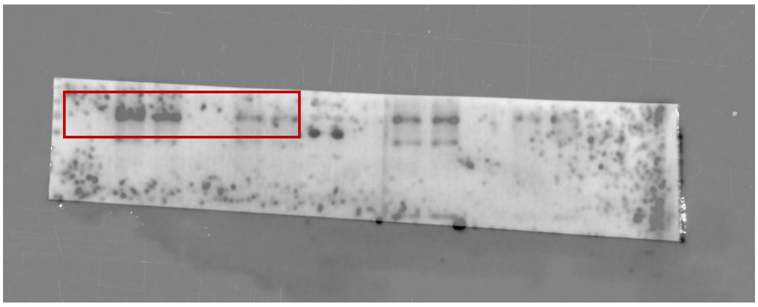

7.IRF3

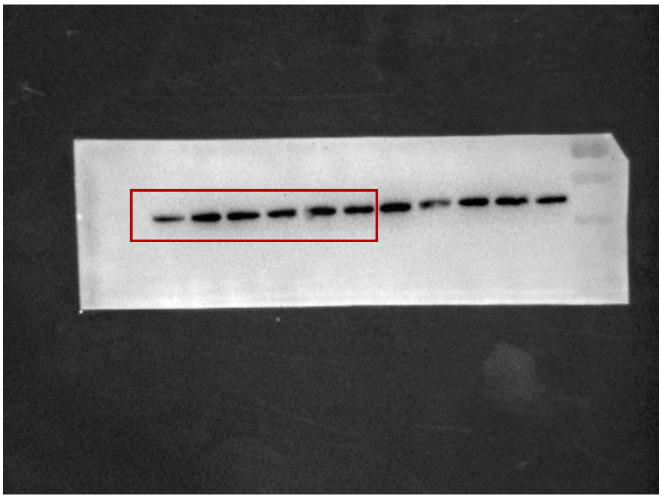

8. β-actin

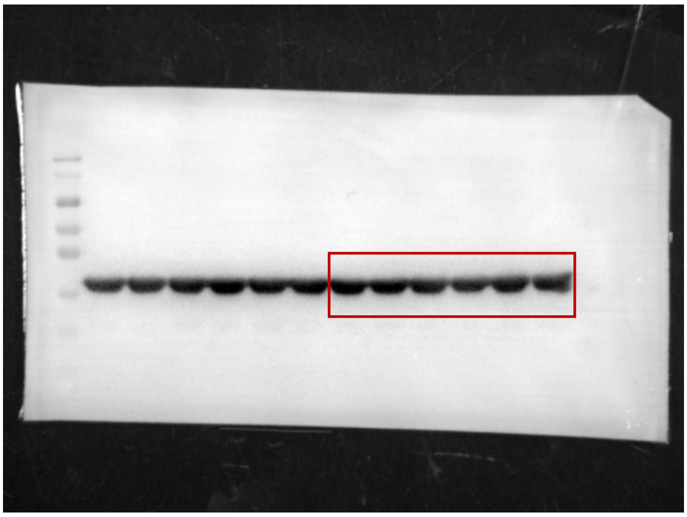

Figure S2 B

1.STING

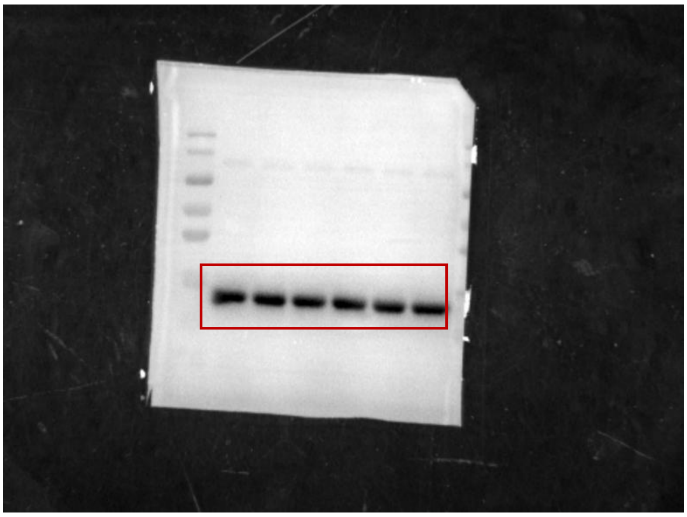

2.p-TBK1

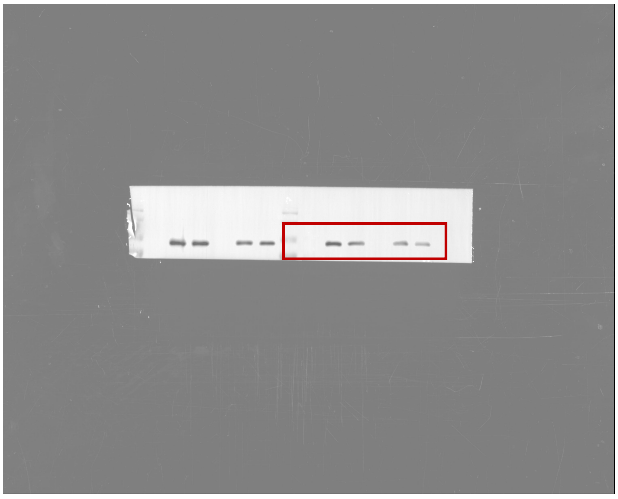

3.TBK1

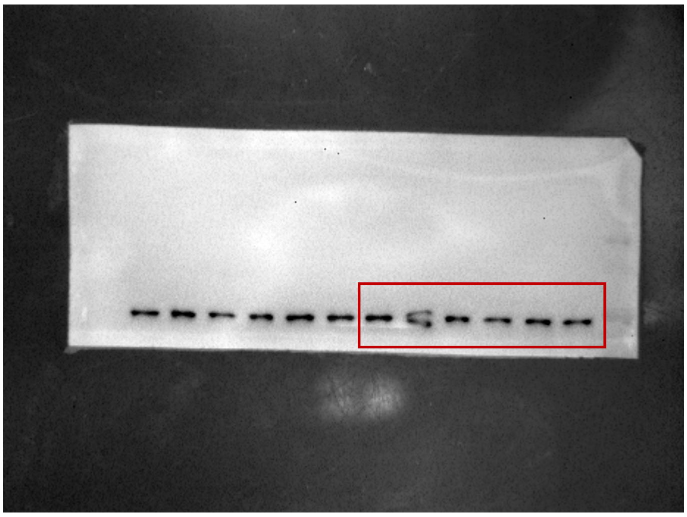

4.p-p65

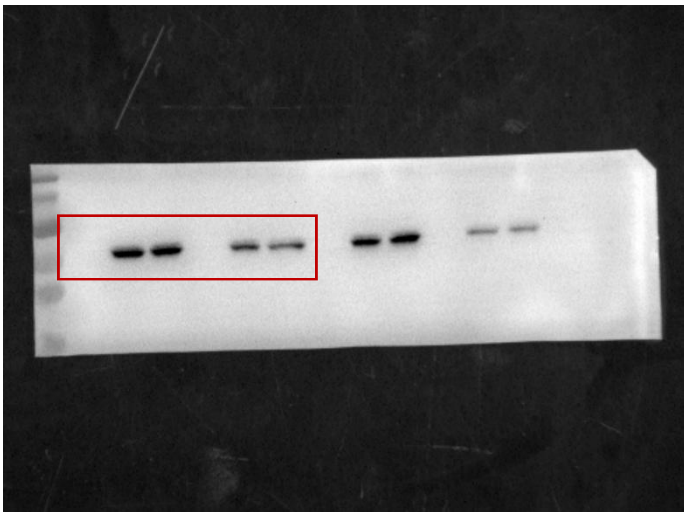

5.p65

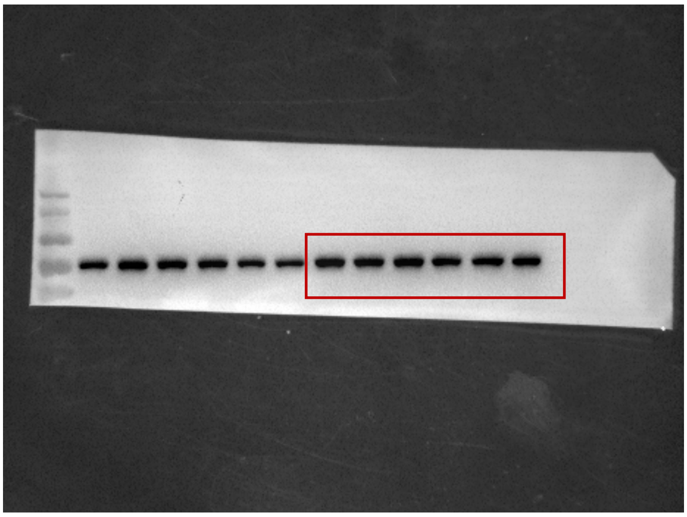

6.p-IRF3

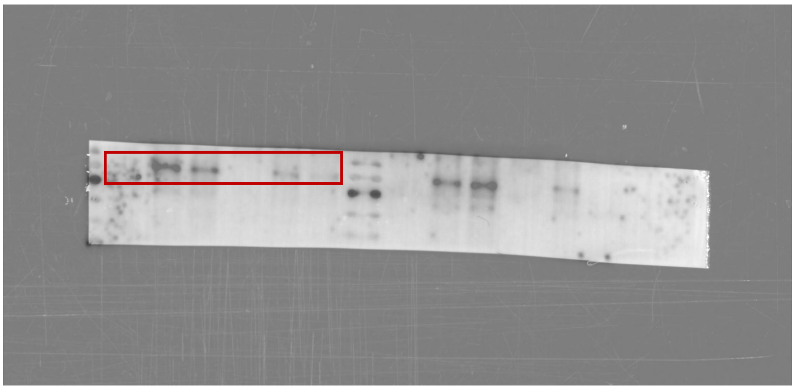

7.IRF3

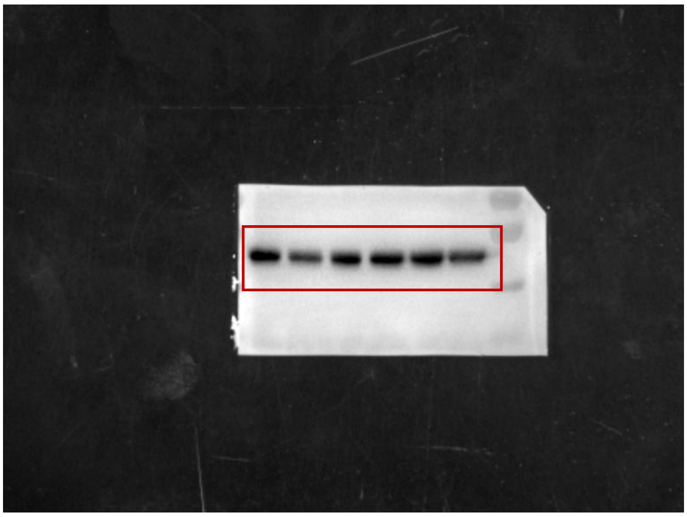

8. β-actin

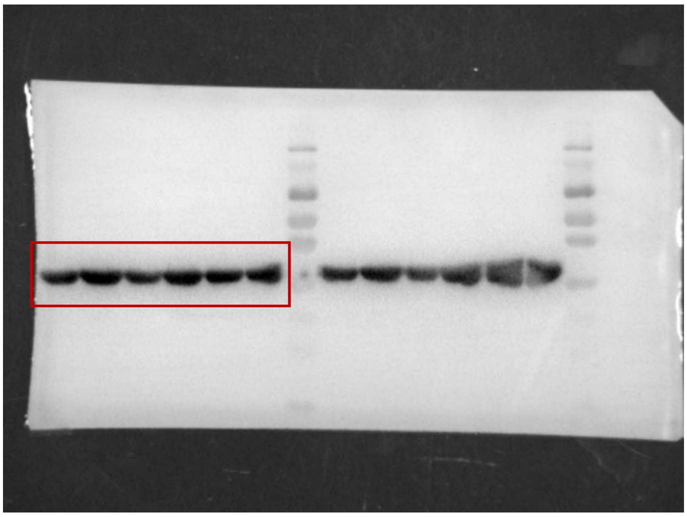

Figure S2 C

1.STING

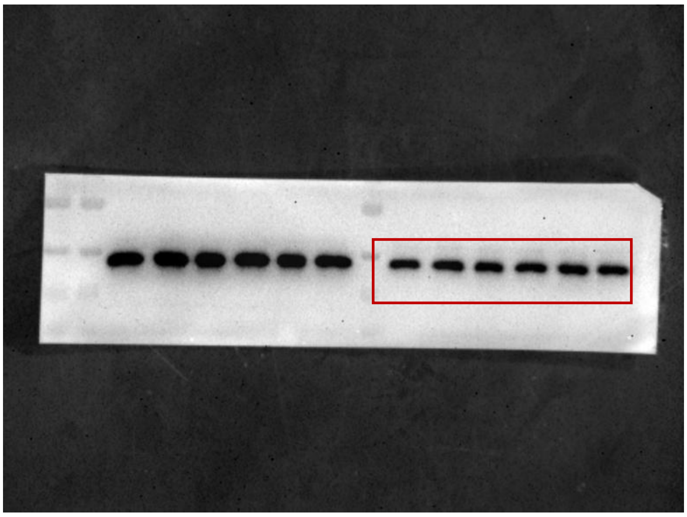

2.p-TBK1

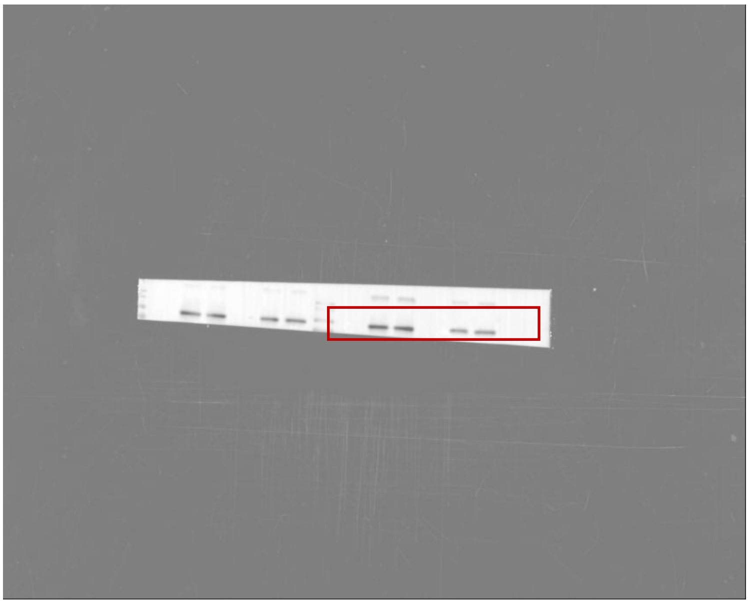

3.TBK1

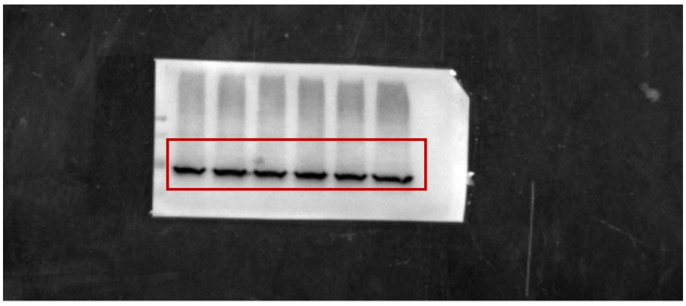

4.p-p65

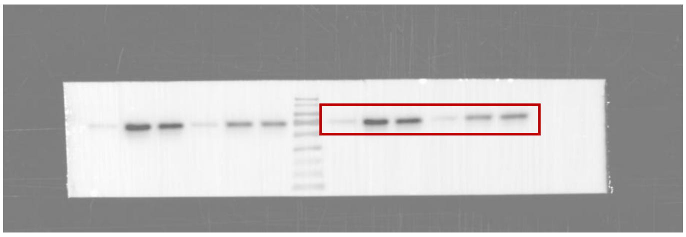

5.p65

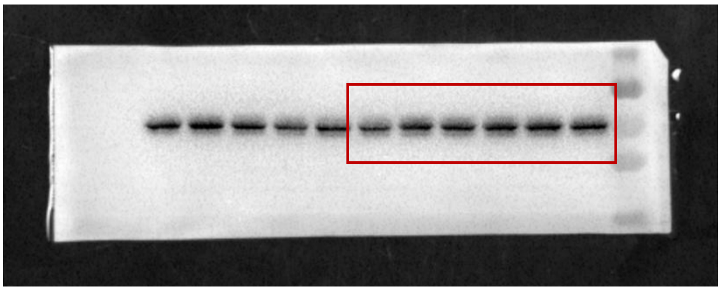

6.p-IRF3

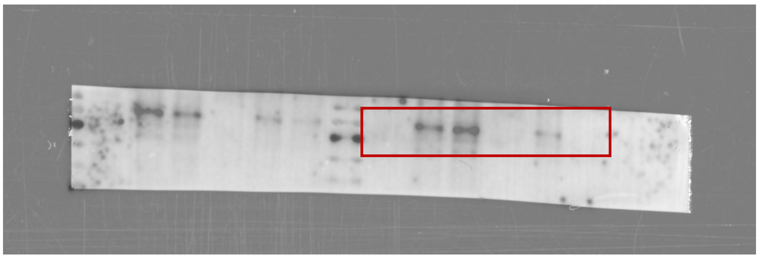

7.IRF3

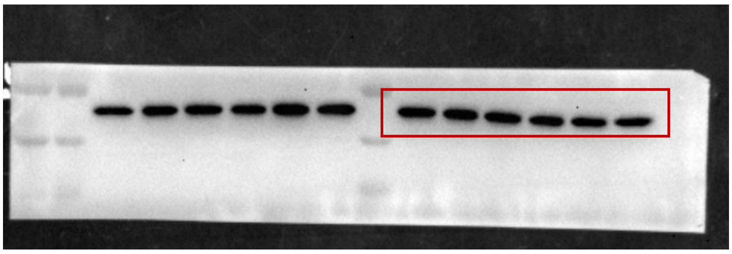

8. β-actin

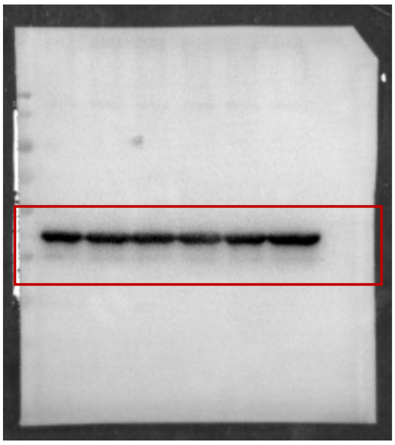

Figure S3 A

1.IP:HA-Ub

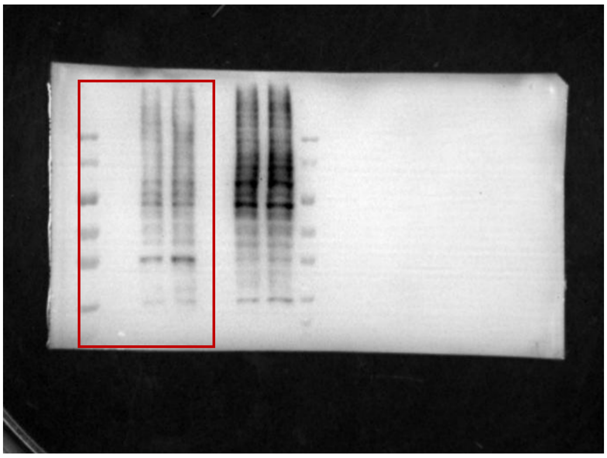

2.IP:Flag-STING

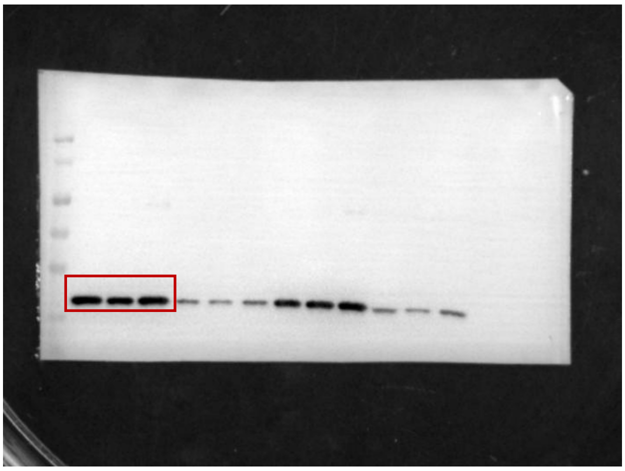

3. Input:HA-Ub

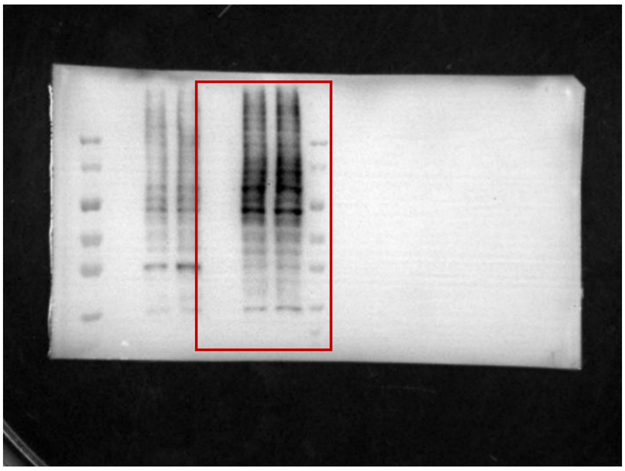

4.Input: Flag-STING

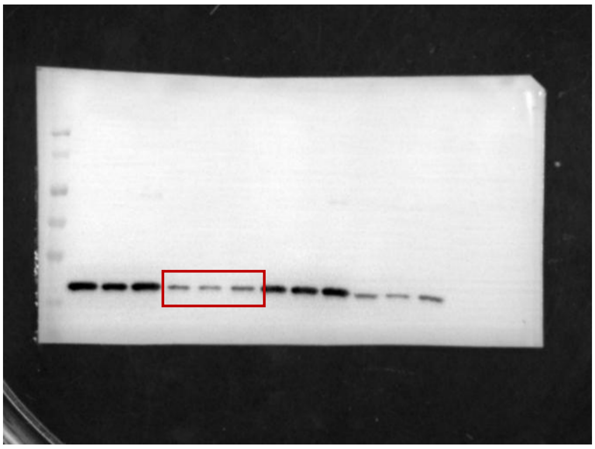

5.Input: Myc-DTX2

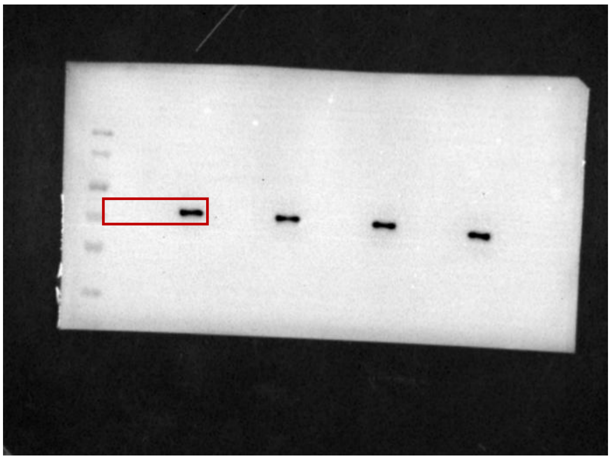

6.Input:β-actin

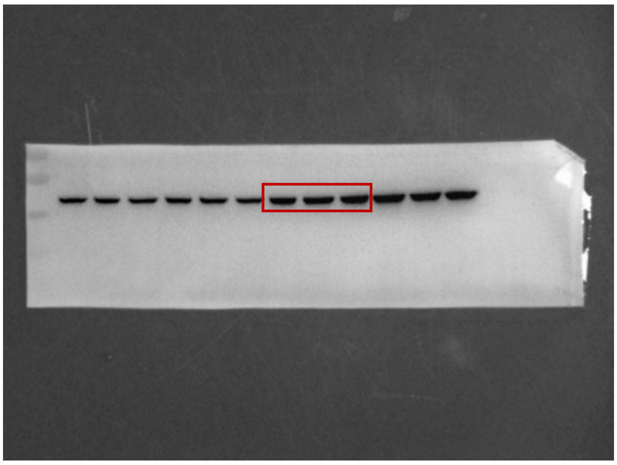

Figure S3 B

1. IP:HA-Ub

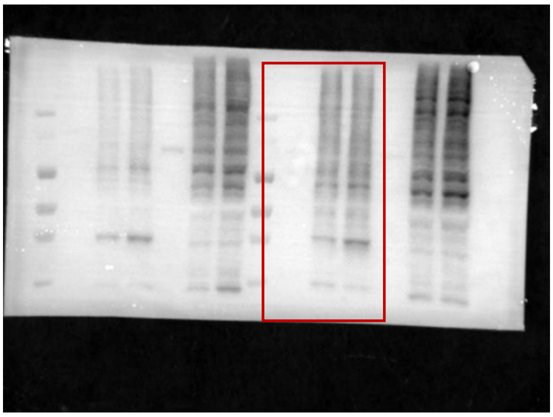

2.IP:Flag-STING

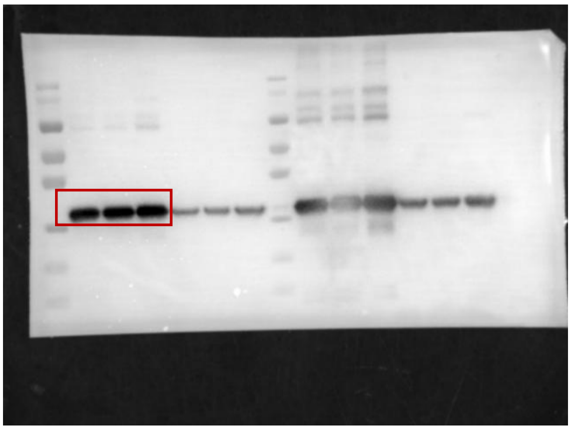

3. Input:HA-Ub

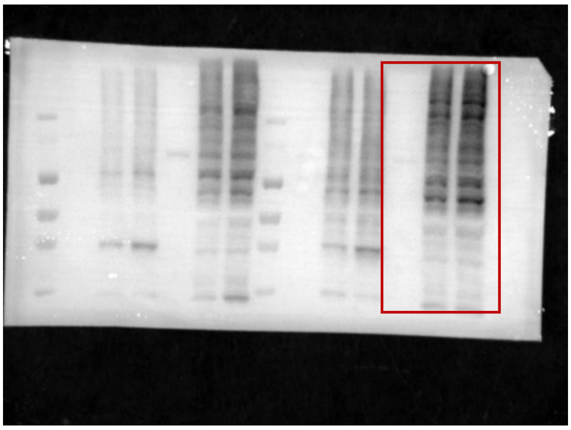

4.Input: Flag-STING

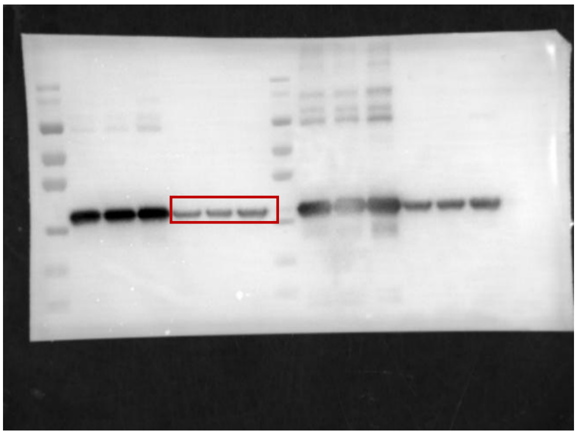

5.Input: Myc-DTX2

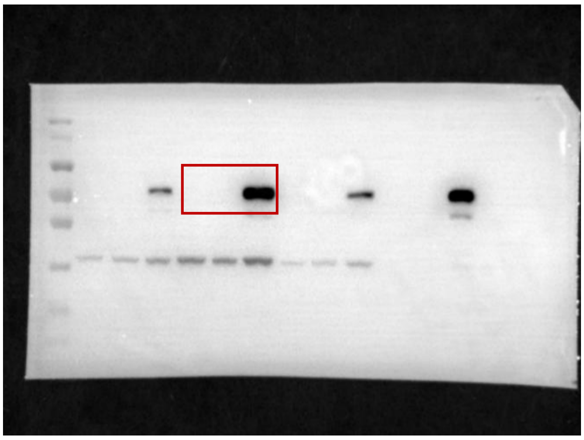

6.Input:β-actin

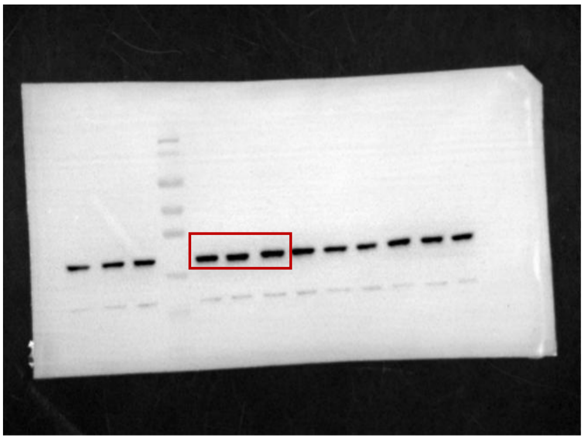

Figure S3 C

1.IP:HA-Ub

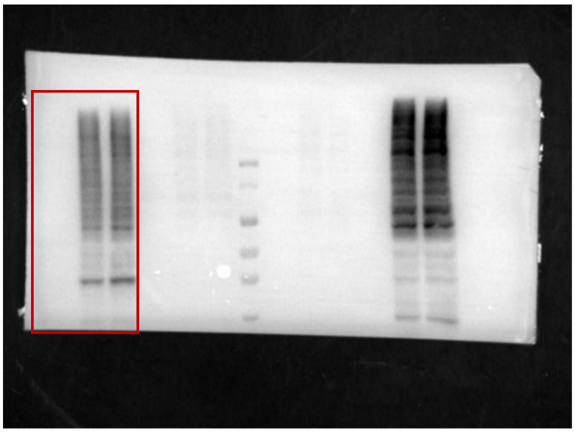

2.IP:Flag-STING

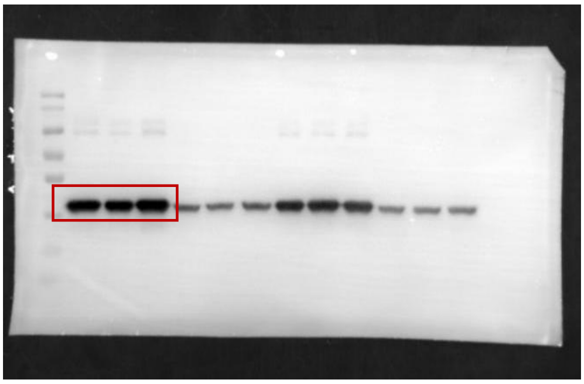

3. Input:HA-Ub

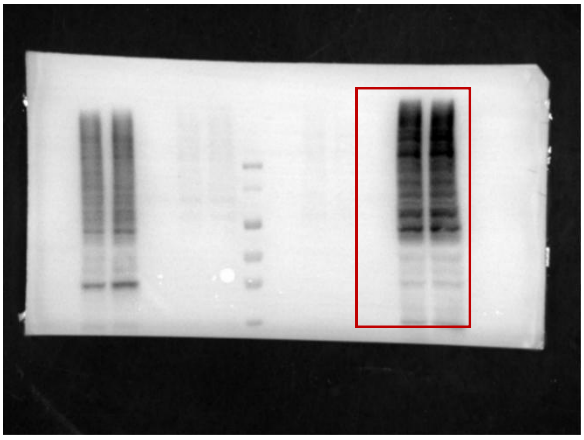

4.Input: Flag-STING

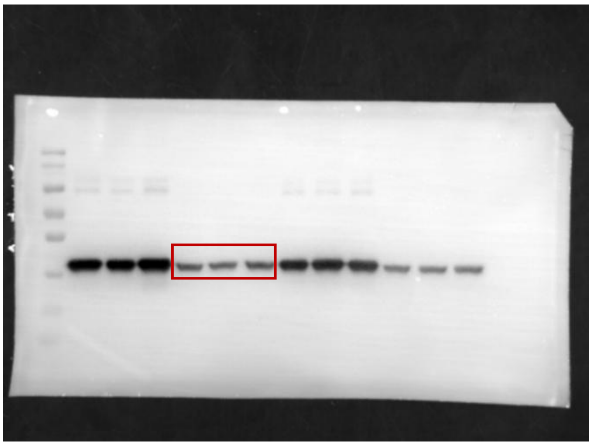

5.Input: Myc-DTX2

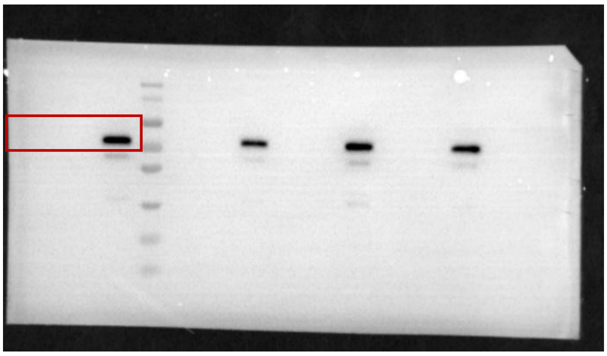

6.Input:β-actin

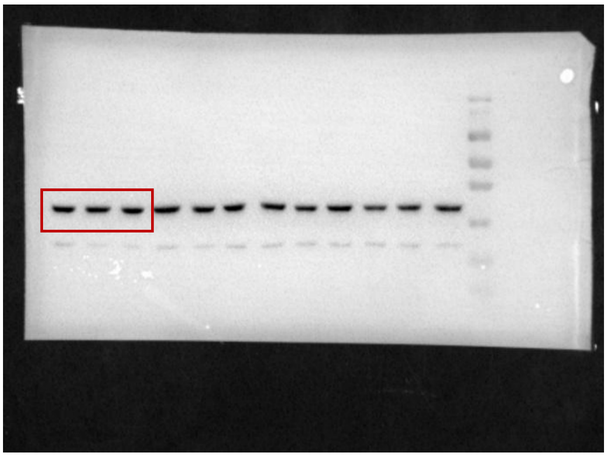

Figure S3 D

1.IP:HA-Ub

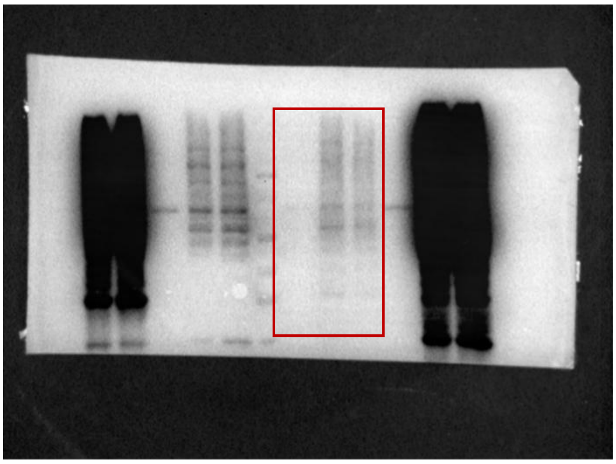

2.IP:Flag-STING

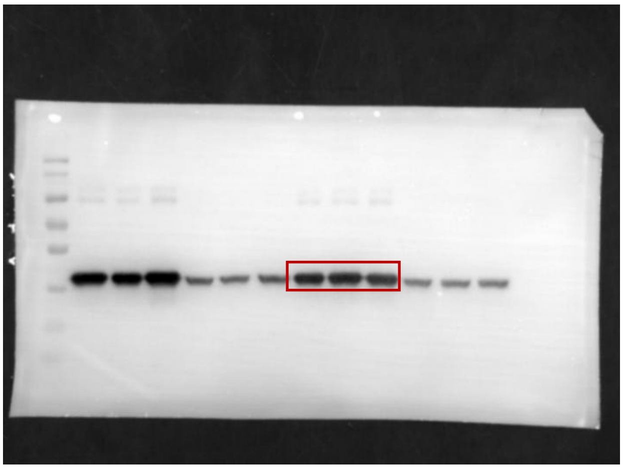

3. Input:HA-Ub

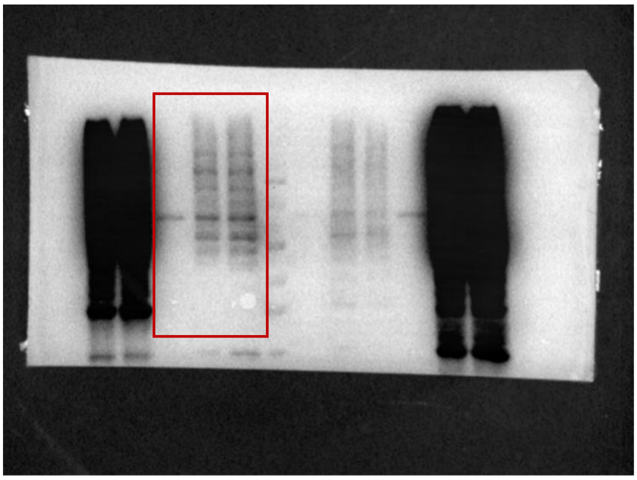

4.Input: Flag-STING

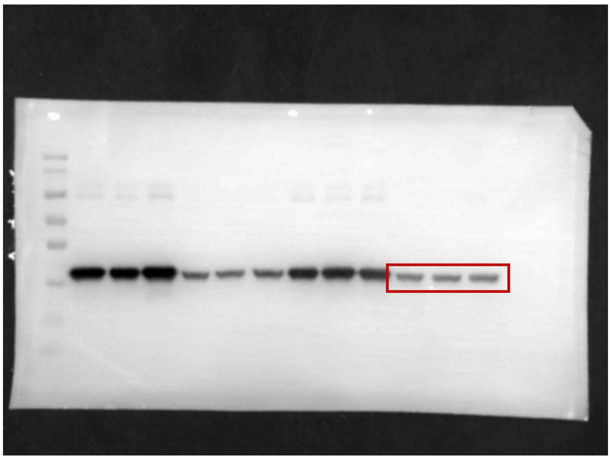

5.Input: Myc-DTX2

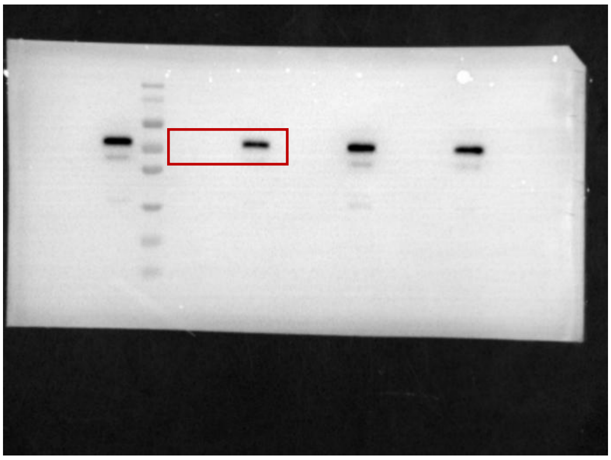

6.Input:β-actin

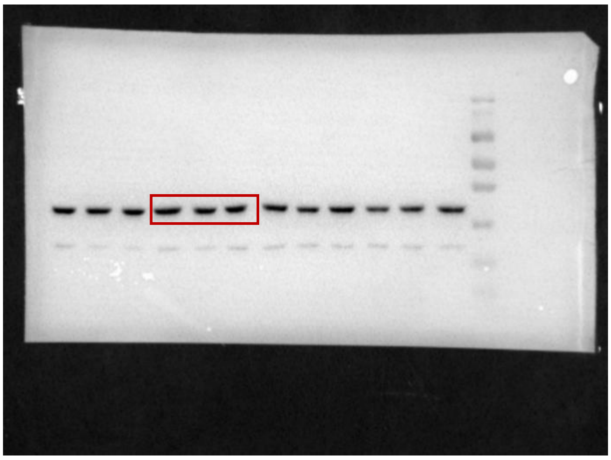

Figure S3 E

1.IP:HA-Ub

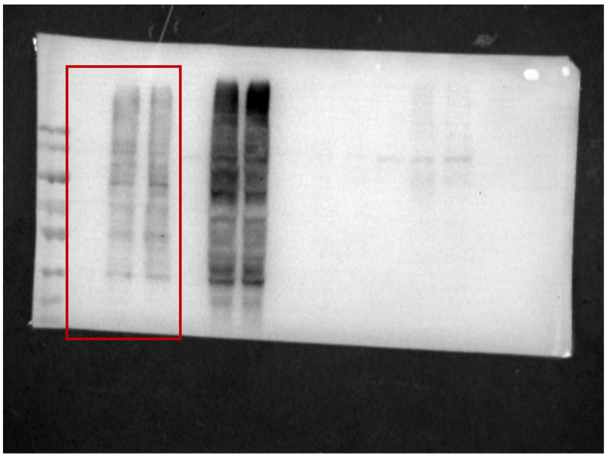

2.IP:Flag-STING

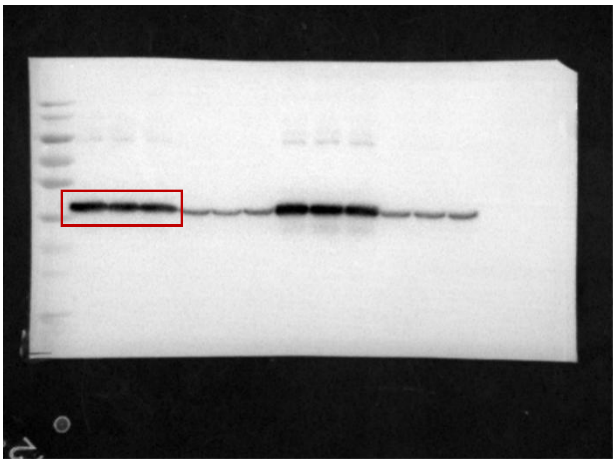

3. Input:HA-Ub

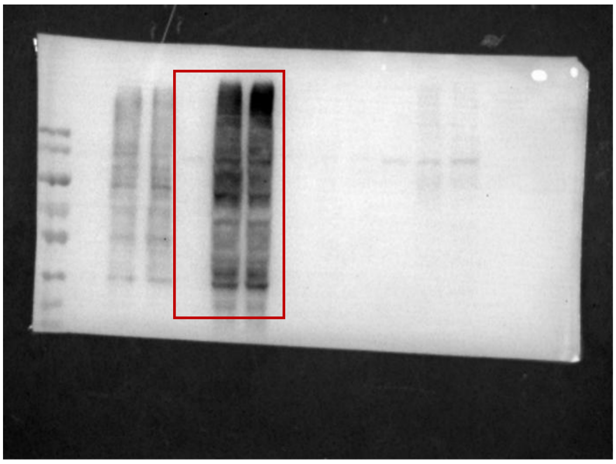

4.Input: Flag-STING

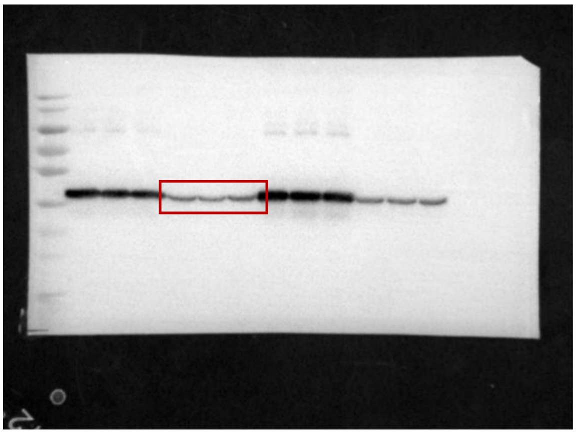

5.Input: Myc-DTX2

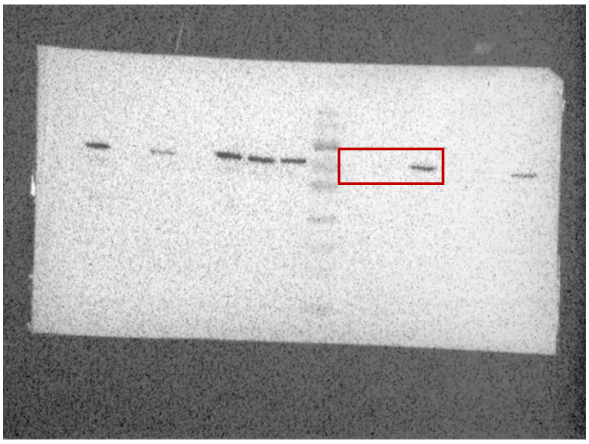

6.Input:β-actin

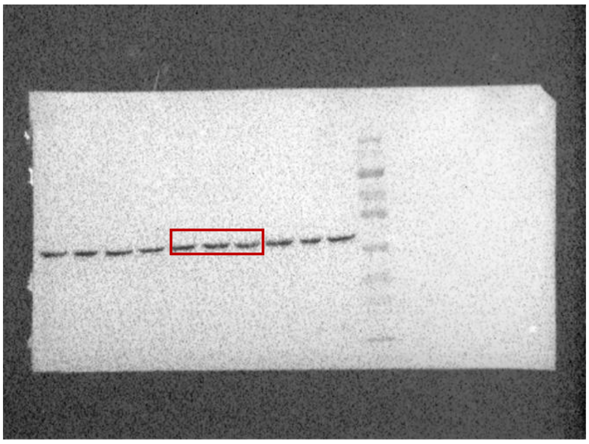

Figure S3 F

1.IP:HA-Ub

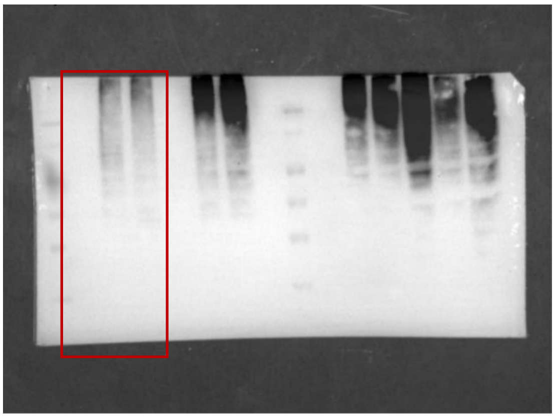

2.IP:Flag-STING

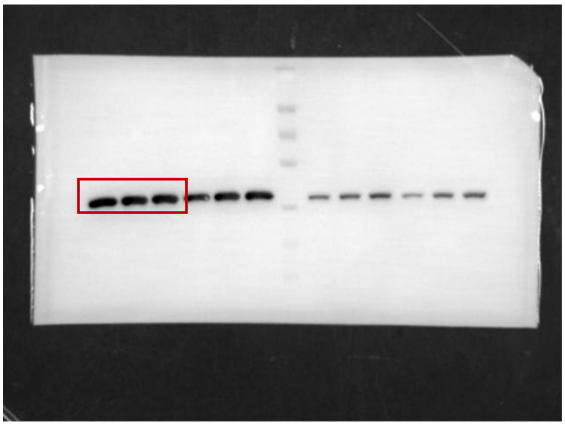

3. Input:HA-Ub

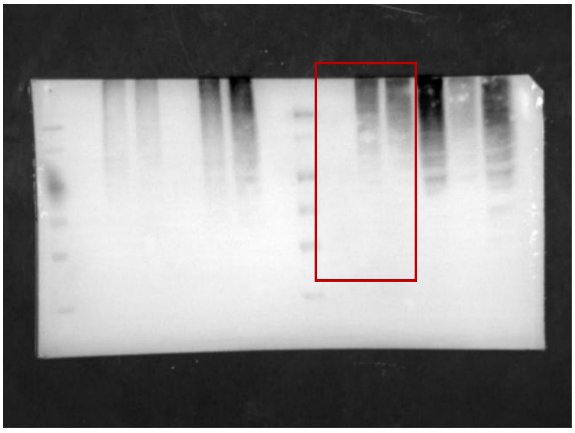

4.Input: Flag-STING

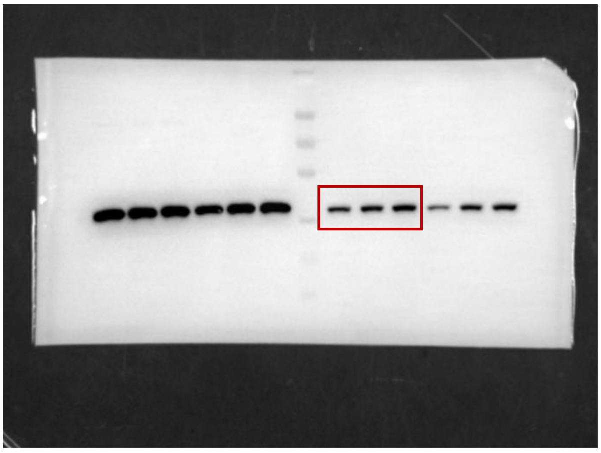

5.Input: Myc-DTX2

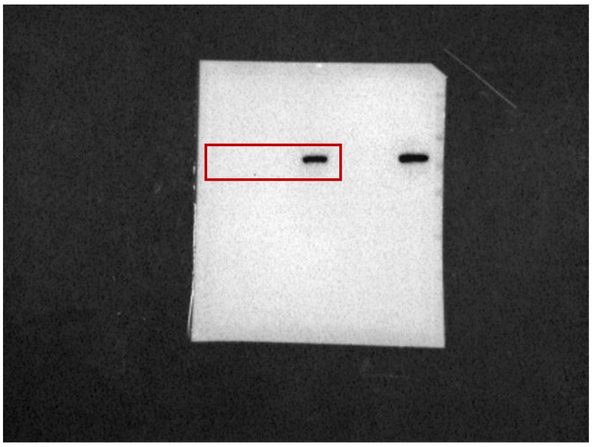

6.Input:β-actin

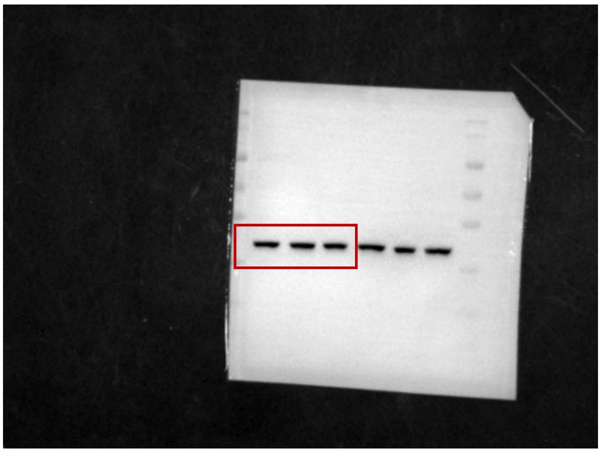

Fig S3 G

1.IP:Ub

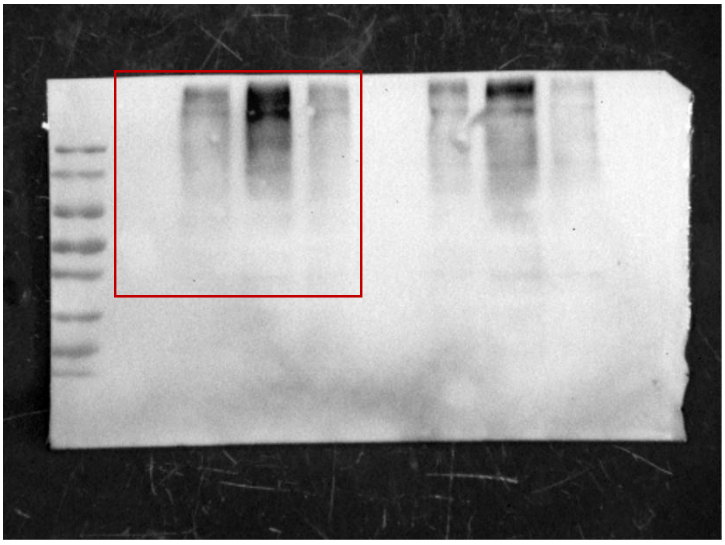

2.IP:STING

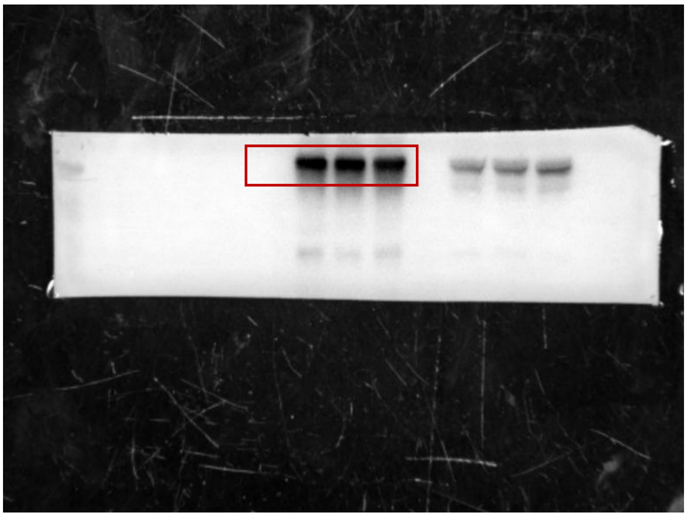

3.Input:Ub

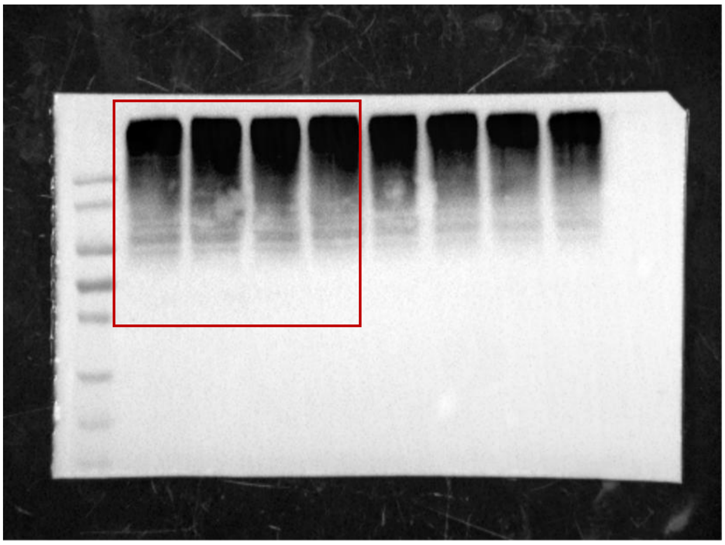

4.Input:STING

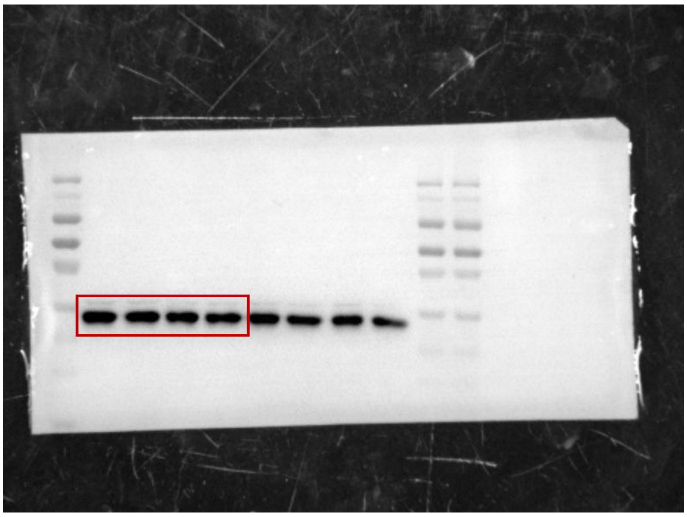

5.Input: Myc

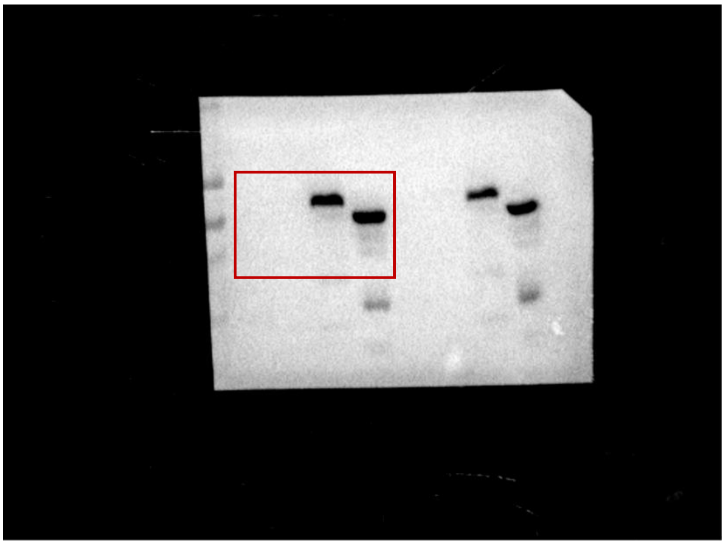

6.Input: β-actin

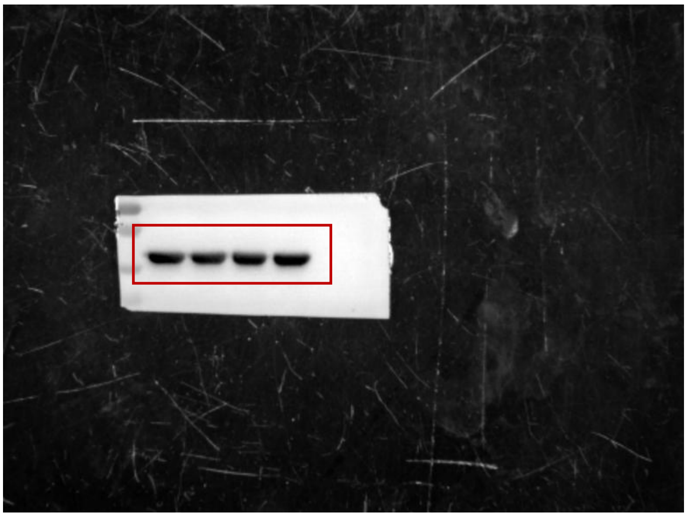

Fig S4 C

1.STING

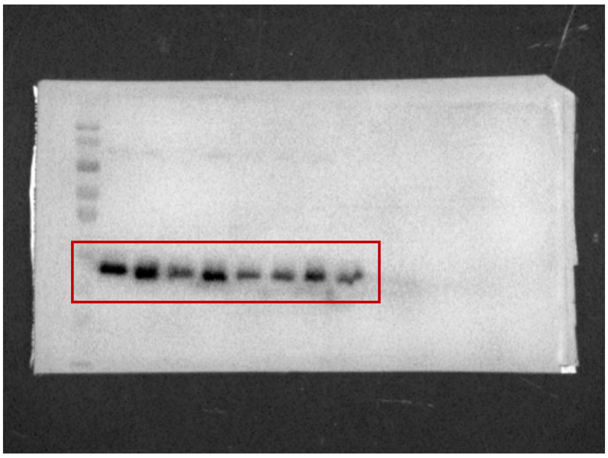

2.GM130

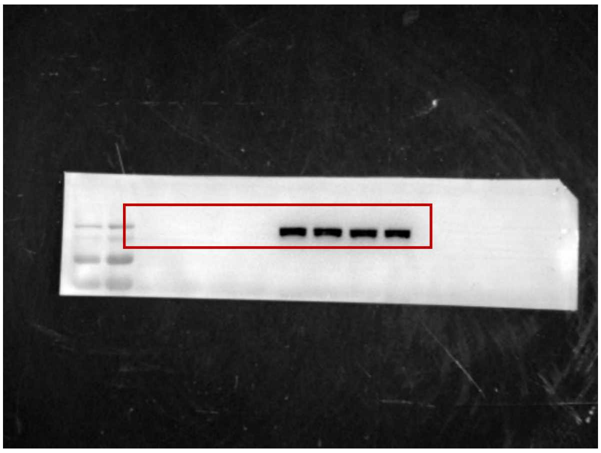

3.ERp72

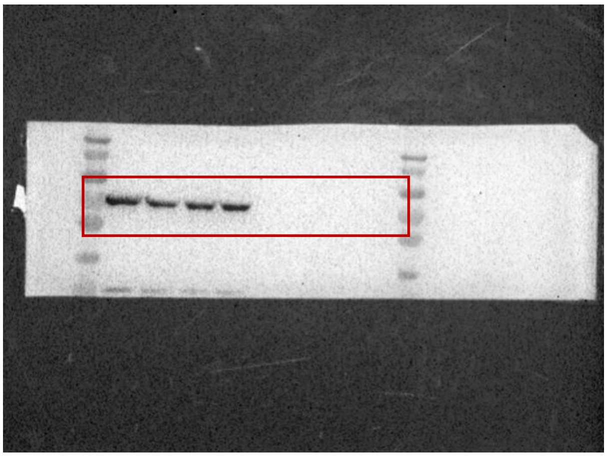

Fig S5 A

1.IP:Flag

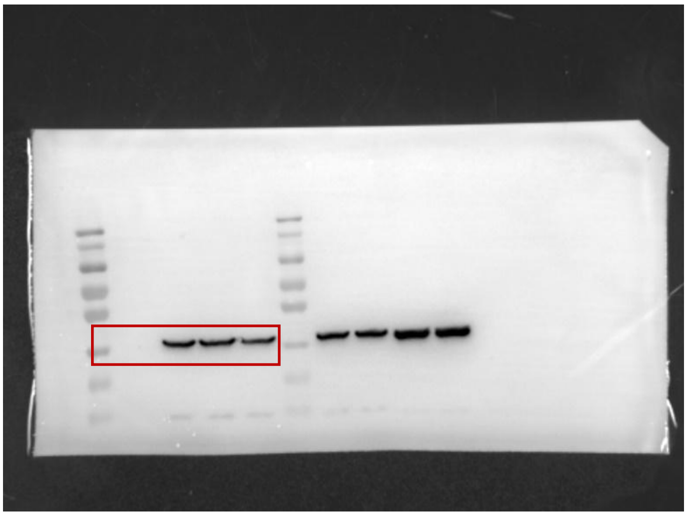

2.IP:Myc

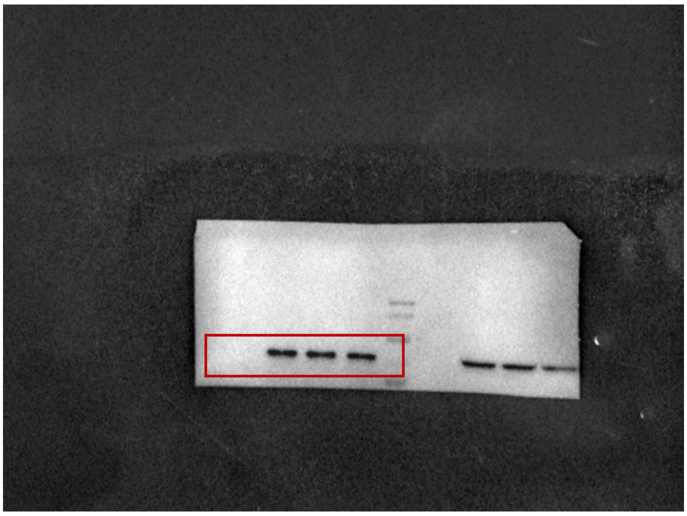

3.Input:Flag

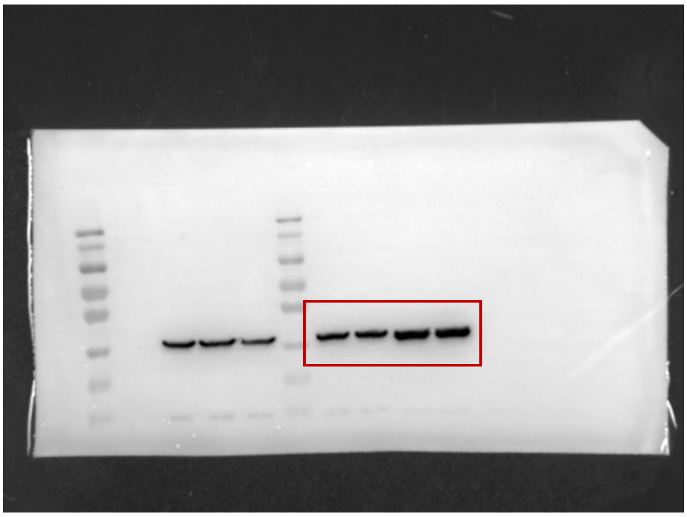

4.Input:Myc

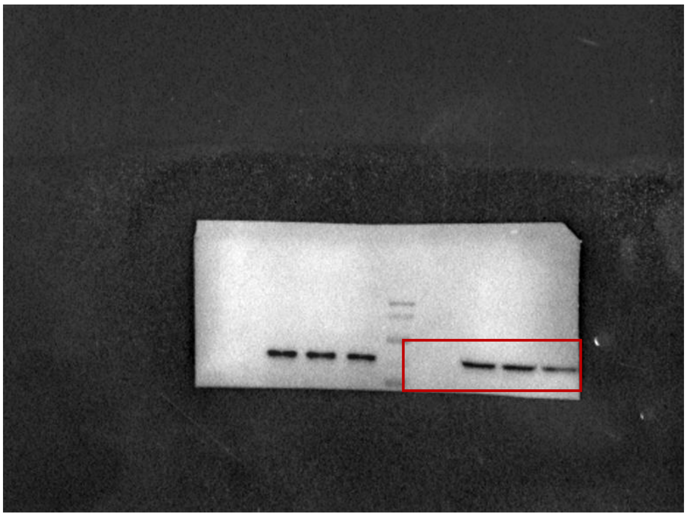

5.Input: β-actin

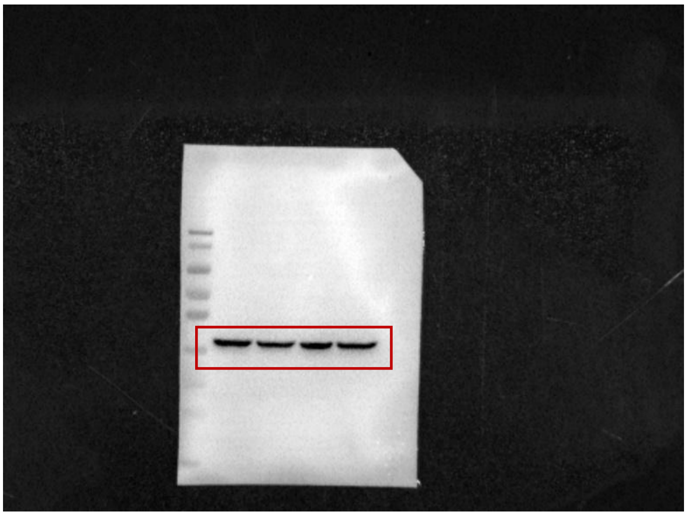

Fig S5 C

1.STING

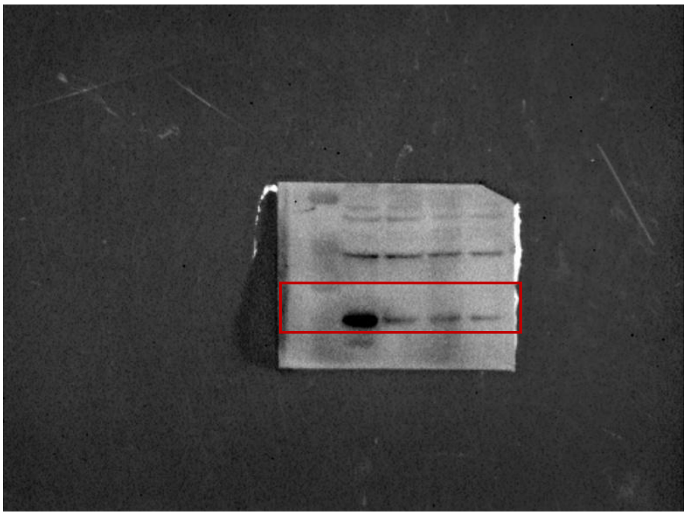

2.β-actin

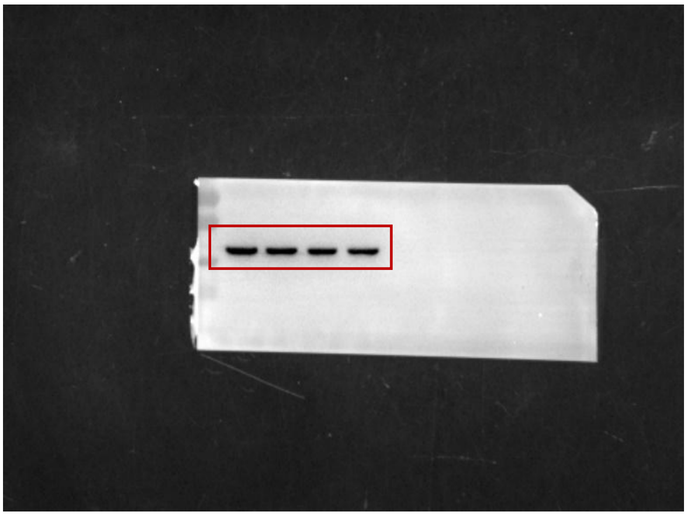

Fig S5 E

1.STING

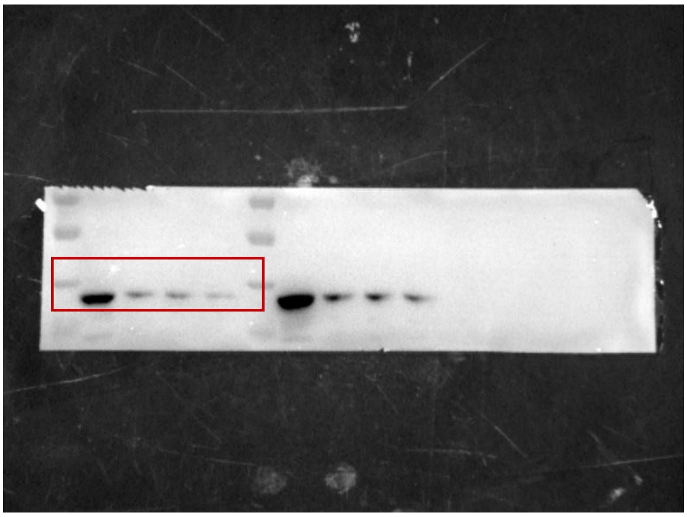

2.β-actin

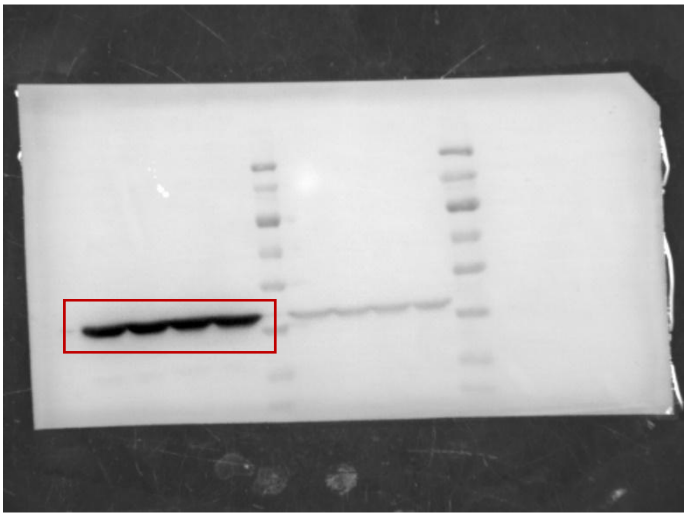

Fig S8 A

1.Myc-DTX2

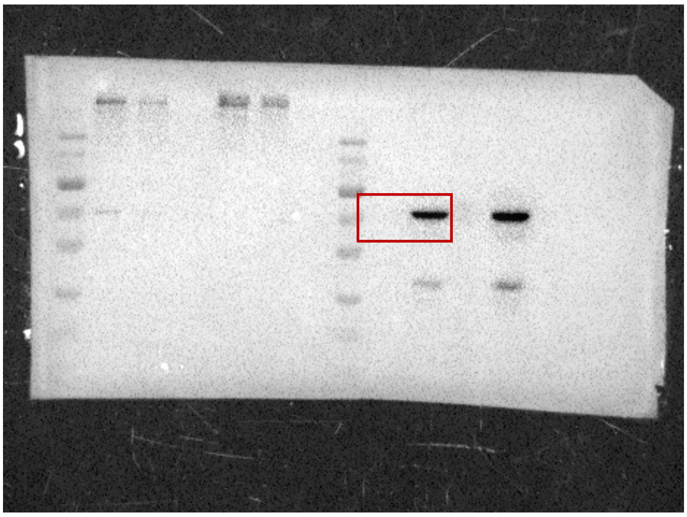

2.β-actin

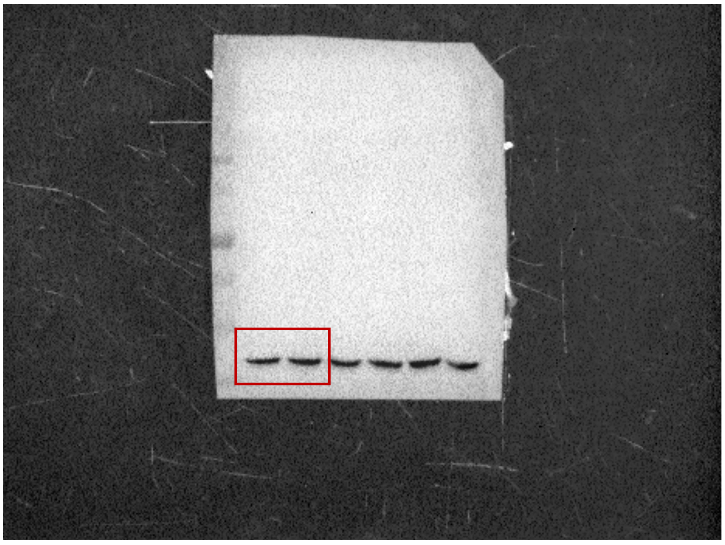

Fig S8 B

1.Myc-DTX2

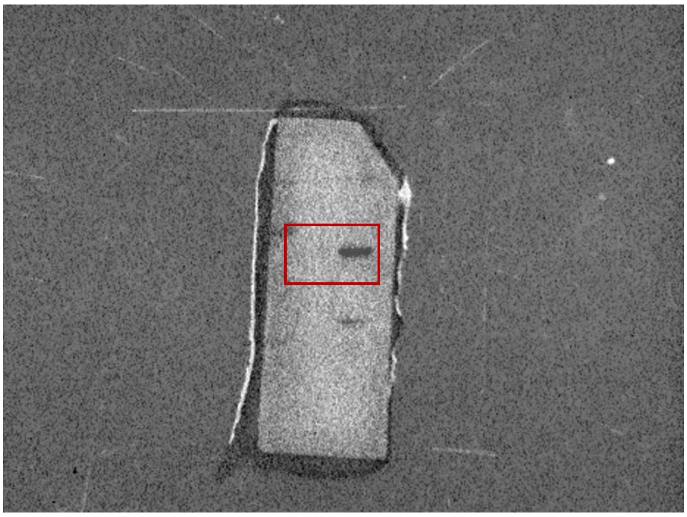

2. β-actin

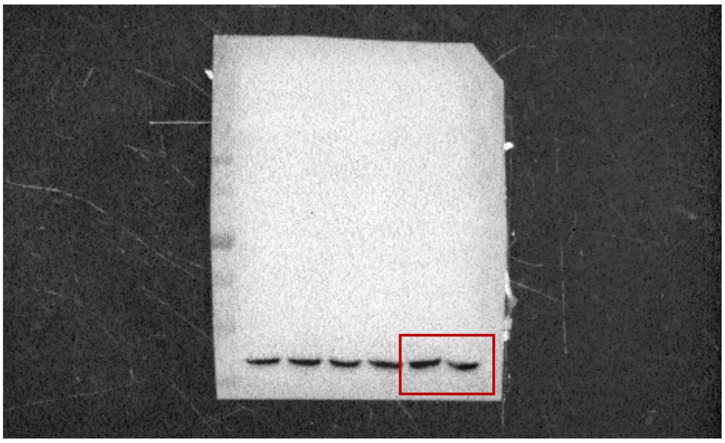

Fig S8 C

1.Myc-DTX2

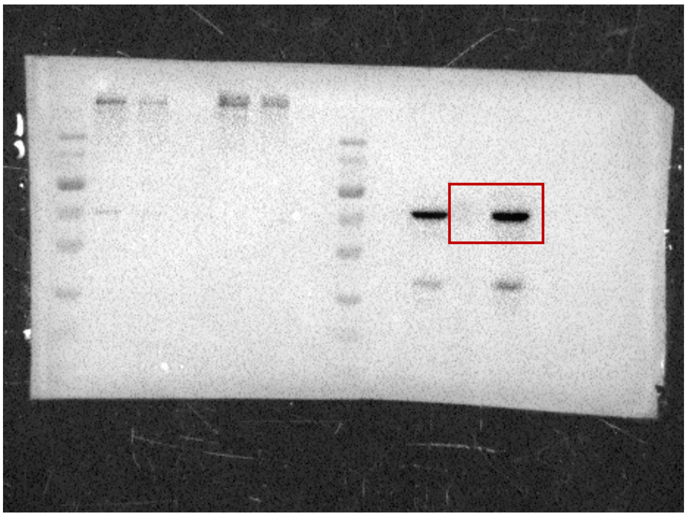

2. β-actin

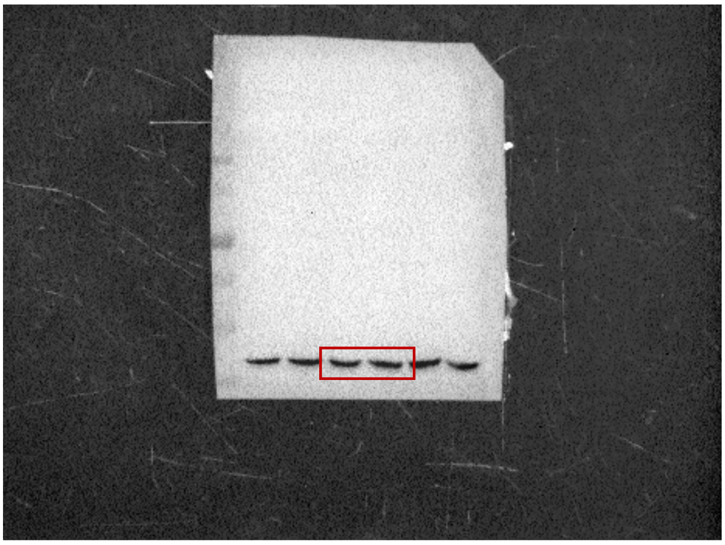

Fig S8 G

1.STING

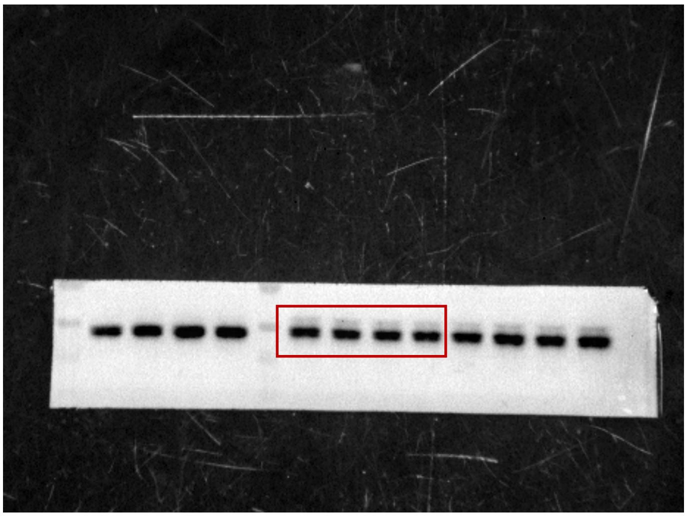

2.p-TBK1

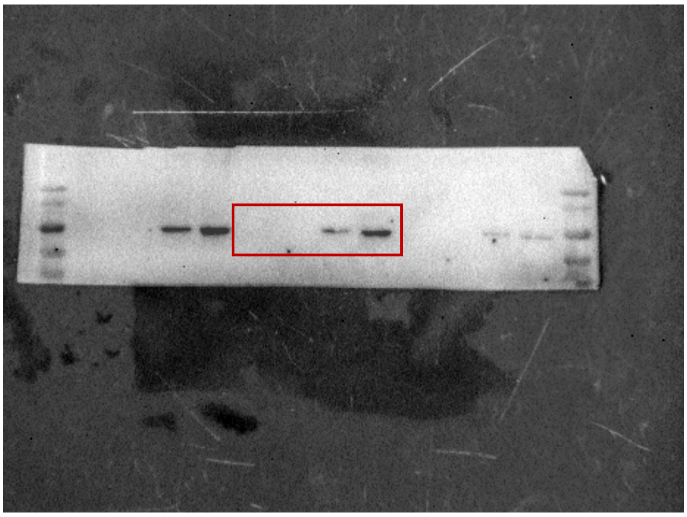

3.TBK1

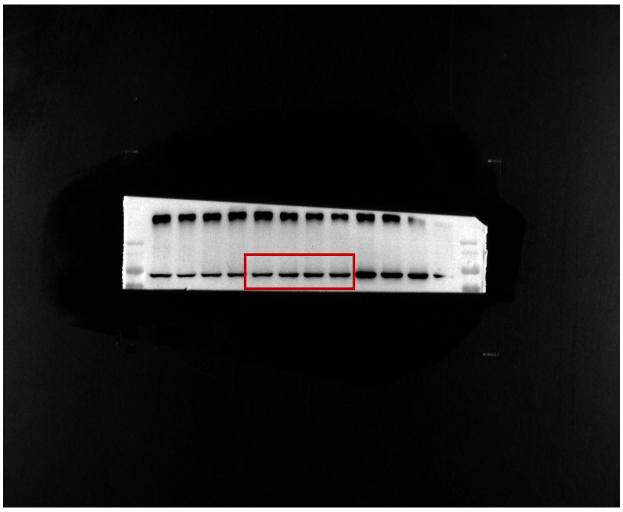

4.p-IRF3

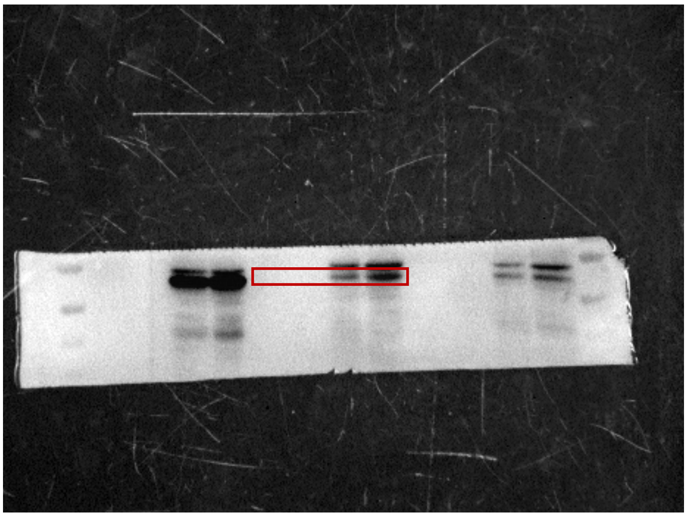

5.IRF3

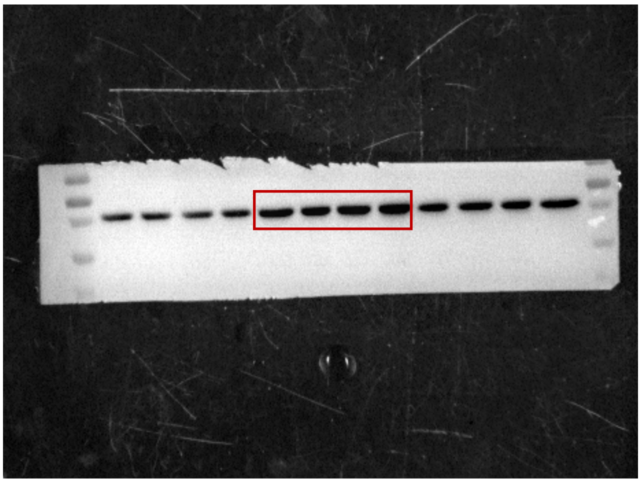

6.β-actin

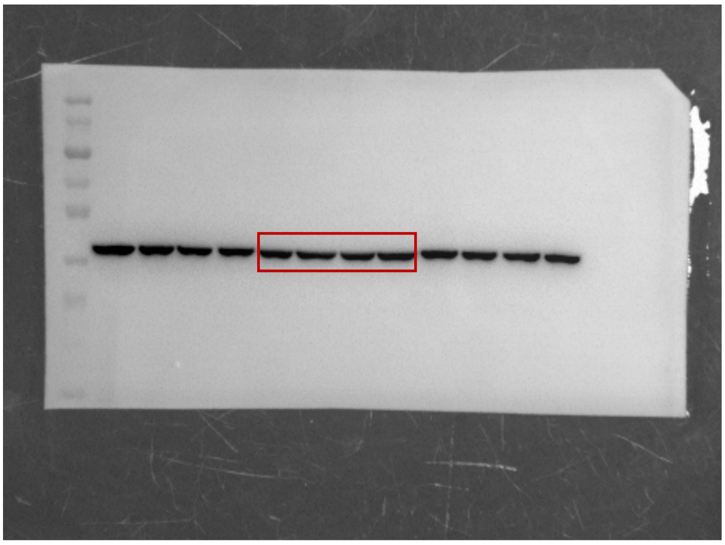

Supplement: Supplementary file 3 — Western blots [file 41419_2026_8659_MOESM3_ESM.pdf]
